# Tuning optical properties and local lone-pair off-centering in “hollow” FA_1−*x*_{*en*}_*x*_Pb_*η*−*y*_Sn_*y*_Br_3_ perovskites

**DOI:** 10.1039/d5sc01841b

**Published:** 2025-10-31

**Authors:** Adam Balvanz, Anastasia Pournara, Robert P. Reynolds, Patricia E. Meza, Christos D. Malliakas, Jared D. Fletcher, Ram Seshadri, Vinayak P. Dravid, Mercouri G. Kanatzidis

**Affiliations:** a Northwestern University Department of Chemistry Evanston IL 60208 USA m-kanatzidis@northwestern.edu; b Northwestern University Department of Materials Science and Engineering Evanston IL 20208 USA; c Materials Department and Materials Research Laboratory, University of California Santa Barbara CA 93106 USA; d Materials Science Division, Argonne National Laboratory Lemont IL 60439 USA

## Abstract

Hollow metal halide perovskites, while a relatively recent discovery, have already been shown to push the boundaries of tuneability within an inherently limited compositional space. Here, we further expand our knowledge of these complex materials demonstrating the ability to control optical properties and local structure, notably lone-pair induced off-centering. We focus on the mixed metal FA_1−*x*_en_*x*_Pb_*η*−*y*_Sn_*y*_Br_3_ hollow perovskites. These compounds have wide ranging optical gaps from 1.9 eV (deep red) to 2.6 eV (light yellow), combining the anomalous bandgap red-shifting of Pb/Sn mixing with the blue-shifting effects of the organic substitution. Average and local structural studies employing single crystal X-ray diffraction and total X-ray scattering pair distribution function analyses respectively suggest strong incoherent off-centering distortions that are locally correlated with Sn concentration. The inclusion of ethylenediammonium dications appears to regulate metal off-centering, opening new opportunities of research into this phenomenon.

## Abstract 2

The synthesis, structural, and optoelectronic characterization of the family of FA_1−*x*_en_*x*_Pb_*η*−*y*_Sn_*y*_Br_3_ hollow metal halide perovskites highlighting the interplay between the local structure dynamics and optical properties.

## Graphical abstract

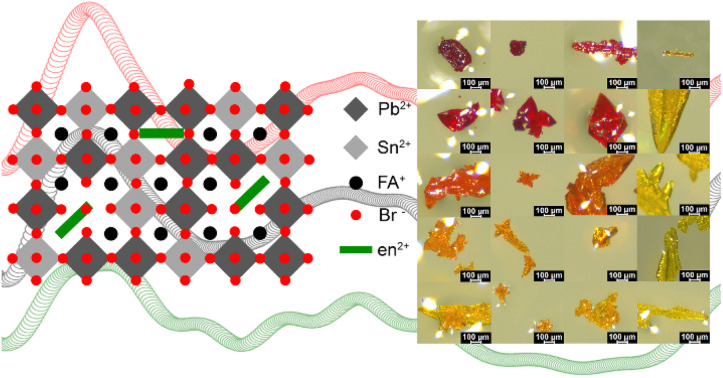

## 1 Introduction

Metal halide perovskites (MHPs) are a promising class of optoelectronic materials with wide-ranging applications in devices that employ high-performance semiconductors: photovoltaics (including single^1–5^ and multijunction^6–11^ devices), radiation detectors,^12–16^ and light-emitting diodes (LEDs)^17–20^ to name a few. These compounds are highly absorbing, direct-gap semiconductors with high charge carrier mobilities^21^ that can be easily synthesized through low-cost synthetic methods and starting materials.^22^ Unlike conventional semiconductors such as silicon and gallium arsenide, MHPs display a remarkable resiliency to electronic defect states, making them a focal point for scientific research efforts and technological advancements as a cost-effective alternative.^23–25^ In addition, compositions can be engineered to match a desired optical band gap (*E*_g_), with few exceptions, through simple chemical substitutions, accessing semiconductors with *E*_g_ ∼1.2–2.4 eV.^26–29^ With regard to photovoltaic applications, despite their rapid accent in power conversion efficiency, MHPs fundamentally suffer from two endemic pitfalls to their commercial viability.

A limiting feature of the MHP family, specifically the highest-performing three-dimensional (3D) variety, is the constrained compositional space. Defined as an AMX_3_ composition (where A and M are mono and divalent cations respectively and X is a monovalent anion) there are only three A-site cations (formamidinium (FA^+^), methylammonium (MA^+^), and Cs^+^) and limited M-site cations (Ge^2+^, Sn^2+^, Pb^2+^) and three X-site anions (Cl^−^, Br^−^, I^−^) that can form a perovskite framework (with some exceptions).^30,31^ These limitations are reflective of the geometric requirements to stabilize the 3D perovskite structure.^32,33^ If only considering compositions with potential applications, the M-site possibilities are narrowed further to Sn^2+^ and Pb^2+^ with other divalent M^2+^ having limited stability outside of a reducing environment. The initiative to reduce the reliance on Pb-containing devices due to Pb's toxicity^34^ can further narrow the field of usable MHP materials.

The ambient stability of MHPs is the second constraining factor, especially when considering the compounds with applications in solar cell technologies (the most widely pursued research area). The α-phases of FAPbI_3_ and CsPbI_3_ (*E*_g_ = 1.45 and 1.67 eV respectively),^22^ for instance, are highly moisture sensitive with both phases undergoing a moisture-assisted phase transition to their respective δ-phases after exposure to ambient conditions, while the Sn-bearing FASnI_3_/MASnI_3_ (*E*_g_ = 1.41 and 1.20 eV respectively)^22^ display high oxygen sensitivity with the Sn^2+^ oxidation state being unstable with respect to oxidizing to Sn^4+^. The hybrid materials are also susceptible to thermal degradation at relatively low temperatures, increasing the barrier to their commercialization. Some of these shortcomings have historically been addressed through dimensional reduction to a 2D perovskite lattice,^35^ but the expansion of potential compositions and improvements to stability come with significantly reduced photovoltaic conversion efficiencies.

Ideally, MHPs would permit a more expansive composition space, be free from instabilities when exposed to ambient conditions, without compromising the favorable electronic structure characteristics afforded by its 3D architecture and be primarily Sn-based. The intriguing class of “hollow” metal halide perovskites (hMHP) has proven to be a step towards ideality by the incorporation of a “hollowing agent” (most commonly ethylenediammonium, en^2+^, although others have been reported)^36,37^ into the lattice by introducing it during the synthesis/film deposition. The hMHP family has been shown to drastically improve air stability and provide a secondary parameter to further tune the composition and optoelectronic properties of the resulting phase. These exotic compounds are characterized by their progressively reduced crystal density, blue shifting band gap/photoluminescence (PL) emission, and aperiodic substitution of the hollowing agent causing lattice expansion.^38,39^ hMHPs have already been shown to be effectively incorporated into PV devices, improving device stability.^40–42^

Here, we build upon our previous works in the hMHP family by pushing the boundary into the compositionally complex FA_1−*x*_en_*x*_Pb_*η*−*y*_Sn_*y*_Br_3_ (*η* = 1–0.5*x*; *x* = 0–0.36; *y* = 0.17–1) family to explore the combination of the band-bowing effects exhibited by Pb/Sn MHP alloys^26^ and the improved stability and band gap widening of the hollow counterparts. We report the synthesis, structural, and optical property characterization of twenty compounds within this phase space, rigorously examining the substitution patterns of ethylenediammonium dications through the combination of single crystal X-ray diffraction, quantitative ^1^H NMR, Pb/Sn elemental analysis, and experimental crystal density measurements. We find that the en^2+^ substitution does not substantially impact the Pb/Sn ratio in the resulting compound from a synthetic perspective and suggest an alternative substitution pattern producing a formula described as FA_1−*x*_en_*x*_Pb_*η*−*y*_Sn_*y*_Br_3_ (*η* = 1–0.5*x*) which differs from the previously reported A_1−*x*_en_*x*_M_1−0.7*x*_X_3−0.4*x*_.^38,39^ The optical characterization reveals the sweeping ability to produce materials with optical gaps between 1.9 and 2.6 eV, combining the well-known anomalous redshifting effects of Pb/Sn mixing and progressively blue-shifting characteristics of hMHPs. Although the typically robust photoluminescent properties of MHP are notably absent from these materials, its absence is reconciled with the experimental 1-dimensional atomic pair distribution function which shows the presence of local structure M^2+^ off-centering known to drastically decrease PL intensity. However, the addition of en^2+^ was remarkably found to have the effect of reducing the levels of these distortions on the local level, though proved to be insufficient with respect to reviving the PL. This study highlights a methodology for producing compositionally and optically tunable MHP materials for further study and opens a new pathway for addressing M-site off-centering which is critical for the adoption of Pb-free devices.

## 2 Materials and methods

### 2.1 General synthetic procedures for the FAPb_1−*y*_Sn_*y*_Br_3_ compositions

Each of the “non-hollow” compounds, referred to as the parent materials in all future discussions, were synthesized using a typical precipitation reaction from hydrobromic acid (HBr). Desired quantities of Pb(CH_3_COO)·3H_2_O (Sigma Aldrich; 98 wt%) and SnCl_2_·2H_2_O (Sigma-Aldrich; 98 wt%) were massed and added to a 20 mL scintillation vial alongside a magnetic stir bar. Desired volumes of HBr were then added directly to the vial and the reaction was moved to a hotplate set to 120 °C. The solids were allowed to dissolve in the acid. 1 mL of hypophosphorous acid (H_3_PO_2_; Sigma Aldrich; 50 wt% in H_2_O) was also added to the reaction as a reducing agent, since the Sn^2+^ oxidation state is metastable, readily oxidizing to Sn^4+^. After allowing the solid to dissolve completely, formamidine acetate (FACH_3_COO; TCI; >98 wt%) was added to the hot solution upon which a solid precipitated from the solution. Following the addition, the hotplate temperature was increased to 200 °C and the reaction was kept under constant stirring until all of the solids were once again dissolved. Once a clear solution was achieved, the hotplate was turned off and the stirring was discontinued. The solution was left on the hotplate to cool to room temperature. The crystals of the parent materials could then be isolated from the solution as light to deep red crystals depending on the composition. For specific synthetic conditions used for each compound, see the SI.

### 2.2 General synthetic procedures for the FA_1−*x*_en_*x*_Pb_*η*−*y*_Sn_*y*_Br_3_ compositions

The synthesis of the hollow analogs follows a similar procedure to the parent compounds with minor deviations to add the additional ethylenediammonium (en^2+^) cation to the reaction solution. Desired quantities of Pb(CH_3_COO)·3H_2_O (Sigma-Aldrich; 98 wt%) and SnCl_2_·2H_2_O (Sigma-Aldrich; 98 wt%) were massed and added to a 20 mL scintillation vial alongside a magnetic stir bar. Volumes of HBr were then added directly to the vial and the reaction was moved to a hotplate set to 120 °C. The solids were allowed to dissolve in the acid while in a separate vial, 1 mL of H_3_PO_2_ was added after which desired volumes of ethylenediamine (en) were added dropwise to protonate both the primary nitrogen heads, forming the dication (en^2+^) in solution. Once the solids in the primary reaction vessel were dissolved, the en^2+^/H_3_PO_2_ solution was transferred to the hot reaction solution. After stirring was continued for ∼1 minute, FACH_3_COO (TCI; >98 wt%) was massed and added to the hot reaction solution accompanied by subsequent precipitation of the perovskite phase. The temperature of the hotplate was then increased to 200 °C, allowing any remaining precipitate to dissolve, creating a clear solution. Once the precipitate was completely dissolved, the hotplate was turned off and the stirring was discontinued. The reaction was left on the hotplate to cool to room temperature naturally. Once the solid product precipitated, and the solution reached room temperature, the perovskite phase was extracted from the solution and dried in an inert atmosphere. Fast extraction of the perovskite phase was critical, especially for reactions with the highest concentration of en^2+^ since an en^2+^-rich phase can precipitate as a white solid if not diligently monitored. For specific synthetic conditions for each phase, the reader is referred to the SI for the specific compounds of interest. The identity of the bulk product was also verified through air-free powder diffraction experiments (Fig. S14–S17).

### 2.3 Drying of bulk perovskite crystals

For further testing on the bulk products, the materials were thoroughly dried. Following precipitation from the reaction solution, the crystals were separated from the mother liquor through vacuum filtration and then dried through either of the following methodologies.

#### 2.3.1. Vacuum drying

Crystallites were placed inside a fused silica tube (sealed at one end) and placed under vacuum (pressures less than ∼10^−2^ mtorr) for 12–48 hours. Once dried, the tube was quickly transferred to a nitrogen-filled glovebox for storage.

#### 2.3.2. Purging nitrogen

Once extracted, the sample was placed inside a custom 9/11 mm (inner diameter/outer diameter) fused silica tube sealed at one end. The tube was narrowed in the middle so a filter could be placed halfway down. On the bottom, a hole was made so nitrogen could constantly flow down the length of the tube. The sample was rested on top of the filter and the crystals were left to dry for ∼12 hours.

### 2.4 Air-free powder X-ray diffraction (PXRD)

Dry crystallites were ground using an agate mortar and pestle inside a nitrogen-filled glovebox until the product was a powder-like consistency. The powder was then packed into an 8 mm metallic mask by using 2 layers of polyimide tape (one on each side of the mask). The PXRD data were then collected in a transmission geometry within the range of 2–60° 2*θ* (Cu K_α_1__) on a STOE-STADI-P powder diffractometer utilizing an asymmetric curved germanium monochromator, a Cu K_α_1__ radiation source for diffraction (*λ* = 1.54056 Å) and a MYTHEN2 1K DECTRIS one-dimensional silicon strip detector. Before the data collection, the diffractometer was calibrated against a NIST Silicon standard (640d) prior to diffraction.

### 2.5 Single crystal X-ray diffraction

Selected compounds were structurally characterized through single crystal X-ray diffraction through the following process. The crystals were isolated and then placed under Paratone-N oil on a glass slide which protects the surface of the crystal from potential oxidation. Suitable crystals were then selected under an optical microscope, cut to size, and then transferred to either a XtaLAB Synergy diffractometer equipped with a microfocus sealed X-ray PhotonJet Mo K_α_ radiation source (*λ* = 0.71073 Å) and a Hybrid Pixel Array Detector (HyPix) or a STOE StadiVari diffractometer which uses an Ag K_α_ (*λ* = 0.560834 Å) X-ray A-MiXS source for diffraction and a Dectris Platus3 R CdTe 200K Hybrid Photon-Counting Detector. The data collected were reduced within either the CrysAlisPro (Rigaku) or X-Area software packages (STOE), using an empirical absorption correction. X-area utilizes LANA to accomplish scaling and outlier rejection.^43^ The resulting reflection and instrument files were imported into either Jana2006 (ref. 44) or Olex2.^45^ The data imported to Olex2 was solved through the intrinsic phasing method as implemented in SHELXT^46^ and further refined on *F*^2^ using ShelXL.^47^ The data imported to Jana2006 was solved through the usage of SUPERFLIP^48^ and the model was refined on *F*^2^. Jana2006 was chosen for this purpose based on the increased flexibility in writing the necessary occupancy constraints.

Because the presence of en^2+^ in the material lacks long-range ordering average crystal structure was refined through the incorporation of vacancies on both the organic and metal sites. The FA^+^ vacancy was fixed based on findings from quantitative ^1^H NMR on the dissolved bulk sample and the molar ratio of Pb/Sn was fixed based on ICP-OES data.

### 2.6 Quantitative ^1^H nuclear magnetic resonance spectroscopy

NMR spectra were collected at room temperature using a Bruker Avance III 500 MHz spectrometer equipped with a BBO Prodigy probe and fitted with an autosampler. Chemical shifts (*δ*) are reported in parts per million (ppm) and coupling constants (*J*) are reported in hertz (Hz). Spectra recorded in DMSO-d_6_ are referenced to residual ^1^H DMSO signal (*δ* = 2.50 ppm) and reported relative to tetramethylsilane (TMS) at *δ* = 0.00. Spectra analysis, peak finding, and integration was performed using Bruker TopSpin software.

The samples were prepared by dissolving 45–60 mg of each compound in 0.5 mL of DMSO-d_6_ with maleic acid (*δ* = ∼6.20) included as a quantitative standard with either 0.111 or 0.094 M concentration, resulting in clear solutions on shaking. Each spectrum collected is provided in the SI along with the calculated integrations.

### 2.7 Inductively coupled plasma-optical emission spectroscopy (ICP-OES)

ICP-OES was performed on a computer-controlled (QTEGRA software) Thermo iCap7600 ICP-OES operating in axial view and equipped with an ESI SC-2DX PrepFAST autosampler. Each sample was acquired using 5 second visible exposure time and 15 second UV exposure time including 3 replicates. The spectral lines selected for analysis of Pb^2+^ were: 340.458, 324.270, 363.470, and 340.458 nm. The spectral lines selected for the analysis of Sn^4+^ were: 189.989, 283.999, and 242.949 nm.

### 2.8 Helium gas pycnometry

The true experimental density for each compound was determined through helium gas pycnometry using a Micromeritics AccuPyc II 1340 equipped with a 1 cm^3^ sample measuring cup and utilizing a triplicate measuring methodology. Each sample was thoroughly dried before measurement and kept in a vacuum desiccator prior to the experiment. The instrument was first calibrated using a spherical standard obtained from Micromeritics and then the calibration was tested by experimentally measuring the volume of an independent standard (purchased from Micromeritics). An acceptable deviation in the average volume of the second standard was deemed to be ±0.001 cm^3^. If the volume fell outside of the bounds, the instrument was calibrated once more. Densities were determined by adding a known mass of material to the measuring cup and determining the volume to 3 decimal places. The simple division then resulted in the density of the material. Theoretical densities were then calculated using the formula of each compound, considering contributions from ethylenediammonium substitution, and the corresponding unit cell volume from the single crystal refinement.

### 2.9 Experimental pair distribution function measurements on FA_1−*x*_en_*x*_Pb_*η*−*y*_Sn_*y*_Br_3_

#### 2.9.1. Non-hollow parent compounds

Fully dry non-hollow compounds were mechanically ground in an agate mortar and pestle until a uniform powder was received inside a nitrogen-filled glovebox. The powder was then sieved to obtain particle sizes of <53 µm and added to a 0.3 mm borosilicate capillary (purchased from Charles supper Co.) that was sealed at one end and then flame sealed under vacuum (pressures less than 10^−2^ mbar). Sieving the compounds was an effective way to ensure dense packing of the crystallites. Data was then collected on a Stoe StadiVari diffractometer which is equipped with an AXO Ag K_α_ (*λ* = 0.560834 Å) X-ray A-MiXS source and a Dectris Platus3 R CdTe 300 K hybrid photon counting detector for diffraction. The high resolution PXRD patterns were collected in the 2*θ* range of 1 to 160° over 5 frames using a 2 hour exposure time per frame. A 14 000 keV filter was applied to the detector to reduce contributions from anomalous scattering. Data for an empty capillary and air scattering was also collected for the purpose of providing a baseline. The images were individually integrated in GSAS-II^49^ where a collection on a NIST SRM 660c certified LaB_6_ standard was used to calibrate the instrumental parameters. After integration, the resulting frames were combined, manually scaled, and reduced to the *G*(*r*) in PDFgetX3.^50^ Further details about the calculations are provided in the SI. Here we note that the PDF curve for FASnBr_3_ was obtained as a part of our previous study,^51^ but is reexamined here within the context of the title system.

#### 2.9.2. Hollow compounds

Dry, bulk product of representative compositions of the hollow perovskite compounds were mechanically ground using a mortar and pestle inside of a nitrogen-filled glovebox and then sieved to particle sizes of <53 µm. The compounds were added to a vial and vacuum sealed in plastic for transportation to the ID31 beamline at the European Synchrotron Radiation Facility (ESRF) where the powders were stored in an argon-filled glovebox until measurement. Total scattering measurements were then conducted by adding the powder into cylindrical slots (∼1 mm thickness) between 2 Kapton windows in a high-throughput sample holder. Data were collected in a transmission geometry using an X-ray beam with an incident energy of 75.051 keV (*λ* = 0.16520 Å) and a Pilatus CdTe 2M detector with dimensions 1679 × 1475 pixels where the square pixels were 172 µm in length. To maximize the resolution, the beam was aligned to the corner of the detector. The background was subtracted through an additional measurement of empty sample holders and windows. For calibration of the instrumental setup, data was collected for a NIST SRM 660b LaB_6_ standard which was performed in the pyFAI software package. The resulting images were integrated applying flat-field geometry, solid-angle, and polarization corrections. The resulting powder patterns were then reduced to the *G*(*r*) through the PDFgetX3 software package.^50^

### 2.10 Reverse Monte Carlo atomic pair distribution function (PDF) analysis

In an effort to more conclusively analyze the local structure of these phases, reverse Monte Carlo simulations were conducted for the purposes of fitting the experimental *G*(*r*). High resolution powder X-ray diffraction data for selected compositions were imported into GSAS-II^49^ and were used as the basis for a Rietveld analysis using the experimentally determined average crystal structure as the basis structure. For the purposes of modeling, the FA^+^ cation, modeled as disordered in the single crystal X-ray diffraction analysis, was substituted for Mn to simplify the analysis. Reverse Monte Carlo fitting of three ranges of *r* were conducted to understand how the local distortions are averaged as longer-range ordering is considered. The low *r* cutoff was set to 2.5 Å while the upper limit was set to 6, 12, and 20 Å. These simulations were done within the FULLRMC software package^52,53^ using GSAS-II^49^ as a graphical interface. A 10 × 10 × 10 supercell approximation was used as the starting model (except FASnBr_3_ in which a 5 × 5 × 5 supercell was used instead) and sensible bond distance constraints were applied to ensure the optimized model was chemically sensible. Atomic swap probabilities were set to 85% for Pb and Sn given the alloyed nature of the compounds. The RMC analysis consisted of a minimum of 5 × 10^6^ steps to ensure the optimization was reasonably converged and the partial atomic pair correlations were exported as radial distribution functions.

### 2.11 X-ray photoelectron spectroscopy

Experiments were performed using a Thermo Scientific Nexsa G2 system (monochromated Al K_α_ radiation, ∼1486.6 eV) at a pressure of ∼6.4 × 10^−7^ mBar and with an analysis spot size of 50 µm. Samples were charge compensated with a flood gun. The sample surface was cleaned with 600s of 2000 atom Ar cluster gun with a 150 µm spot size prior to data collection. All peaks were charge-corrected to adventitious carbon at 284.8 eV. Sn 3d spectra were fit with CasaXPS using a Spline Tougaard background. Peak widths were not allowed to be larger than 3.5 eV. All other data were fit with Thermofisher Avantage.

## 3 Results and discussion

### 3.1 Synthetic aspects

Synthetically, the FA_1−*x*_en_*x*_Pb_*y*_Sn_*η*−*y*_Br_3_ hollow perovskite alloys and their derivative parent compounds behave similarly to the recently reported FASnI_3−*x*_Br_*x*_ (*x* = 0–3) series^51^ and we attribute the similarities to a similar competition between the pseudo-0D FASnBr_3_ structure type^51,54^ with the robust 3D FAPbBr_3_ cubic type:^38^ an identical structure to FASnI_3_.^22^ As a result, the Sn fraction in the final product, in our experience, is always lower than what was charged to the reaction.

When considering the parent Pb/Sn alloy compounds (FAPb_1–*y*_Sn_*y*_Br_3_) we note the critical role that the Pb^2+^ concentration plays in obtaining a compound with the desired Pb/Sn fraction. Synthetically, Pb^2+^ was treated similarly to a limiting reagent due to the tendency of Pb-rich phases to more easily precipitate upon cooling. This effect is derived from the considerable difference in solubility of FASnBr_3_ and FAPbBr_3_. Using HBr as the solvent, relatively higher concentrations of FASnBr_3_ can be tolerated without precipitation. For example, to fully dissolve 3 mmol of FASnBr_3_ (a typical amount for an exploratory reaction) at ∼120 °C, only 3–4 mL of solution is needed. Given the same conditions, only half the amount of FAPbBr_3_ is fully dissolved. Therefore, the Pb^2+^ concentration must be carefully controlled. We stress that controlling the loading stoichiometry alone is insufficient and instead the relative concentrations of Pb^2+^ and Sn^2+^ should be considered in the context of the solubility differences.

Now considering the entire range of hollow Pb/Sn alloys, the experimental *χ*_Sn_ is nearly consistent across the various ethylenediammonium substitution ranges for each alloy with minor deviations. Given the consistency, the impacts of en^2+^ incorporation on the structure, optical, and physical properties can be examined directly without considering the effects of variable Pb/Sn ratios. In further discussions of the Pb/Sn alloys, the representative experimentally determined*χ*_Sn_ for the three groups which have approximate values of 0.2, 0.5, and 0.75 corresponding to 0.33, 0.67, and 0.83 *χ*_Sn_ loading respectively.

**Fig. 1:**
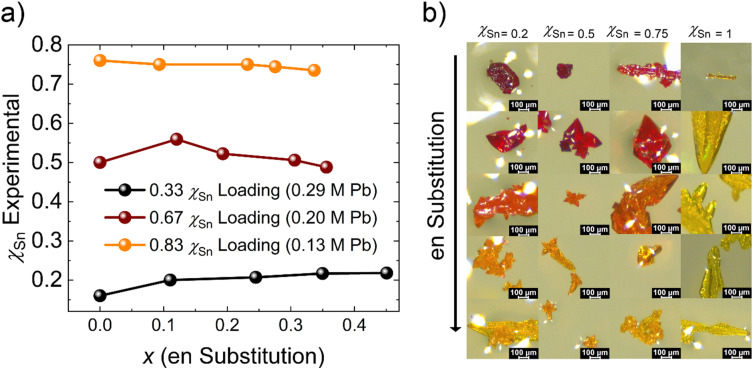
(a) Depicts the mol fraction of Sn (*χ*_Sn_ Experimental), determined by ICP-OES, as a function of ethylenediammonium substitution (*x*) in the FA_1−*x*_en_*x*_Pb_*y*_Sn_*η*−*y*_Br_3_ family. The mol fraction of Sn charged to the reaction solution (*χ*_Sn_ Loading) is also labeled with the corresponding Sn^2+^ reaction concentration in mol L^−1^ determined from the synthetic conditions (see the SI). (b) A selection of crystal images to illustrate typical crystal morphologies and colors. The representative experimental Sn fraction is labeled for each column of crystals.

We also highlight the change in crystal morphology as a result of altering the Pb/Sn ratio and the addition of en^2+^ into the structure. Mixing Pb/Sn causes an elongation of the typical rhombic dodecahedron, becoming more rod-like with increasing Sn concentration. A similar trend was observed in the FASnI_3−*x*_Br_*x*_ (*x* = 0−3) where increasing Br fractions resulted in more rod-like crystals.^51^ The rod can be obtained by extending the cuboctahedra morphology in one direction (Fig. 1b).

Ethylenediammonium loading has a different impact on the crystal morphology which has been documented in our previous research efforts,^39^ but we will summarize it here for clarity. The typical rhombic dodecahedral shape results from the crystal growth occurring along the 〈110〉 direction in the cubic class and the extended rods more typical of the Sn-rich families are an asymmetrical growth along the same direction. The halide and metal are then expected to terminate the crystal. Regardless of the base morphology, the FA_1−*x*_en_*x*_Pb_*η*−*y*_Sn_*y*_Br_3_ hollow perovskites have crystal habits more consistent with regular octahedrons generated by growth along the 〈111〉 body diagonal. Asymmetric growth then leads to crystals of various shapes (Fig. 1b).

Lastly, we briefly highlight the materials' sensitivity to air which can be inspected visually and through PXRD. As can be expected from extrapolating the behavior of FAPbBr_3_ and FASnBr_3_,^38,51^ the alloys have varied susceptibilities to oxidation with dependence on the Sn concentration; Sn-rich samples were more easily oxidized than Pb-rich. The inclusion of ethylenediammonium into the structure improves the air-stability similar to previous reports.^39^ Fig. S20–S27 show the experimental powder diffraction patterns of select compounds after an initial and at least 24 hour period of exposure to air. Furthermore, the improvement in stability can be physically seen by comparing the darkening of FASnBr_3_ crystals (due to SnO formation) after 4 hours of air exposure against FA_0.8_en_0.2_Sn_0.9_Br_3_ (Fig. S28).

### 3.2 Compositional characterization

In our previous works,^38,39^ we thoroughly examined an unusual class of termed “hollow” perovskites in which we determined that ethylenediammonium (en^2+^) can be substituted into the MHP architecture. However, this is not a classical substitution of one cation replacing another. Rather in addition the {*en*} cation also substitutes part of the inorganic sublattice, and mainly the metals. The pattern was determined to be asystematic in nature, and we relied on a compilation of characterization techniques to determine how and what atoms were “removed” from the lattice in favor of en^2+^. *Via* the examination of the ^1^H NMR and experimental density, amongst other techniques, a general formula was devised as the best representation of the presented materials: A_1−*x*_en_*x*_M_1−0.7*x*_X_3−0.4*x*_ where *x* is reported to vary from 0 to 0.44 with a dependence on the general phase space. In this study, we report a compositionally more complex set of hollow perovskites, represented by the general formula FA_1−*x*_en_*x*_Pb_*η*−*y*_Sn_*y*_Br_3_ (*η* = 1–0.5*x*), combining the tuneability exhibited by the existing class of materials with the reported band-bowing effects of Pb/Sn alloying. Full characterization of these materials involved the reexamination of the substitution patterns of en^2+^ to clarify if and how Pb/Sn alloying impacts the existing hollow perovskite paradigm.

#### 3.2.1. Quantitative ^1^H NMR

**Fig. 2:**
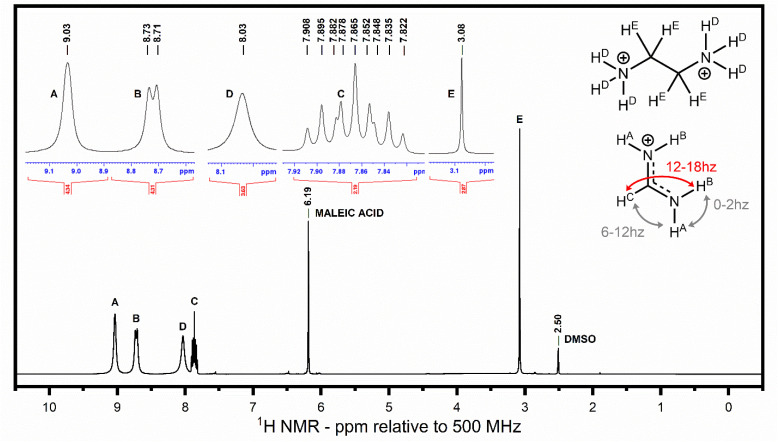
^1^H NMR (500Mhz, DMSO-d_6_ = 2.50) *δ* 9.03 (br s, 2H, A, FA exo NH), 8.72 (br d, *J* = 13.8 Hz, 2H, B, FA endo NH), 8.03 (br s, 3H, D, EN NH_3_), 7.86 (dddd, *J* = 14.9, 14.9, 6.4, 6.4 Hz, 1H, C, FA CH), 3.08 (s, 4H, E, EN (CH_2_)_2_; dddd = doublet of doublets of doublets of doublets).

Formamidinium spectroscopically behaves in a manner similar to amides at low temperature, with rotation about the carbon–nitrogen bond slow enough that the endo and exo hydrogens of each –NH_2_ group become magnetically inequivalent, resulting in two separate downfield peaks. These peaks are susceptible to geminal, *cis*, and *trans* coupling to nearby –N–H and –CH^−^ hydrogens, but peak broadening stifles the observation of the geminal and cis coupling, allowing only high-frequency trans coupling to be observed between –CH^−^ and endo –N–H hydrogens as seen in the broad doublet (13.9 Hz) at 8.72 ppm (Fig. 2). Since endo and exo hydrogens are nonequivalent, the splitting pattern of the –CH^−^ hydrogen is most accurately described as a doublet of doublet of doublet of doublets, which appears as an apparent triplet of triplets due to coupling constant equivalencies of –N–H endo (14.9 Hz) and –N–H exo (6.4 Hz) coupling from both –NH_2_ moieties.

For the purposes of integration calculations, the intensity of the –NH_3_ moiety of ethylene diamine (en) was not used as it showed appreciable levels of signal loss from deuterium exchange. This was observed as a systematic relative decrease in intensity of the –NH_3_ peak relative to the –CH_2_^−^ peak.

The –NH_2_ moieties of formamidinium (FA), which are significantly more basic than ethylene diamine, did not show any signs of deuterium exchange, with the –NH_2_ integration having a consistent ∼2*x* intensity relative to the –CH^−^ peak as expected. At high concentrations of en the –CH^−^ peak of FA experiences an increase in intensity from being adjacent to the broad en –NH_3_ peak. However, the effect is minor enough that the exclusion or inclusion of this peak has very little effect on the calculated en/FA ratio, so the peak was always included for consistency. The overall integrations relative to the internal standard suggest an en^2+^/FA^+^ substitution pattern of 1 to 1, commensurate with previous studies. However, we will note the potential for variance fundamentally rooted in the low mass percentages the organic molecules have to the overall measured mass of each material, meaning a relatively small deviation in the sample mass dissolved for NMR analysis can have a measured impact on the calculated concentrations. The integrated values, relative to the internal standard, and individual NMR spectra are presented in Table S9 and Fig. S1–S5.

#### 3.2.2. X-ray photoelectron spectroscopy

**Fig. 3:**
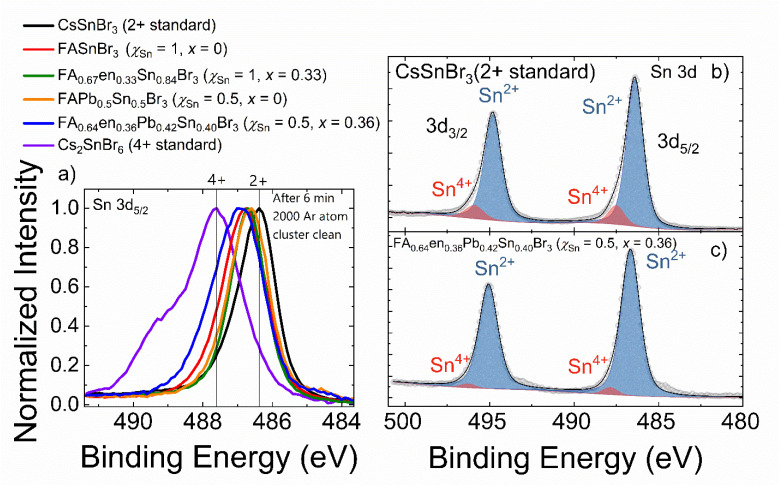
(a) Sn 3d_5/2_ spectrum of 2 standards (CsSnBr_3_ and CsSnBr_6_) and selected samples. The samples include a hMHP (FA_0.67_en_0.33_Sn_0.84_Br_3_ (*χ*_Sn_ = 1, *x* = 0.33)), a mixed Pb/Sn (FAPb_0.5_Sn_0.5_Br_3_ (*χ*_Sn_ = 0.5, *x* = 0)) and both hMHP and Pb/Sn mixed sample (FA_0.64_en_0.36_Pb_0.42_Sn_0.40_Br_3_ (*χ*_Sn_ = 0.5, *x* = 0.36)). All samples have a similar FWHM and peak position as the FASnBr_3_. All the samples are mainly Sn^2+^ with minor Sn^4+^ components. (b) Fitted Sn 3d spectra for the 2+ standard showing a small (11%) Sn^4+^ contribution compared to sample (c) FA_0.64_en_0.36_Pb_0.42_Sn_0.40_Br_3_ (*χ*_Sn_ = 0.5, *x* = 0.36), which showed only 4%.

Given the tendency of Sn^2+^ to undergo oxidation to Sn^4+^ in MHP materials, we examined the oxidation state of Sn *via* the Sn 3d_3/2_ and 3d_5/2_ photoelectron spectra for a subset of samples (FAPb_0.5_Sn_0.5_Br_3_, FA_0.64_en_0.36_Pb_0.42_Sn_0.40_Br_3_, FASnBr_3_, and FA_0.67_en_0.33_Sn_0.84_Br_3_) to eliminate a Sn^4+^ mediated substitution pattern. Due to the small difference in binding energy between the Sn^2+^ and Sn^4+^, as synthesized (see the SI for details) CsSnBr_3_ (Sn^2+^) and Cs_2_SnBr_6_ (Sn^4+^) were use as standards to establish a baseline. CsSnBr_3_ shows a single narrow peak with a full width at half maximum (FWHM) of 1.1 eV, while the spectrum of Cs_2_SnBr_6_ consists of two contributions including from an oxide species shown as a shoulder with higher binding energy (Fig. 3). Nonetheless, a 1.2 eV separation is observed between the standards which is consistent with tin bromides.^55,56^ When substituting Cs^+^ for FA^+^ into the framework there is a shift of + 0.36 eV in the 3*d*_5/2_ binding energy peak. The shift is attributed to the introduced N–H⋯Br interactions that impose larger octahedral tilts, that polarize the cage, reduce the Sn–Br covalency, and result in a higher electrostatic potential at the Sn site.^57^ Although there is a peak shift, there is little change in the FWHM compared to the 2+ standard (1.2 eV *vs.* 1.1 eV), indicative that there is no new species/peak.

To ensure there is no new Sn^4+^ species, the Sn 3d spectrum for all samples was fit considering contributions from both 4+ and 2+ peaks spaced 1.2 eV apart (the 2+/4+ standards spacing). The resultant fits for all measured materials are shown in SI Fig. S29–S34. Including the 4+ peak components improves the overall fit for all samples, as is to be expected when fitting with additional peaks; however, the percent concentration of Sn^4+^ for the majority of samples was determined to be less than the CsSnBr_3_ (11%; Table S11). The only sample with a higher estimated concentration was FAPb_0.5_Sn_0.5_Br_3_ (12%), while the tested hMHP samples had a substantially lower estimated Sn^4+^ concentration (4%) for both FA_0.64_en_0.36_Pb_0.42_Sn_0.40_Br_3_ and FA_0.67_en_0.33_Sn_0.84_Br_3_. These findings simultaneously support the observed increase in air-stability for the synthesized hMHP materials as well as eliminate substitution patterns that are facilitated by Sn^4+^ states.

#### 3.2.3. Structural refinement and en^2+^ substitution pattern

With the collective compositional information gathered from quantitative ^1^H NMR and ICP-OES studies, each crystal structure was refined, including FA^+^ organic vacancies and fixing the Pb^2+^/Sn^2+^ ratio to the experimentally determined values. Initially, the overall M^2+^ occupancy (*η*) was unrestricted in the refinement leading to the incorporation of vacancies. Considering only M^2+^ vacancies, the refined *η* frequently converged near charge neutrality without the inclusion of Br^−^ vacancies or the usage of restraints. The final criterion for selecting the appropriate overall formula for these materials was to restrict it to be charge-neutral and compare the resulting experimental and calculated densities from the refinements. An absolute value for the difference in density (Δ*ρ*) of ≥0.1 g cm^−3^ was chosen to be the cutoff for good agreement between the model and experiment and a 0.1 ≤ Δ*ρ* ≤ 0.15 g cm^−3^ was denoted as moderate agreement. A Δ*ρ* of ≤0.15 was considered poor.

**Fig. 4:**
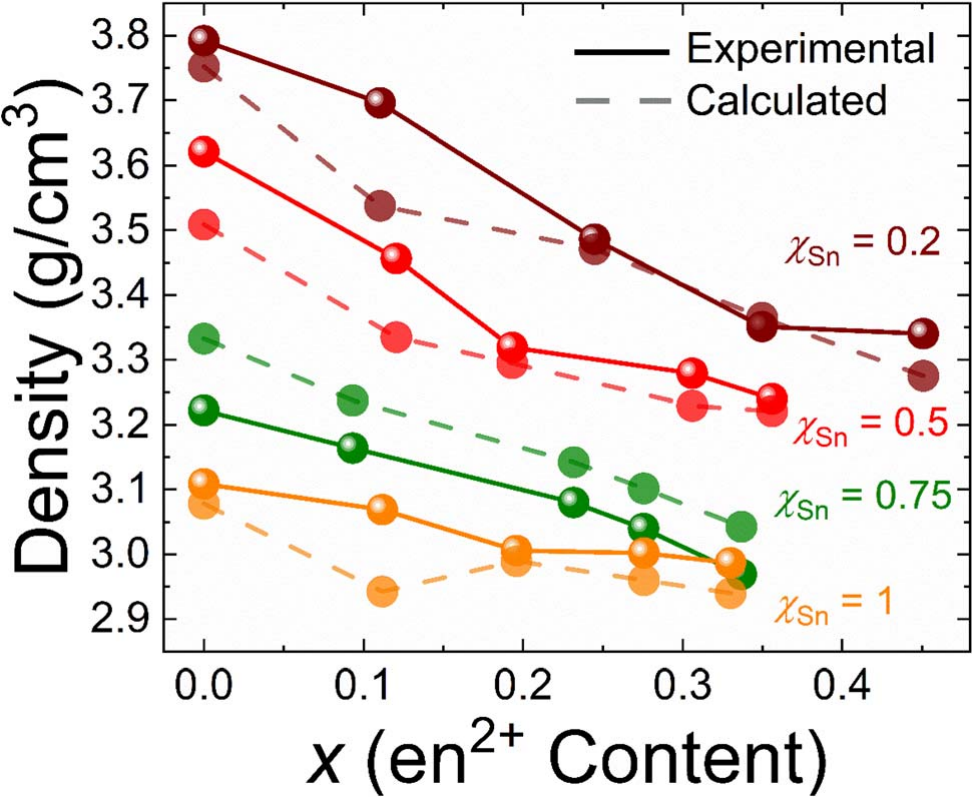
Experimental and calculated density (g cm^−3^) from single crystal refinements for each material plotted as a function of ethylenediammonium (en^2+^) substitution. The experimental and calculated densities are represented by solid and dashed lines, respectively.

In our experience, the calculated density from SXRD refinements, in most cases, was lower than the experimentally determined density. This provided an additional criterion for the elimination of theoretically possible substitution patterns: if the model resulted in a systematically lower density than a previous model, it was rejected. Indeed, this was the case for our previous model (A_1−*x*_en_*x*_M_1−0.7*x*_X_3−0.4*x*_) since the inclusion of Br vacancies resulted in systematically lower theoretical densities. The only substitution pattern that both included en^2+^ substitution for FA^+^ and produced a reasonably accurate density was FA_1−*x*_en_*x*_Pb_*η*−*y*_Sn_*y*_Br_3_ (*η* = 1–0.5*x*). The suggested en^2+^ substitution differs from our previous works, however, this formula is rooted in improved characterization techniques including quantitative compositional analyses. As a result, we suggest that the presented formula more accurately represents the nature of the substitution patterns of en^2+^ in the metal halide perovskite lattice, improving our overall understanding of these exotic materials. The experimental and calculated densities for each material are shown in Fig. 4 as solid and dashed lines respectively.

### 3.3 Structural description

#### 3.3.1. General remarks

**Fig. 5:**
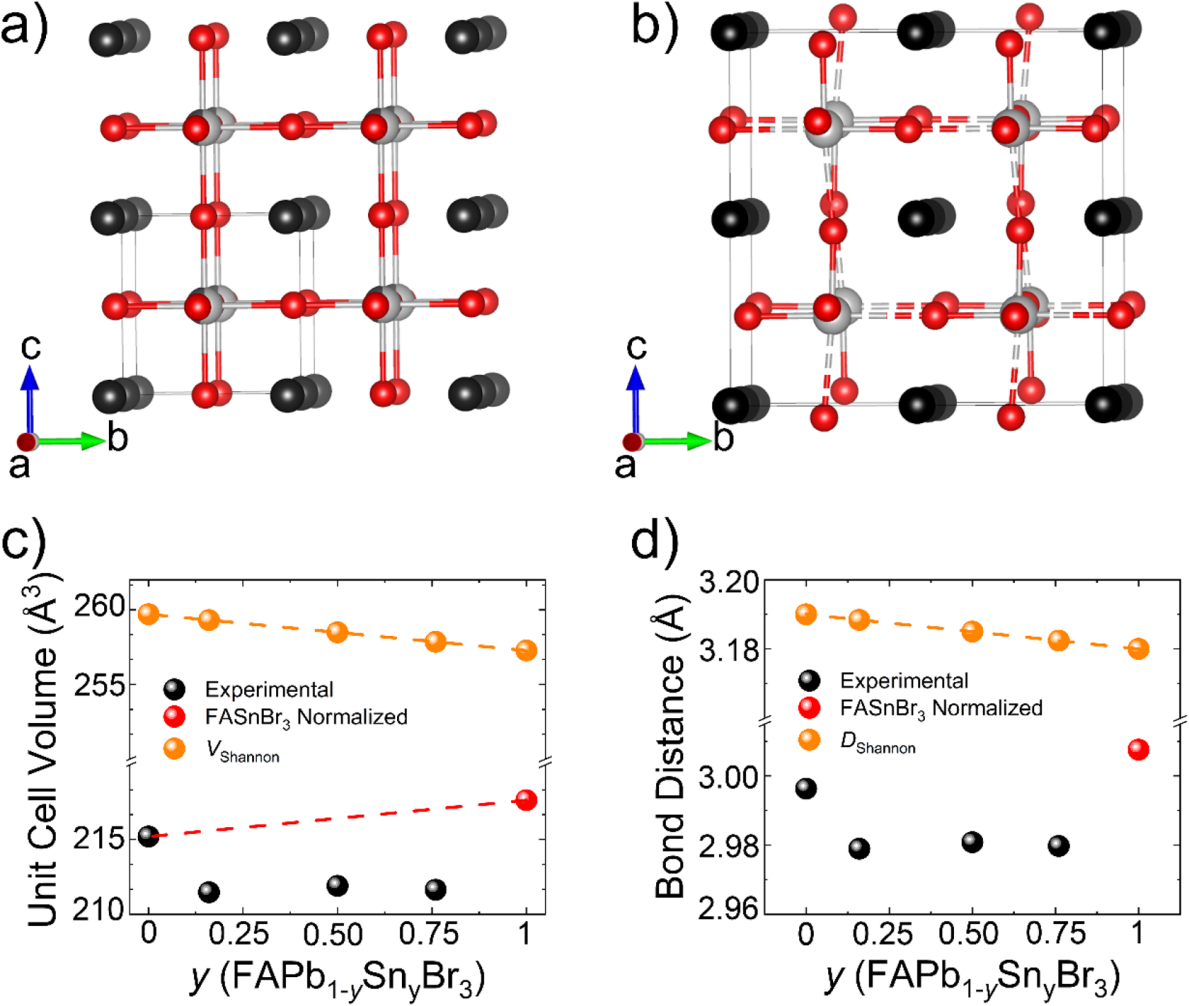
(a) The overall structure for FAPb_0.5_Sn_0.5_Br_3_ and (b) FASnBr_3_ as displayed down the crystallographic *a*-axis, (c) the trend in the experimental unit cell volume compared to the theoretical Shannon volume as a function of Sn content, and (d) the trend in bond distance compared to the predicted Shannon distance as a function of Sn content. The atoms are shown in grey, silver, red, and black for Pb, Sn, Br, and FA^+^ respectively.

All of the materials, with the exception of FASnBr_3_ (Fig. 5b) which has a pseudo-0D type structure with a 2-fold superlattice and *Pa*3̄ space group symmetry,^51,54^ crystallize in a cubic crystal system with space group symmetry *Pm*3̄*m*. The metals (Pb/Sn) were found to reside in the center of an idealized octahedron coordinated to 6 Br atoms. The octahedra are corner-shared with one another in all three crystallographic directions forming the prototypical perovskite framework (SrTiO_3_-type; *a*^0^*a*^0^*a*^0^ Glazer notation)^58–61^ consisting of a 3D cage surrounding the FA^+^ cation (represented by a black sphere in our depiction) which resides at the center to balance the negative charge of the covalent framework (Fig. 5a).

#### 3.3.2. The crystal structure of the pristine FAPb_1−*y*_Sn_*y*_Br_3_ alloys

The pristine parent compounds, represented by the general formula FAPb_1−*y*_Sn_*y*_Br_3_ (*y* = 0–3), structurally deviate from ideality in a variety of ways. Most notable is the bowing seen in the unit cell volume as a function of Sn content (Fig. 5c) where all three of the measured Pb/Sn alloy compounds show volumes that are both roughly consistent with each other (∼211 Å^3^) and significantly lower than what would be expected based on a Vegard's law analysis. Furthermore, the bond distances mirror the trend (Fig. 5d) as expected for an undistorted MHP structure. For the purposes of this comparison, the unit cell volume of FASnBr_3_ was normalized to that of the FAPbBr_3_ type which has been shown in previous research to yield a good approximation for assessing ideality.^51^ Lastly, we highlight that the observation of unit cell contraction (relative to FASnBr_3_) is consistent with measurements on the bulk with clear shifts in peak positions in the powder diffraction patterns.

Due to the distortions in the chemical environment of Sn, the average bond distance presented for FASnBr_3_ was approximated by reducing the normalized cell volume since the cell edge is equivalent to 2*x* the M–Br bond distance in a pristine MHP structure. The observed trends are in contradiction to predictions made by the summation of the relevant ionic radii (*D*_Shannon_), commonly known as the Shannon ionic radii.^62,63^ The observed gap between the predicted and experimental distances, and Shannon volume (*V*_Shannon_) by extension, can be reconciled with the covalent nature of the M–Br bond. As such the summation of ionic radii overestimates the bond length for compounds that are more covalent in nature. However, the decrease in predicted bond length as a function of Sn concentration highlights the level of complexity and competing chemical influences within this phase space. As shown previously in the literature, the Sn in FASnBr_3_ is highly off centered at room temperature due to the 5s^2^ lone pair of electrons competing for its own space, resulting in a normalized unit cell volume that is larger than predicted for a pristine lattice. However, it is unlikely that the same influences are responsible for the observed bowing here. If off-centering were the dominant feature, an upward trend with Sn concentration would be expected (Fig. 5c; red dashed line). Instead, neither analysis method (Vegard's Law and *V*_Shannon_) represents what is experimentally observed, which is also inconsistent with the analogous FAPb_1−*x*_Sn_*x*_I_3_ phase space.^64^ Clearly, the average structure alone is insufficient to understand the entirety of these phases. As such we examined the local structure behavior by measuring the total scattering pair distribution function (PDF) as presented later in this work.

#### 3.3.3. FA_1−*x*_en_*x*_Pb_*η*−*y*_Sn_*y*_Br_3_ hollow perovskite average structure description

**Fig. 6:**
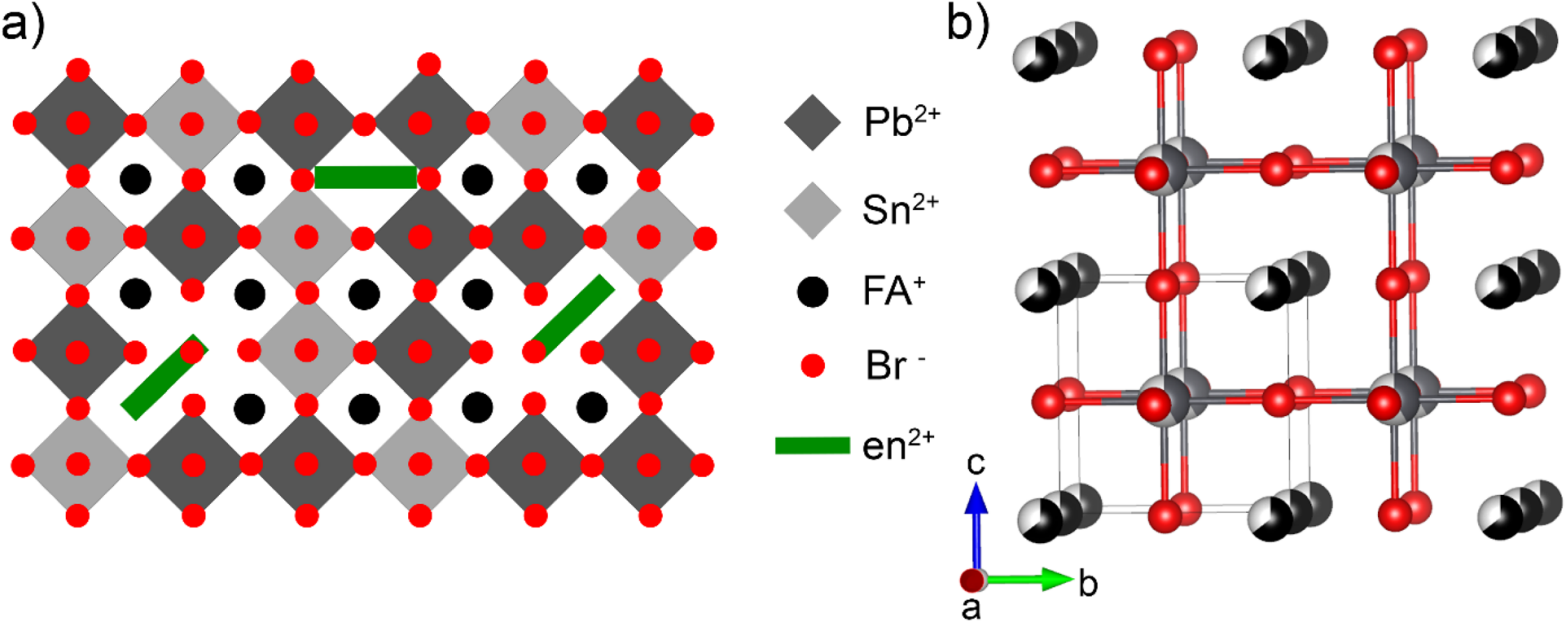
(a) A depiction of the ethylenediammonium substitutions into the metal halide perovskite lattice and (b) a representative average crystal structure for the FA_1−*x*_en_*x*_Pb_*η*−*y*_Sn_*y*_Br_3_ family of hollow perovskites. The atoms are shown in grey, silver, red, black, and white for Pb, Sn, Br, FA^+^, and vacancies respectively.

All of the termed “hollow” perovskite alloys crystallize in a cubic crystal system at room temperature with *Pm*3̄*m* space group symmetry. The average structure is best described as a “defective” MHP architecture with ethylenediammonium introducing vacancies on the M and FA^+^ site since the substitutions are random. As such, by increasing the en^2+^ concentration, it can be considered that the lattice becomes more “defective” as the 3D framework is more frequently disrupted by the presence of the dication. A pictorial representation of the hollow perovskite structure is presented in Fig. 6 as a visualization aid. An example hollow perovskite alloy structure is depicted in Fig. 6a with the FA^+^ molecule represented as a black sphere for simplicity.

The hollow perovskites also exhibit lattice expansion as a function of the en^2+^ dictation substitution with little exception based on the refined unit cell volumes from room temperature single crystal X-ray diffraction experiments (Fig. S6). Room temperature X-ray powder diffraction experiments confirm the expansion through the observation of shifted diffraction peaks (Fig. S14–S17). The Pb/Sn hollow alloys exhibit a composition-dependent rate of expansion with en^2+^ substitution (*x*). The calculated slope of the *χ*_Sn_ = 0.2 regression line (20 ± 5 Å^3^/*x*) is consistent with the full Pb family experimental data (21 ± 3 Å^3^/*x*).^38^ However, as the Sn fraction increases, the lattice expansion rate follows with the *χ*_Sn_ = 0.5 and 0.75 data sets having values of 22 ± 5 Å^3^/*x* and 31 ± 5 Å^3^/*x* respectively. Curiously, this rate shows a drastic decrease for the full Sn series (7.5 ± 0.9 Å^3^/*x*), possibly rooted in the enlarged normalized volume of FASnBr_3_ relative to FAPbBr_3_, *vide supra*. Adding to structural complexity, FA_0.66_en_0.34_Pb_0.22_Sn_0.61_Br_3_ has the largest cell volume of all of the hollow bromide perovskites (223.0 Å^3^) despite not having the highest reported en^2+^ substitution (34% compared to the 42%^38^) and the second lowest parent volume: FAPb_0.24_Sn_0.76_Br_3_ has a cell volume of 211.6 Å^3^ which is narrowly larger than 211.5 Å^3^ for FAPb_0.84_Sn_0.16_Br_3_. Clearly, the structural dynamics of the FA_1−*x*_en_*x*_Pb_*η*−*y*_Sn_*y*_Br_3_ (*η* = 1–0.5*x*) hollow perovskite family is intricate, requiring magnified efforts to fully grasp the underlying chemistry giving rise to the phenomena described here.

The peculiarities of this phase space lie not only in the Pb/Sn alloys but also in the full Sn phases as well. The hollow Sn bromide perovskites, described by the formula FA_1−*x*_en_*x*_Sn_*η*_Br_3_ (*η* = 1–0.5*x*), indicate en^2+^ substitution has an effect of regulating the Sn off-centering (5*s*^2^ loan pair expression) present in the room temperature average structure of FASnBr_3_: a compound which is known to crystallize in a superlattice with lower symmetry (Pa3̄) and more discrete [SnBr_3_]^−^ pyramidal units.^51,54^ The substitution of ∼12% of en^2+^ into the lattice suppresses the superlattice, crystallizing in *Pm*3̄*m* with a more robust framework of corner-shared SnBr_6_ octahedra as expected for a typical MHP. The 3D architecture is then adopted for the remainder of the analyzed en^2+^ concentrations analyzed in this work, a similar phase transition suppression is experienced for the FAPbI_3_ and MAPbI_3_ systems where ethylenediammonium substitution has the effect of straightening the critical Pb–X–Pb octahedral tilting angles to the idealized 180° with a corresponding shift in the space group symmetry towards the classical P*m*3̄*m* symmetry with little exception.^39^ As such, the tendency of hollow perovskites towards adopting an idealized architecture is well documented and continues with this assessment. To further gain a more detailed understanding of the chemical influences on the structure of these materials, the local structure of each material was analyzed through total scattering pair distribution function (PDF) measurements.

#### 3.3.4. Total scattering pair distribution function (PDF)

Total scattering PDF measurements utilize high-resolution powder diffraction experiments to extract the real space (Å) distribution of distance vectors from an arbitrary center, also known as the PDF curve. As such, examining the curve (*G*(*r*)) can yield information regarding the ordering of atoms on a localized scale, including the distribution of bond distances for a single unit cell without the qualifying condition of crystallinity. Perturbations in the *G*(*r*) away from expectations based on the average crystal structure then yield insight into localized distortions that cannot be easily determined through traditional crystallographic experiments.

For a typical hybrid metal halide perovskite containing Br as the halide anion and Pb/Sn as the group 14 metal, the low *r* region of the PDF curve should be dominated by the M–Br bond followed by the nearest neighbor Br–Br distances. FAPbBr_3_, for example, has an average Pb–Br bond distance of 3 Å followed by 4.2 Å distance for Br–Br nearest neighbors, as determined by the room temperature structure. FASnBr_3_ on the other hand, displays a distorted lattice with three short Sn–Br bonds (2.7 Å) and three long distances (3.3 Å), creating a wider range of nearest neighbor Br–Br distances giving rise to a much broader PDF curve in the 3–5 Å range. In this regard, the behavior of the local structure is paramount to understanding the optoelectronic properties of the material as the lone pair expression exhibited in FASnBr_3_ has been tied to drastic quenching of the photoluminescent properties of MHPs.^51^

Hollowing of a perovskite lattice also has signatures throughout the PDF curve, though more subtle, characterized primarily by a distinct decrease in the density of the distance vectors based on the decrease in the concentration of atoms with high scattering power (Pb and Sn). Tailing or shouldering of the primary M–X distance vector may also be present in correspondence with stretching of the bond in agreement with shifts in the average unit cell parameters extracted from single crystal and powder diffraction experiments. For the FA_1−*x*_en_*x*_Pb_*η*−*y*_Sn_*y*_Br_3_ family of compounds, a convolution of these two local structure dynamics is seen in the experimental PDF curve.

**Fig. 7:**
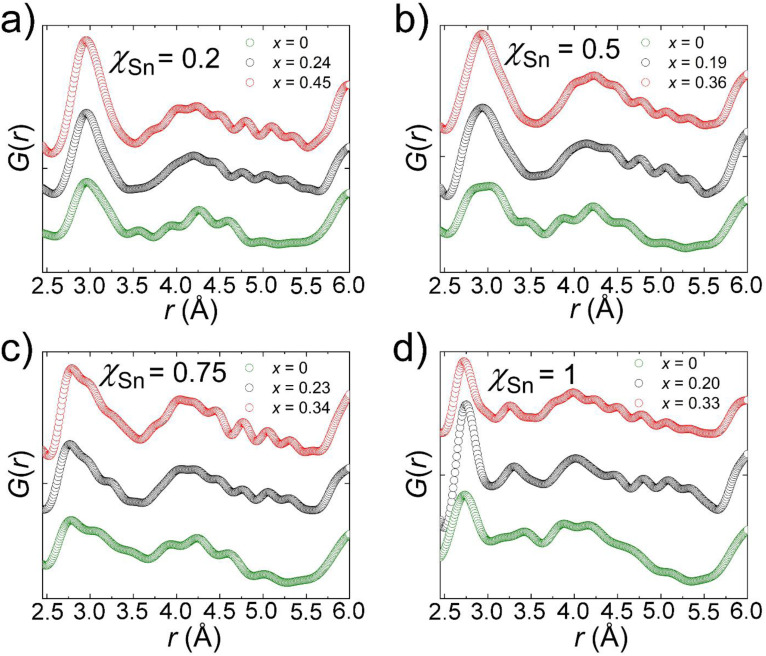
Experimental pair distribution function (PDF) curves for representative compounds in the FA_1−*x*_en_*x*_Pb_*η*−*y*_Sn_*y*_Br_3_ family of perovskites in four panels (a–d) depending on the experimentally determined Sn fraction (*χ*_Sn_) and ethylenediammonium fraction (*x*). Here the PDF of FASnBr_3_ is plotted from our previous works for comparison.^51^

The experimental PDF curves for representative samples with varying Sn and en^2+^ fractions are shown in Fig. 7, specifically highlighting the region indicative of the local structure of the material within considering the distribution of interatomic distance correlations present within a single unit cell (2.5–6 Å). To deconvolute the local structure implications of the Pb/Sn mixing and ethylenediammonium substitution, we first analyze the *G*(*r*) by comparing the PDF of the materials with similar en^2+^ substitutions but varying *χ*_Sn_ followed by an analogous analysis where the comparison criteria are inverted.

#### 3.3.5. PDF analysis of the FAPb_1–*y*_Sn_*y*_Br_3_ alloys

As the Sn concentration increases, there is a notable broadening/shouldering of the distance vector correlation for the M–Br bond as a result of a convolution of distance vectors with different peak maxima. This is particularly evident in the curves for the *χ*_Sn_ = 0.2 and *χ*_Sn_ = 0.5 where the former has a maximum at ∼2.97 Å with shouldering towards higher *r* and the latter has two maxima of nearly equivalent density centered at ∼2.88 and ∼3.03 Å. The distribution distances can be reconciled with the crystal structures for each compound since FAPb_0.84_Sn_0.16_Br_3_ and FAPb_0.5_Sn_0.5_Br_3_ have average distances of 2.9788(7) and 2.9808(9) Å. The two peaks present in the PDF curve of FAPb_0.5_Sn_0.5_Br_3_ show increased separation best described by the local structure exhibiting M^2+^ off-centering for FAPb_0.24_Sn_0.76_Br_3_. The rationale is confirmed by the presence of a 2.77 Å distance vector consistent with the short bond distance in the [SnBr_3_]^−^ motif in FASnBr_3_ (2.716(1) Å) and the 2.74 Å vector in the corresponding PDF curve. The second most prominent peak in the region that is contributed to M–Br bonds is centered at 3.09 Å more consistent with an elongated Pb–Br bond in the 3D FAPbBr_3_ structure (2.9963(2) Å).

Curiously, the experimental PDF curves of all three mixed Pb/Sn compounds have a peak with a relatively low density centered at 3.47 Å that we attribute a long Sn–Br distance similar to that reported for the pseudo 0D FASnBr_3_. The Pb-rich samples, therefore, exhibit similar distortions but to a lesser degree. The competitive nature was previously identified through computational analyses but were not experimentally confirmed until now.^65^ Furthermore, the initially puzzling lack of photoluminescence for the FAPb_1−*y*_Sn_*y*_Br_3_ family of perovskites is then reconciled with the presence of these lattice distortions on a local scale since the property is both sensitive to defect state concentration, and the disruption of the MHP inorganic framework even when not ordered in the long range. Importantly, these lattice distortions are fundamentally different from the rocking (tilting) of the octahedra as the M–X–M bond rapidly bends characterized by enlarged atomic displacement parameters (ADPs) perpendicular to the M–X bond direction. These influences are what give rise to phase transitions at lower temperatures where the octahedral tilting angles average to less than 180°.

Examination of the remainder of the low *r* region of the PDF curves shows a broad distribution of distance vectors, which can be attributed to a wide range of nearest neighbor Br–Br distances with variance depending on both the M^2+^ off-centering behavior and the identity of the metal. The *χ*_Sn_ = 0.2 curve, for instance, shows three distinct overlapping distance distributions at ∼3.95, 4.26, and 4.60 Å. The middle of the three is consistent with the average nearest neighbor Br–Br distance in the single crystal structure (4.213(1) Å), while the shorter 3.95 Å vector in the curve is in line with the shortest Br–Br distance in a [Pb/SnBr_3_]^−^ pyramidal unit if the behavior is extrapolated from the known structure of FASnBr_3_. The origins of the longest of the three are less easily deciphered, but its presence persists across all of Pb/Sn alloys, indicating it is a feature of the structure that is not dependent on the composition of the metal site.

#### 3.3.6. PDF analysis of the FA_1−*x*_en_*x*_Pb_η−*y*_Sn_*y*_Br_3_ hollow perovskite alloys

The examination of the local structure of the en^2+^ hollow FA–Pb/Sn–Br phase space loosely confirms the trend shown in the average structures of the *χ*_Sn_ = 1 series in that on a local level, the M^2+^ off-centering is less pronounced on a local scale as the en^2+^ concentration is steadily increased. The best example of this phenomenon is displayed by the trend in the shape of the low *r* region distance distributions for the *χ*_Sn_ = 0.5 series of compounds (Fig. 7b). The distances representative of the Pb/Sn–Br bonds in the material coalesce from a distinct “doublet” for *x* = 0, to a sharper “singlet” with little evidence of shouldering trending towards higher *r* at *x* = 0.36. Furthermore, the distance vector attributed to an elongated M–Br bond present in a pseudo 0D [MBr_3_]^−^ pyramidal arrangement dissipates by an en^2+^ concentration of *x* = 0.19. Lastly, the region of the curve attributed to nearest neighbor Br–Br distances in the perovskite architecture (3.75–4.75 Å) also trends towards the merging of three distinct distance distributions present in the *x* = 0 curve towards one in the *x* = 0.36 experimental PDF which is centered at ∼4.24 Å: a value consistent with the Br–Br nearest neighbor distances present in the robust, 3D average crystal structure for the same phase. The remainder of the Pb/Sn hollow en^2+^ alloys tested exhibit similar behavior albeit with the amount of observed off-centering regulation increasing with Pb content. The local structure of the full Sn FA_1−*x*_en_*x*_Sn_*η*_Br_3_ is, therefore, relatively unchanged, in comparison, with respect to en^2+^ concentration. Lastly, the curves do not display much shifting in the position of the distance vector distributions towards higher *r* and instead remain relatively consistent.

### 3.4 Reverse Monte Carlo (RMC) analysis of atomic pair distribution function data

Given the various chemical influences and lattice distortions displayed by the compounds on the local scale, the experimental *G*(*r*) was fitted through Reverse Monte Carlo simulations, as implemented through fullrmc,^52,53^ fitting 3 ranges of *r* considering a lower limit of 2.5 Å and the upper limits of 6, 12, and 20 Å. The selected ranges allow for an analysis of how the localized lattice distortions are averaged as longer ranges of ordering are considered. Given the disordered/alloyed nature of the M^2+^ site, an 85% swap probability was implemented. The following analysis follows a similar approach to that used to describe the low *r* region of the experimental PDF curves (*vide supra*) in that first the impacts of Sn substitution are examined without considering the addition of en^2+^. Then the ethylenediammonium substitution is considered to more concretely analyze its impacts on the local dynamics. The usage of three different ranges for fitting the experimental PDF curve will also give insight to how the atoms order at longer path lengths throughout the analysis.

#### 3.4.1. Reverse Monte Carlo (RMC) simulation analysis on the parent FAPb_1−*y*_Sn_*y*_Br_3_ compounds

**Fig. 8:**
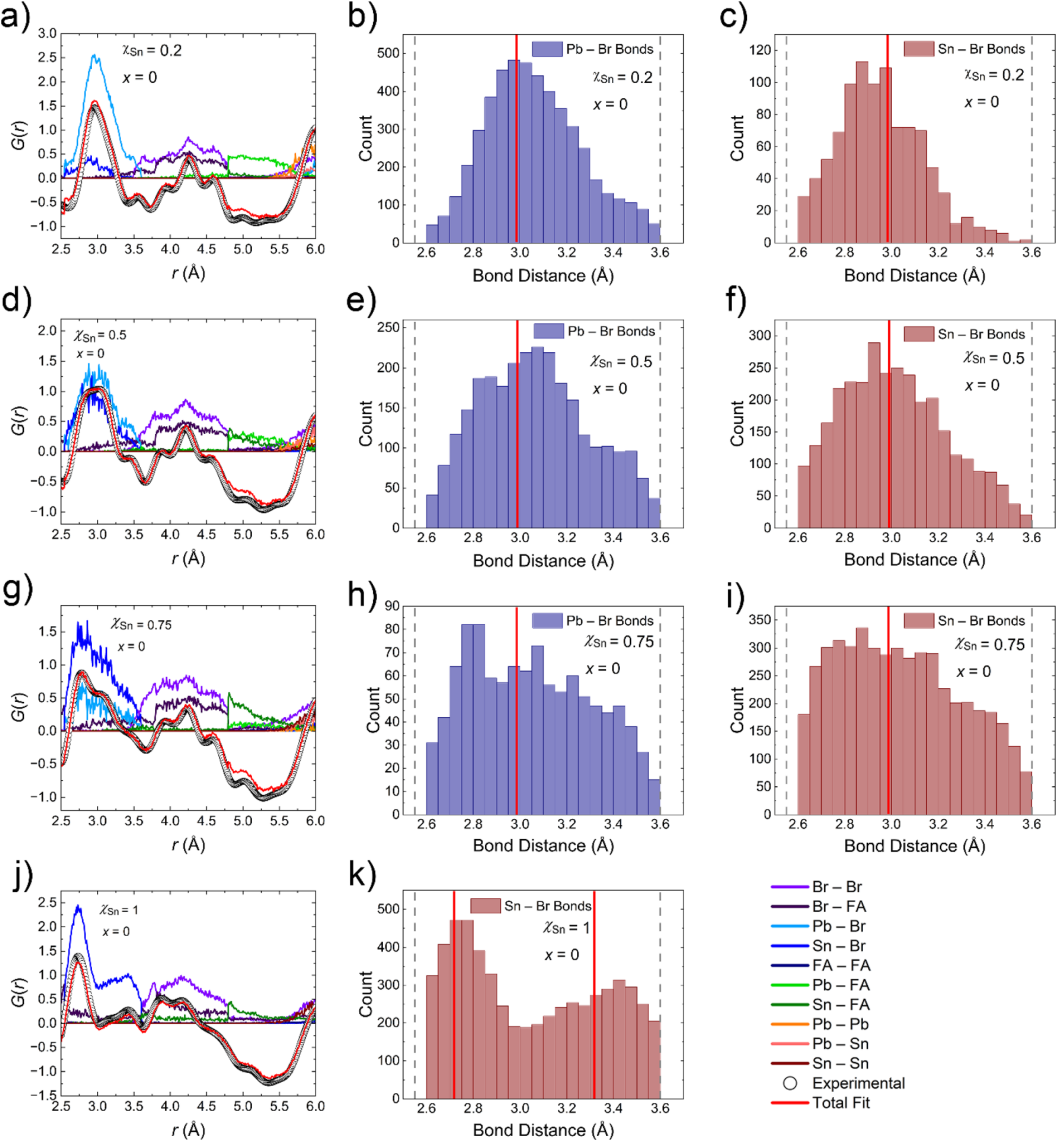
Results from Reverse Monte Carlo fitting of the low *r* region (2.5–6 Å) of the experimental PDF curves for the FAPb_1–*y*_Sn_*y*_Br_3_ (*y* = 0.2, 0.5, 0.75, 1) compounds (a, d, g and j) and histograms of the Pb–Br (b, e and h) and Sn–Br (c, f, i and k) bond distances extracted from the resulting model. The average bond distance(s) determined from single crystal X-ray diffraction are displayed as red line(s) on the histograms.

The simulations confirm the assignments of the PDF peaks in the low *r* region of the curve as discussed in the more qualitative description. Most notably, the pair correlation that occurs for *χ*_Sn_ = 0.2, 0.5, 0.75 at ∼3.5 Å is indeed at least partially contributed to M–X bonds, which is a strong indication for the presence of off-centering on the M^2+^ site. The region of the curve contributed to the M–Br bond, specifically for *χ*_Sn_ = 0.75, is also a strong indication given the broad distribution and the presence of pair correlations around 2.7 Å; more in line with the distorted FASnBr_3_ average structure than FAPbBr_3_. Based on the distribution of bond distances from the 10 × 10 × 10 optimized supercell models (Fig. 8), the Sn–Br distance distributions trend towards shorter lengths than the Pb–Br bonds when compared to the average bond distance obtained from each compound's crystal structure. This is readily apparent for the *χ*_Sn_ = 0.2 and 0.5 samples where the maxima in the distributions are shifted with respect to the maxima in the Pb–Br bonds. The relative shift in the Sn–Br bonds towards shorter distances eludes to another characteristic of the models which show a gradual broadening of distances for all M–X bonds such that the models of the local structure for FAPb_0.25_Sn_0.75_Br_3_ have no distinct maxima in the M–X bond distances. Furthermore, the number of bond lengths less than the average from the single crystal refinement increase with Sn concentration. We attribute this phenomenon to the increase in off-centering with increasing Sn fraction until *χ*_Sn_ = 1 which show a splitting of the Sn–Br distances in accordance with its distorted average structure.^51,54^ We note here that previous modeling of the PDF curve of FASnBr_3_ suggests a trigonal planar-like geometry for the local structure;^66^ however, we dismiss this type of model as a possibility based on the known off-centering distortions at room temperature (space group *Pa*3̄ instead of *Pm*3̄*m* giving rise to a pyramidal-like [SnBr_3_] coordination) as well as reports of analogous [MX_3_]^−^ pyramidal units present in FASnI_0.1_Br_2.9_ (ref. 51) and Ge-based perovskites: CsGeI_3_,^67^ MAGeI_3,_^68,69^ FAGeI_3_,^69^ CsGeBr_3_,^67,70^ RbGeBr_3_,^71^ MAGeBr_3,_^70^ FAGeBr_3_,^70^ and CsGeCl_3_.^67^

The simulations also more concretely pinpoint the origins of peaks in the in the range of *r* ≈ 3.75–5 Å as being from both Br–Br correlations for all of the phases and small contributions from FA^+^–Br distances, although the contributions may be over-approximated given the disordered nature of the organic cation combined with its weak X-ray scattering power. The lack of one centralized Br–Br pair correlation centered around the average Br–Br distance once more is evidence of localized distortions that are not captured in the average crystal structure.

When the range of the PDF curve considered in the model is increased (2.5–12 or 20 Å) there is little change in the bond distance distributions obtained from the simulations (Fig. S7–S9). The persistence of the distributions suggests the level of lone-pair expression is still significant even as longer pair correlations are considered up to 20 Å. Note here that we do not present a variable *r* fitting for FASnBr_3_ since the local distortions persist in the average structure.

#### 3.4.2. Reverse Monte Carlo (RMC) simulation analysis on the hollow FA_1−*x*_en_*x*_Pb_*η*−*y*_Sn_*y*_Br_3_ perovskites

**Fig. 9:**
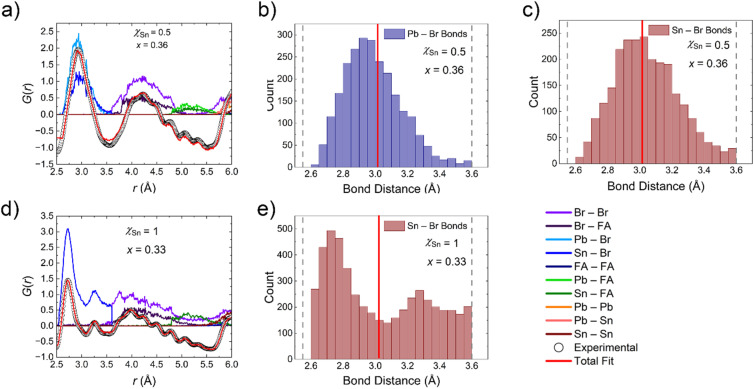
Results from Reverse Monte Carlo fitting of the low *r* region (2.5–6 Å) of the experimental PDF curve for FA_0.64_en_0.36_Pb_0.42_Sn_0.40_Br_3_ (a) and histogram of the Pb–Br (b) and Sn–Br (c) bond distances extracted from the resulting model as well as the corresponding plots for FA_0.67_en_0.33_Sn_0.84_Br_3_ (d and e). The average bond distance determined from single crystal X-ray diffraction are displayed as a red lines on the histograms.

As noted previously, we suggest the primary effect on the local structure of the phases discussed here is the apparent reduction of the local off-centering of the Pb/Sn atoms. This phenomenon is confirmed by the RMC fitting of the of the low *r* region (2.5–6 Å) of the PDF curves for the hollow perovskite compounds with the highest en^2+^ fractions. The trend is most easily identified considering the *χ*_Sn_ = 0.5 series were the M–X bond distances extracted from the 10 × 10 × 10 RMC supercell model are wide ranging given the parameters of the simulation (broad distribution) for *x* = 0 (Fig. 8e and f), but are more concentrated (narrow distribution) towards the measured average bond distance for *x* = 0.36 compound (Fig. 9a–c). The attribution of the broad distribution present for *x* = 0 to local off-centering means by extension that the *x* = 0.36 compound is observably less distorted locally. These findings highlight both the importance and interplay of the local structure components in MHP chemistry, since the desirable optoelectronic properties are heavily influenced by the local coordination environment of atoms, including dynamic features. In this case, the addition of en^2+^ to form a hollow perovskite could then be considered a methodology for regulation of the off-centering behavior known to cause degradation in the optoelectronic properties in MHPs while simultaneously inducing blueshifting of the band gap.

There is, however, an apparent limitation to the regulation capabilities of en^2+^ substitution as the bond distances extracted from the supercell model of FA_0.67_en_0.33_Sn_0.84_Br_3_ are consistent with the distributions obtained from FASnBr_3_, despite the average structure of FA_0.67_en_0.33_Sn_0.84_Br_3_ having a pristine perovskite lattice (Fig. 9d and e). Clearly Sn-rich phases more easily “resist” the regulating effects of ethylenediammonium substitution. Similar to the non-hollow counterparts, analysis of the fitting of the PDF curves considering different ranges of pair correlations (2.5–6, 12, and 20 Å) reveal little deviation in the bond distance histograms (Fig. S10–S13) with the implications being the same as discussed previously.

### 3.5 FA_1−*x*_en_*x*_Pb_*η*−*y*_Sn_*y*_Br_3_ optical property characterization

To understand the trends in the optical band gap for these materials, diffuse reflectance UV-vis spectra were collected on the solid. Sets of spectra, separated by the experimental Sn mol fraction are shown in four panels in Fig. S13. The band gaps were estimated through linear extrapolation of the absorption edge with the observed baseline.

**Fig. 10:**
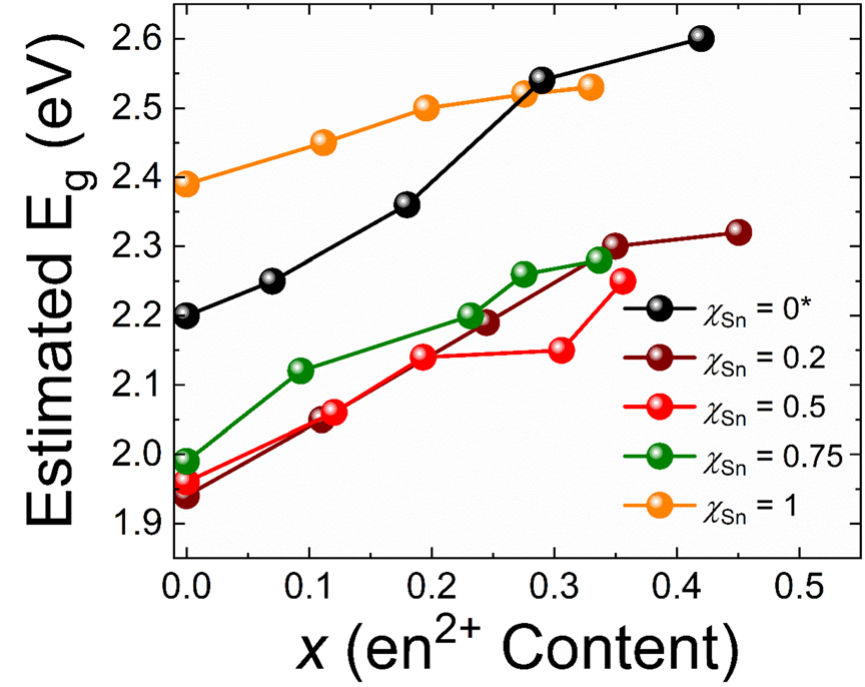
Depicts the trend in estimated *E*_g_ (eV) as a function of the experimentally determined ethylenediammonium content. *The *χ*_Sn_ = 0 line is plotted based on data from our previous works.^38^

The Pb/Sn alloys have narrower bandgaps than either FAPbBr_3_ or FASnBr_3_ exhibiting anomalous band-bowing effects as expected based on the reported iodide counterparts (Fig. 10).^10,26,64^ Here, the widely accepted rationale is rooted in the band edge positions of the pure Pb and Sn counterparts, where the Pb/Sn alloy maintains the valence band maximum because of the contribution of the antibonding states of the 5s^2^ lone pair of electrons of the pure Sn phase and the conduction band minimum of the Pb phase, resulting in an overall narrower gap.^72^ These effects are also extended to the hollow FA_1−*x*_en_*x*_Pb_*η*−*y*_Sn_*y*_Br_3_ family which similarly exhibit narrower gaps. Interestingly, the band-bowing effects observed in the bandgaps can be combined with the blueshifting resulting from en^2+^ incorporation^38,39^ creating a wide range of materials with varying compositions and optical gaps. As a result of both types of substitutions, *E*_g_ values between ∼1.9–2.6 eV, making the phase space highly versatile. Each set of compounds displays a linear trend with regard to en^2+^ substitution, affording predictability in the band gap values.

From an optoelectronic property perspective, the FA_1−*x*_en_*x*_Pb_*η*−*y*_Sn_*y*_Br_3_ family of perovskites represents a departure from the typical paradigm since each compound exhibit little to no photoluminescence (PL). However, the lack of PL can be reconciled with existing literature. The local dynamic M^2+^ off-centering identified in the experimental PDF curves has been reported to be responsible for the extinguishing of the PL in the similar FASnI_3−*x*_Br_*x*_ (*x* = 0–3) phase space.^51^ This is in addition to the reporting of the non-luminescent MAGeI_3_ and FAGeI_3_ phases, which show similar distortions in the average crystal structures.^68,69^ Furthermore, the disruption of the lattice caused by en^2+^ addition has been shown to have a stronger effect in quenching the PL than any enhancement of PL from alleviation of s^2^ lone pair expression^42^ Therefore, when considering all of these factors, the extinguishing of PL in the materials presented in this study is not surprising, but rather an anticipated consequence of the local structure dynamics.

## Conclusions

Optical tuneability is one of the fundamental underpinnings driving extensive scientific research aimed at commercializing metal halide perovskites as active materials in optoelectronic devices. However, the requirements of the architecture place strict limitations on the possible compositions, especially if only the environmentally non-toxic phases are considered. In our most recent work, we demonstrate the optical tuneability offered by hollow metal halide perovskites by extending our original works to the mixed metal FA_1−*x*_en_*x*_Pb_*η*−*y*_Sn_*y*_Br_3_ hollow perovskites. These compounds combine the anomalous reds-shifting effects of Pb/Sn mixing with the progressive shifting of incorporating ethylenediammonium in increasing concentrations to produce a family of compounds with optical gaps ranging from 1.9 to 2.6 eV. We precisely define the substitution pattern of en^2+^ into the perovskite lattice by rigorously combining compositional characterization techniques with structural determination methods. We also analyze the impacts of Sn and en^2+^ substitution on local metal off-centering distortions through comparison of the average structure and local structure determined through single crystal X-ray diffraction and pair distribution function analyses respectively. We find that mixing Pb/Sn leads to increased off-centering while the inclusion of en^2+^ regulates the distortions to some degree while also generating massive amounts of metal vacancies to accommodate the larger cation. Our findings suggest that organic substitutions into the perovskite lattice can be a methodology for tuning not only optical properties in metal halide perovskites but also structural distortions.

## Author contributions

Adam Balvanz: conceptualization, synthesis, crystal structure analysis, atomic pair distribution function data collection and analysis, gas pycnometry data collection and analysis; Anastasia Pournara: elemental analysis data collection and analysis through ICP-OES; Robert P. Reynolds: quantitative NMR data collection and analysis; Patricia E. Meza: XPS data collection and analysis; Christos D. Malliakas: atomic pair distribution function data analysis; Jared D. Fletcher: photoluminescence data collection and analysis; Ram Seshadri: editorial contributions; Vinayak P. Dravid: XPS data analysis and editorial contributions; Mercouri G. Kanatzidis: conceptualization and editorial contributions.

## Conflicts of interest

The authors declare no competing interests.

## Supplementary Material

SC-OLF-D5SC01841B-s001

SC-OLF-D5SC01841B-s002

## Data Availability

CCDC 2426344–2426362 contain the supplementary crystallographic data for this paper.^73*a*–*s*^ Supplementary information: additional materials and methods, crystallographic/refinement information, quantitative NMR tabulations, ICP-OES tabulations, XPS calculations, ^1^H NMR spectra, unit cell plots, results from reverse Monte Carlo analyses, experimental diffuse reflectance spectra, experimental powder diffraction patterns, sample images, and XPS spectra/fittings. See DOI: https://doi.org/10.1039/d5sc01841b.
