# Supplementary material for: Tuning optical properties and local lone-pair off-centering in “hollow” FA1−x{en}xPbη−ySnyBr3 perovskites
Source: Chem Sci. 2025 Oct 31;17(1):526–43. doi: 10.1039/d5sc01841b (PMC12624409; doi:10.1039/d5sc01841b)
Supplement: SC-017-D5SC01841B-s001 [file SC-017-D5SC01841B-s001.pdf]

Supplementary Information (SI) for Chemical Science.
This journal is © The Royal Society of Chemistry 2025

Supporting Information

Tuning Optical Properties and Local Lone–Pair Off-Centering in "Hollow" $FA_{1-x}\{en\}_xPb_{\eta-y}Sn_yBr_3$ Perovskites

Adam Balvanz,[1] Anastasia Pournara,[1] Robert P. Reynolds,[1] Patricia E. Meza,[2] Christos D. Malliakas,[1] Jared D. Fletcher,[1] Ram Seshadri,[3] Vinayak P. Dravid,[2] and Mercouri G. Kanatzidis[1,4,*]

[1]Northwestern University Department of Chemistry, Evanston, IL 60208 USA

[2]Northwestern University Department of Materials Science and Engineering, Evanston IL, 20208 USA

[3]Materials Department and Materials Research Laboratory, University of California, Santa Barbara, CA 93106, USA

[4]Materials Science Division, Argonne National Laboratory, Lemont, IL 60439 USA

[*]Correspondence: m-kanatzidis@northwestern.edu

Table of Contents:

Additional Materials and Methods: (pg. S2-S10)

Crystallographic and Refinement Tables (S1 – S8): (pg. S11-S19)

Table S9: Quantitative ^{1}H NMR Analysis: (pg. S20)

Table S10: Quantitative ICP-OES Compositional Analysis. (pg. S21)

Table S11: Calculated Sn^{2+} and Sn^{4+} Concentrations from X-ray Photoelectron Spectroscopy (pg. S21)

Figure S1: ^{1}H NMR Spectra in the 8.15 to 7.75 ppm Range: (pg. S22)

Figures S2-S5: Full ^{1}H NMR Spectra for the $FA_{1-x}en_xPb_{\eta-y}Sn_yBr_3$ (η = 1-0.5x) family of perovskites (pg. S23-S26)

Figure S6: A plot of the Measured Unit Cell Volumes for the $FA_{1-x}en_xPb_{\eta-y}Sn_yBr_3$ (η = 1-0.5x) Family of Perovskites (pg. S27)

Note S1 (pg. S27)

Figure S7-S12: Results from Reverse Monte Carlo Analyses on Experimental Pair Distribution Function Data (Pg. S28-S34)

Figure S13: Diffuse Reflectance UV-Vis Spectra for the $FA_{1-x}en_xPb_{\eta-y}Sn_yBr_3$ (η = 1-0.5x) family of perovskites (pg. S35)

Figures S14-S19: Experimental Powder Diffraction Patterns for the $FA_{1-x}en_xPb_{\eta-y}Sn_yBr_3$ (η = 1-0.5x) family of perovskites (Pg. S36-S38)

Note S2-S6 (pg. S36-S38)

Figures S20-S27: Select Experimental Powder Diffraction Patterns for the $FA_{1-x}en_xPb_{\eta-y}Sn_yBr_3$ (η = 1-0.5x) Compounds after an initial and at least 24 hours of exposure to air (pg. S39-S42)

Figure S28: Photograph of $FASnBr_3$ and $FA_{0.8}en_{0.2}Sn_{0.9}Br_3$ after initial and 4 hours of exposure to air. (pg. S43)

Note S8: (pg. S43)

Figures S29-S32: Additional Fittings of X-ray Photoelectron Spectra (pg. S44-S47)

Figures S35-S36: (pg. S48) Experimental X-ray powder diffraction patterns for the as-synthesized $CsSnBr_3$ and Cs_2SnBr_6

Additional References: (pg. S49)

Additional Synthetic Procedures:

Materials: The materials, $Pb(CH_3COO)_2 \cdot 3H_2O$ (Sigma Aldrich; 98 wt. %), $SnCl_2 \cdot 2H_2O$ (Sigma Aldrich; 98 wt.%), Formamidine acetate salt ($FACH_3COO$; TCI; > 98 wt.%), hypophosphourous acid (H_3PO_2; Sigma Aldrich; 50 wt.% in H_2O), hydrobromic acid (Thermo Fisher; 48 wt.% in H_2O), and 1,2-diaminoethane (ethylenediamine; Sigma Aldrich > 99 wt.%) were used as received.

Synthesis of $FAPb_{0.2}Sn_{0.8}Br_3$: 2 mmol (758.7 mg) of $Pb(CH_3COO)_2 \cdot 3H_2O$ and 1 mmol (225.7 mg) of $SnCl_2 \cdot 2H_2O$ was added to a 20 mL vial and to it 1 mL of hypophosphourous acid (H_3PO_2) and 6 mL of Hydrobromic acid (HBr) was added. The reaction was placed on a hotplate set ~120°C under constant stirring until the solid dissolved. Once dissolved 3 mmol (312.3 mg) of Formamidine acetate salt ($FACH_3COO$) was added to the hot solution followed by rapid precipitation of a deep red solid which redissolved upon continued stirring. Once fully dissolved, the hotplate was turned off and the reaction was allowed to cool naturally to room temperature. The perovskite phase was extracted from the solution as a deep red cuboctahedral crystals for further experiments.

Synthesis of $FA_{0.89}en_{0.11}Pb_{0.76}Sn_{0.19}Br_3$: 2 mmol (758.7 mg) of $Pb(CH_3COO)_2 \cdot 3H_2O$ and 1 mmol (225.7 mg) of $SnCl_2 \cdot 2H_2O$ was added to a 20 mL vial and to it 6 mL of hydrobromic acid (HBr). The reaction was heated on a hotplate set to ~120°C under constant stirring. In a separate vial, 1 mL of hypophosphorous acid (H_3PO_2) was added followed by 20 µL of ethylenediamine to neutralize it. The resulting solution was then added to the reaction once all the solid was dissolved. After the addition of the H_3PO_2/en^{2+} solution, 3 mmol (312.3 mg) of $FACH_3COO$ salt was added followed by a rapid precipitation of a red solid. Upon continued heating and stirring the solid redissolved. Once fully dissolved, hotplate was turned off and the reaction was allowed to cool naturally to room temperature where the phase was extracted as red octahedral crystals.

Synthesis of $FA_{0.72}en_{0.24}Pb_{0.69}Sn_{0.18}Br_3$: 2 mmol (758.7 mg) of $Pb(CH_3COO)_2 \cdot 3H_2O$ and 1 mmol (225.7 mg) of $SnCl_2 \cdot 2H_2O$ was added to a 20 mL vial and to it 6 mL of hydrobromic acid (HBr). The reaction was heated on a hotplate set to ~120°C under constant stirring. In a separate vial, 1 mL of hypophosphorous acid (H_3PO_2) was added followed by 50 µL of ethylenediamine to neutralize it. The resulting solution was then added to the reaction once all the solid was dissolved.

After the addition of the H_3PO_2/en^{2+} solution, 3 mmol (312.3 mg) of $FACH_3COO$ salt was added followed by a rapid precipitation of an orange-red solid. Upon continued heating and stirring the solid redissolved. Once fully dissolved, hotplate was turned off and the reaction was allowed to cool naturally to room temperature, where the perovskite phase was extracted as orange-red octahedral crystals.

Synthesis of $FA_{0.65}en_{0.35}Pb_{0.65}Sn_{0.18}Br_3$: 2 mmol (758.7 mg) of $Pb(CH_3COO)_2·3H_2O$ and 1 mmol (225.7 mg) of $SnCl_2·2H_2O$ was added to a 20 mL vial and to it 6 mL of hydrobromic acid (HBr). The reaction was heated on a hotplate set to ~120°C under constant stirring. In a separate vial, 1 mL of hypophosphorous acid (H_3PO_2) was added followed by 75 μL of ethylenediamine to neutralize it. The resulting solution was then added to the reaction once all the solid was dissolved. After the addition of the H_3PO_2/en^{2+} solution, 3 mmol (312.3 mg) of $FACH_3COO$ salt was added followed by a rapid precipitation of an orange solid. Upon continued heating and stirring the solid redissolved. Once fully dissolved, hotplate was turned off and the reaction was allowed to cool naturally to room temperature, where the perovskite phase was extracted as orange octahedral crystals.

Synthesis of $FA_{0.55}en_{0.45}Pb_{0.61}Sn_{0.17}Br_3$: 2 mmol (758.7 mg) of $Pb(CH_3COO)_2·3H_2O$ and 1 mmol (225.7 mg) of $SnCl_2·2H_2O$ was added to a 20 mL vial and to it 6 mL of hydrobromic acid (HBr). The reaction was heated on a hotplate set to ~120°C under constant stirring. In a separate vial, 1 mL of hypophosphorous acid (H_3PO_2) was added followed by 100 μL of ethylenediamine to neutralize it. The resulting solution was then added to the reaction once all the solid was dissolved. After the addition of the H_3PO_2/en^{2+} solution, 3 mmol (312.3 mg) of $FACH_3COO$ salt was added followed by a rapid precipitation of an yellow solid, upon continued heating and stirring the solid redissolved. Once fully dissolved, the hotplate was turned off and the reaction was allowed to cool naturally to room temperature, where the perovskite phase was extracted as yellow octahedral crystals.

Synthesis of $FAPb_{0.5}Sn_{0.5}Br_3$: 1 mmol (379.4 mg) of $Pb(CH_3COO)_2·3H_2O$ and 2 mmol (451.3 mg) of $SnCl_2·2H_2O$ was added to a 20 mL vial and to it 1 mL of hypophosphourous acid (H_3PO_2) and 4 mL of Hydrobromic acid (HBr) was added. The reaction was placed on a hotplate set ~120°C

under constant stirring until the solid dissolved. Once dissolved 3 mmol (312.3 mg) of Formamidine acetate salt ($FACH_3COO$) was added to the hot solution followed by rapid precipitation of a deep red solid which redissolved upon continued stirring. Once fully dissolved, the hotplate was turned off and the reaction was allowed to cool naturally to room temperature. The perovskite phase was extracted from the solution as deep red cuboctahedral crystals for further experiments.

Synthesis of $FA_{0.88}en_{0.12}Pb_{0.41}Sn_{0.52}Br_3$: 1 mmol (379.4 mg) of $Pb(CH_3COO)_2{\cdot}3H_2O$ and 2 mmol (451.3 mg) of $SnCl_2{\cdot}2H_2O$ was added to a 20 mL vial and to it 4 mL of hydrobromic acid (HBr). The reaction was heated on a hotplate set to ~120°C under constant stirring. In a separate vial, 1 mL of hypophosphorous acid (H_3PO_2) was added followed by 20 μL of ethylenediamine to neutralize it. The resulting solution was then added to the reaction once all the solid was dissolved. After the addition of the H_3PO_2/en^{2+} solution, 3 mmol (312.3 mg) of $FACH_3COO$ salt was added followed by a rapid precipitation of a red solid. Upon continued heating and stirring the solid redissolved. Once fully dissolved, hotplate was turned off and the reaction was allowed to cool naturally to room temperature where the phase was extracted as red octahedral crystals.

Synthesis of $FA_{0.81}en_{0.19}Pb_{0.43}Sn_{0.47}Br_3$: 1 mmol (379.4 mg) of $Pb(CH_3COO)_2{\cdot}3H_2O$ and 2 mmol (451.3 mg) of $SnCl_2{\cdot}2H_2O$ was added to a 20 mL vial and to it 4 mL of hydrobromic acid (HBr). The reaction was heated on a hotplate set to ~120°C under constant stirring. In a separate vial, 1 mL of hypophosphorous acid (H_3PO_2) was added followed by 50 μL of ethylenediamine to neutralize it. The resulting solution was then added to the reaction once all the solid was dissolved. After the addition of the H_3PO_2/en^{2+} solution, 3 mmol (312.3 mg) of $FACH_3COO$ salt was added followed by a rapid precipitation of a red solid. Upon continued heating and stirring the solid redissolved. Once fully dissolved, hotplate was turned off and the reaction was allowed to cool naturally to room temperature where the phase was extracted as orange-red octahedral crystals.

Synthesis of $FA_{0.69}en_{0.31}Pb_{0.42}Sn_{0.43}Br_3$: 1 mmol (379.4 mg) of $Pb(CH_3COO)_2{\cdot}3H_2O$ and 2 mmol (451.3 mg) of $SnCl_2{\cdot}2H_2O$ was added to a 20 mL vial and to it 4 mL of hydrobromic acid (HBr). The reaction was heated on a hotplate set to ~120°C under constant stirring. In a separate vial, 1 mL of hypophosphorous acid (H_3PO_2) was added followed by 75 μL of ethylenediamine to

neutralize it. The resulting solution was then added to the reaction once all the solid was dissolved. After the addition of the H_3PO_2/en^{2+} solution, 3 mmol (312.3 mg) of $FACH_3COO$ salt was added followed by a rapid precipitation of a red solid. Upon continued heating and stirring the solid redissolved. Once fully dissolved, hotplate was turned off and the reaction was allowed to cool naturally to room temperature where the phase was extracted as yellow-orange octahedral crystals.

Synthesis of $FA_{0.81}en_{0.19}Pb_{0.43}Sn_{0.47}Br_3$: 1 mmol (379.4 mg) of $Pb(CH_3COO)_2 \cdot 3H_2O$ and 2 mmol (451.3 mg) of $SnCl_2 \cdot 2H_2O$ was added to a 20 mL vial and to it 4 mL of hydrobromic acid (HBr). The reaction was heated on a hotplate set to ~120°C under constant stirring. In a separate vial, 1 mL of hypophosphorous acid (H_3PO_2) was added followed by 100 µL of ethylenediamine to neutralize it. The resulting solution was then added to the reaction once all the solid was dissolved. After the addition of the H_3PO_2/en^{2+} solution, 3 mmol (312.3 mg) of $FACH_3COO$ salt was added followed by a rapid precipitation of a red solid. Upon continued heating and stirring the solid redissolved. Once fully dissolved, hotplate was turned off and the reaction was allowed to cool naturally to room temperature where the phase was extracted as yellow octahedral crystals.

Synthesis of $FAPb_{0.24}Sn_{0.76}Br_3$: 0.5 mmol (189.7 mg) of $Pb(CH_3COO)_2 \cdot 3H_2O$ and 2.5 mmol (564.1 mg) of $SnCl_2 \cdot 2H_2O$ was added to a 20 mL vial and to it 1 mL of hypophosphourous acid (H_3PO_2) and 3 mL of Hydrobromic acid (HBr) was added. The reaction was placed on a hotplate set ~120°C under constant stirring until the solid dissolved. Once dissolved 3 mmol (312.3 mg) of Formamidine acetate salt ($FACH_3COO$) was added to the hot solution followed by rapid precipitation of a deep red solid which redissolved upon continued stirring. Once fully dissolved, the hotplate was turned off and the reaction was allowed to cool naturally to room temperature. The perovskite phase was extracted from the solution as deep red rod-like crystals for further experiments.

Synthesis of $FA_{0.91}en_{0.09}Pb_{0.24}Sn_{0.72}Br_3$: 0.5 mmol (189.7 mg) of $Pb(CH_3COO)_2 \cdot 3H_2O$ and 2.5 mmol (546.1 mg) of $SnCl_2 \cdot 2H_2O$ was added to a 20 mL vial and to it 3 mL of hydrobromic acid (HBr). The reaction was heated on a hotplate set to ~120°C under constant stirring. In a separate vial, 1 mL of hypophosphorous acid (H_3PO_2) was added followed by 20 µL of ethylenediamine to neutralize it. The resulting solution was then added to the reaction once all the solid was dissolved.

After the addition of the H_3PO_2/en^{2+} solution, 3 mmol (312.3 mg) of $FACH_3COO$ salt was added followed by a rapid precipitation of a red solid. Upon continued heating and stirring the solid redissolved. Once fully dissolved, hotplate was turned off and the reaction was allowed to cool naturally to room temperature where the phase was extracted as red octahedral crystals.

Synthesis of $FA_{0.77}en_{0.23}Pb_{0.22}Sn_{0.66}Br_3$: 0.5 mmol (189.7 mg) of $Pb(CH_3COO)_2·3H_2O$ and 2.5 mmol (546.1 mg) of $SnCl_2·2H_2O$ was added to a 20 mL vial and to it 3 mL of hydrobromic acid (HBr). The reaction was heated on a hotplate set to ~120°C under constant stirring. In a separate vial, 1 mL of hypophosphorous acid (H_3PO_2) was added followed by 50 μL of ethylenediamine to neutralize it. The resulting solution was then added to the reaction once all the solid was dissolved. After the addition of the H_3PO_2/en^{2+} solution, 3 mmol (312.3 mg) of $FACH_3COO$ salt was added followed by a rapid precipitation of a red solid. Upon continued heating and stirring the solid redissolved. Once fully dissolved, hotplate was turned off and the reaction was allowed to cool naturally to room temperature where the phase was extracted as orange-red octahedral crystals.

Synthesis of $FA_{0.72}en_{0.28}Pb_{0.22}Sn_{0.64}Br_3$: 0.5 mmol (189.7 mg) of $Pb(CH_3COO)_2·3H_2O$ and 2.5 mmol (546.1 mg) of $SnCl_2·2H_2O$ was added to a 20 mL vial and to it 3 mL of hydrobromic acid (HBr). The reaction was heated on a hotplate set to ~120°C under constant stirring. In a separate vial, 1 mL of hypophosphorous acid (H_3PO_2) was added followed by 75 μL of ethylenediamine to neutralize it. The resulting solution was then added to the reaction once all the solid was dissolved. After the addition of the H_3PO_2/en^{2+} solution, 3 mmol (312.3 mg) of $FACH_3COO$ salt was added followed by a rapid precipitation of a red solid. Upon continued heating and stirring the solid redissolved. Once fully dissolved, hotplate was turned off and the reaction was allowed to cool naturally to room temperature where the phase was extracted as orange octahedral crystals.

Synthesis of $FA_{0.66}en_{0.34}Pb_{0.22}Sn_{0.61}Br_3$: 0.5 mmol (189.7 mg) of $Pb(CH_3COO)_2·3H_2O$ and 2.5 mmol (546.1 mg) of $SnCl_2·2H_2O$ was added to a 20 mL vial and to it 3 mL of hydrobromic acid (HBr). The reaction was heated on a hotplate set to ~120°C under constant stirring. In a separate vial, 1 mL of hypophosphorous acid (H_3PO_2) was added followed by 100 μL of ethylenediamine to neutralize it. The resulting solution was then added to the reaction once all the solid was dissolved. After the addition of the H_3PO_2/en^{2+} solution, 3 mmol (312.3 mg) of $FACH_3COO$ salt

was added followed by a rapid precipitation of a red solid. Upon continued heating and stirring the solid redissolved. Once fully dissolved, hotplate was turned off and the reaction was allowed to cool naturally to room temperature where the phase was extracted as yellow octahedral crystals.

Synthesis of $FASnBr_3$: 3 mmol (677 mg) of $SnCl_2{\cdot}2H_2O$ was added to a 20 mL vial and to it 1 mL of hypophosphourous acid (H_3PO_2) and 3 mL of Hydrobromic acid (HBr) was added. The reaction was placed on a hotplate set ~120°C under constant stirring until the solid dissolved. Once dissolved 3 mmol (312.3 mg) of Formamidine acetate salt ($FACH_3COO$) was added to the hot solution followed by rapid precipitation of a deep red solid which redissolved upon continued stirring. Once fully dissolved, the hotplate was turned off and the reaction was allowed to cool naturally to room temperature. The perovskite phase was extracted from the solution yellow rod-like crystals for further experiments.

Synthesis of $FA_{0.89}en_{0.11}Sn_{0.94}Br_3$: 3 mmol (677 mg) of $SnCl_2{\cdot}2H_2O$ was added to a 20 mL vial and to it 3 mL of hydrobromic acid (HBr). The reaction was heated on a hotplate set to ~120°C under constant stirring. In a separate vial, 1 mL of hypophosphorous acid (H_3PO_2) was added followed by 20 μL of ethylenediamine to neutralize it. The resulting solution was then added to the reaction once all the solid was dissolved. After the addition of the H_3PO_2/en^{2+} solution, 3 mmol (312.3 mg) of $FACH_3COO$ salt was added followed by a rapid precipitation of a red solid. Upon continued heating and stirring the solid redissolved. Once fully dissolved, hotplate was turned off and the reaction was allowed to cool naturally to room temperature where the phase was extracted as yellow octahedral crystals.

Synthesis of $FA_{0.80}en_{0.20}Sn_{0.90}Br_3$: 3 mmol (677 mg) of $SnCl_2{\cdot}2H_2O$ was added to a 20 mL vial and to it 3 mL of hydrobromic acid (HBr). The reaction was heated on a hotplate set to ~120°C under constant stirring. In a separate vial, 1 mL of hypophosphorous acid (H_3PO_2) was added followed by 50 μL of ethylenediamine to neutralize it The resulting solution was then added to the reaction once all the solid was dissolved. After the addition of the H_3PO_2/en^{2+} solution, 3 mmol (312.3 mg) of $FACH_3COO$ salt was added followed by a rapid precipitation of a red solid. Upon continued heating and stirring the solid redissolved. Once fully dissolved, hotplate was turned off

and the reaction was allowed to cool naturally to room temperature where the phase was extracted as yellow octahedral crystals.

Synthesis of $FA_{0.72}en_{0.28}Sn_{0.86}Br_3$: 3 mmol (677 mg) of $SnCl_2 \cdot 2H_2O$ was added to a 20 mL vial and to it 3 mL of hydrobromic acid (HBr). The reaction was heated on a hotplate set to ~120°C under constant stirring. In a separate vial, 1 mL of hypophosphorous acid (H_3PO_2) was added followed by 75 µL of ethylenediamine to neutralize it. The resulting solution was then added to the reaction once all the solid was dissolved. After the addition of the H_3PO_2/en^{2+} solution, 3 mmol (312.3 mg) of $FACH_3COO$ salt was added followed by a rapid precipitation of a red solid. Upon continued heating and stirring the solid redissolved. Once fully dissolved, hotplate was turned off and the reaction was allowed to cool naturally to room temperature where the phase was extracted as yellow octahedral crystals.

Synthesis of $FA_{0.67}en_{0.33}Sn_{0.84}Br_3$: 3 mmol (677 mg) of $SnCl_2 \cdot 2H_2O$ was added to a 20 mL vial and to it 3 mL of hydrobromic acid (HBr). The reaction was heated on a hotplate set to ~120°C under constant stirring. In a separate vial, 1 mL of hypophosphorous acid (H_3PO_2) was added followed by 100 µL of ethylenediamine to neutralize it. The resulting solution was then added to the reaction once all the solid was dissolved. After the addition of the H_3PO_2/en^{2+} solution, 3 mmol (312.3 mg) of $FACH_3COO$ salt was added followed by a rapid precipitation of a red solid. Upon continued heating and stirring the solid redissolved. Once fully dissolved, hotplate was turned off and the reaction was allowed to cool naturally to room temperature where the phase was extracted as yellow octahedral crystals.

Pair Distribution Function (PDF) Calculations:

The total scattering pair distribution function is a powerful method for examining the local structures of solids without the precondition of crystallinity. Since the relatively weak diffuse scatting and traditional Bragg reflections (if present) are treated equally, the technique can be applied to crystalline and non-crystalline materials alike. For crystalline materials, modeling of the

PDF curve can draw a contrast, if any, between the local structure and the long-range structure obtained from traditional crystallography experiments. The PDF (G(*r*)) is typically calculated from

experimental high-resolution X-ray powder diffraction patterns considering only the coherent scattering contributions. The total scattering structure function (S(Q)) is obtained from the coherent scattering intensity $I_c(Q)$ through eq. S1.

$$S(Q) = \frac{I_c - \sum c_i |f_i(Q)|^2}{\left|\sum c_i f_i(Q)\right|^2} + 1$$
eq. S1

The coherent part of the intensity function is determined through appropriate background subtraction taking into account scattering from the container, contributions from air scattering, and additional effects. The $S(Q)$ is normalized to the flux of the radiation source and the total number of atoms. The c_i and $f_i(Q)$ terms in eq. S1 are the atomic concentration and the X-ray atomic form factor for the atomic species of type *i* and the momentum transfer (Q) which is calculated through eq. S2.

$$Q = \frac{4\pi \sin\theta}{\lambda}$$
eq. S2

It is important to note that the momentum transfer unit (Å^{-1}) normalizes for the wavelength of radiation, making it comparable across radiation sources. Once calculated, the $S(Q)$ is used to calculate the atomic pair distribution function ($G(r)$) through the Fourier transformation of the expression $Q(S(Q)$-1) as shown in eq. S3.

$$G(r) = \frac{2}{\pi} \int_{Q_{min}}^{Q_{max}} Q(S(Q) - 1)\sin(Qr)dQ$$
eq. S3

The atomic pair distribution function is then also related to the atomic density (ρ_0) and the atomic pair density ($\rho(r)$, where r is a unit of distance typically expressed as angstroms). Eq. S4 describes the precise relationship.

$$G(r) = 4\pi r(\rho(r) - \rho_0)$$
eq. S4

In observing the most practical form of the $G(r)$, it is apparent that the function expresses the density of atoms residing a distance r from an arbitrary center, reference, or designated atom, which has a density ρ_0. Typically, the $G(r)$ is modeled against the predicted PDF curve generated by perturbing the known average structure, or isostructural material. Of importance is the resolution of the $G(r)$ (eq. S5) which limits the smallest variation in distance vectors that can reasonably be observed. The resolution is ultimately determined by the measured $Q_{\max}$ which

ideally would be > 20 Å^{-1} and is easier to obtain through shorter wavelength radiation as implied by eq. S2. However, the needed real space resolution (δr) is entirely dependent on the features in the structure that are desired to be observed.

$$\delta r = \frac{2\pi}{Q_{max}} \qquad \text{eq. S5}$$

Diffuse Reflectance UV-Vis:

The products were mechanically ground in an agate mortar and pestle inside of a nitrogen-filled glovebox until a powder-like consistency was achieved. The powder was then combined with quantities of barium sulfate ($BaSO_4$) and mixed until a homogeneous powder was once more received. The $BaSO_4$ served to dilute the absorbance of the sample in the desired energy range such that a reflection geometry could be used for the data collection. $BaSO_4$ also served as the 100% reflectance standard while an empty chamber served as the 0% reflectance standard. The optical absorption data were collected at room temperature in the desired energy range to observe the sample absorption edge using a Cary 5000 UV-Vis NIR double-beam spectrophotometer equipped with a monochromator and an integrating sphere detector. The data were then used to estimate the band gap of each material through the use of the Kubelka-Munk transformation and the linear extrapolation of the absorption edge and the observed background.[1]

Synthesis of Sn^{2+} and Sn^{4+} Standards for X-ray Photoelectron Spectroscopy:

Synthesis of $CsSnBr_3$: $CsSnBr_3$ was synthesized mechanochemically from the binary halide salt precursors CsBr and $SnBr_2$ in a 1 to 1 molar ratio: total mass ~1 g. The salts were added to a 30 mL, stainless steel mill and subsequently loaded with 3 mm stainless steel bearings, filling ~ ½ of the total volume of the mill. The mill was then loaded into an MM-400 mixer mill, purchased from Retsch and reacted with a frequency of 30 Hz for ~ 90 minutes. The resulting black powder was then tested via X-ray powder diffraction to confirm formation of the desired phase (Figure S35).

Synthesis of Cs_2SnBr_6: Cs_2SnBr_6 was synthesized mechanochemically from the necessary binary halide salt precursors CsBr and $SnBr_4$ in a 2 to 1 molar ratio: total mass ~1 g. the salts were then added to a 30 mL, stainless steel mill and subsequently loaded with 3 mm stainless steel bearings, filling ~ ½ of the total volume of the mill. The mill was then inserted into a MM-400 mixer mill,

purchased from Retsch, and reacted with a frequency of 30 Hz for ~ 270 minutes. The resulting powder was then tested via X-ray powder diffraction, confirming the formation of Cs_2SnBr_6 alongside a small amount of unreacted CsBr (Figure S36)

Table S1: Crystallographic information and refinement parameters for the $FA_{1-x}en_xPb_{\eta-y}Sn_yBr_3$ (η = 1-0.5x) family of perovskites where χ_{Sn}= 0.2.

Formula	$FAPb_{0.84}Sn_{0.16}Br_3$	$FA_{0.89}en_{0.11}Pb_{0.76}Sn_{0.19}Br_3$	$FA_{0.76}en_{0.24}Pb_{0.69}Sn_{0.18}Br_3$	$FA_{0.65}en_{0.35}Pb_{0.65}Sn_{0.18}Br_3$	$FA_{0.55}en_{0.45}Pb_{0.61}Sn_{0.17}Br_3$
Formula weight (g/mol)	472.79	464.9	454.5	446.0	441.4
Temperature (K)			295 K		
Wavelength (Å)	0.56083	0.71073	0.71073	0.71073	0.71073
Crystal system			Cubic		
Space group			$Pm\bar{3}m$		
a/*b*/*c* (Å)	5.9577(13)	6.0203(6)	6.0133(4)	6.0379(7)	6.0566(6)
V (Å^3)	211.46(14)	218.20(4)	217.44(3)	220.12(4)	222.17(4)
Z			1		
ρ_{calc} (g/cm^3)	3.713	3.5377	3.4709	3.3647	3.2749
μ (mm^{-1})	16.858	28.757	27.697	26.482	25.44
Reflections collected	1319	782	3810	979	929
Independent reflections	148 [R_{int} = 0.0150]	151 [R_{int} = 0.0131]	156 [R_{int} = 0.0325]	152 [R_{int} = 0.0168]	76 [R_{int} = 0.0217]
Completeness	100% (θ = 19.664°)	98% (θ = 37.43°)	100% (θ = 37.68°)	98% (θ = 37.5°)	98% (θ = 37.37°)
Data / restraints / parameters	148 / 1 / 9	151 / 1 / 9	156 / 1 / 7	152 / 1 / 7	76 / 1 / 7
Goodness-of-fit	1.336	1.37	1.54	1.71	2.63
Final R indices [I > 2σ(I)]	R_{obs} = 0.0263, wR_{obs} = 0.0750	R_{obs} = 0.0326, wR_{obs} = 0.0762	R_{obs} = 0.0355, wR_{obs} = 0.0919	R_{obs} = 0.0413, wR_{obs} = 0.1065	R_{obs} = 0.0426, wR_{obs} = 0.1301
R indices [all data]	R_{all} = 0.0267, wR_{all} = 0.0751	R_{all} = 0.0360, wR_{all} = 0.0774	R_{all} = 0.0464, wR_{all} = 0.0952	R_{all} = 0.0596, wR_{all} = 0.1103	R_{all} = 0.0445, wR_{all} = 0.1307
Largest peak/hole (e$^-$/Å^3)	0.865 / -1.417	0.74 / -0.81	2.13 / -0.56	2.19 / -0.47	2.03 / -0.67

$R = \Sigma||F_o|-|F_c|| / \Sigma|F_o|$, $wR = \{\Sigma[w(|F_o|^2 - |F_c|^2)^2] / \Sigma[w(|F_o|^4)]\}^{1/2}$ and $w=1/[\sigma^2(Fo^2)+(0.0401P)^2+0.1385P]$ where $P=(Fo^2+2Fc^2)/3$

Table S2: Crystallographic information and refinement parameters for the $FA_{1-x}en_xPb_{\eta-y}Sn_yBr_3$ (η = 1-0.5x) family of perovskites where χ_{Sn}= 0.5.

Formula	$FAPb_{0.50}Sn_{0.50}Br_3$	$FA_{0.88}en_{0.12}Pb_{0.42}Sn_{0.52}Br_3$	$FA_{0.81}en_{0.19}Pb_{0.43}Sn_{0.47}Br_3$	$FA_{0.69}en_{0.31}Pb_{0.42}Sn_{0.43}Br_3$	$FA_{0.64}en_{0.36}Pb_{0.42}Sn_{0.40}Br_3$
Formula weight (g/mol)	442.70	435.4	433.6	428.8	425.8
Temperature (K)			295 K		
Wavelength (Å)	0.56083	0.71073	0.71073	0.71073	0.71073
Crystal system			Cubic		
Space group			$Pm\bar{3}m$		
a/*b*/*c* (Å)	5.9617(18)	6.0075(7)	6.0236(7)	6.0413(3)	6.0315(10)
V (Å^3)	211.89(19)	216.81(4)	218.56(4)	220.491(19)	219.42(6)
Z			1		
ρ_{calc} (g/cm^3)	3.469	3.3348	3.2945	3.2291	3.2221
μ (mm^{-1})	13.687	23.412	23.302	22.83	22.872
Reflections collected	820	752	980	3130	755
Independent reflections	105 [R_{int} = 0.0166]	151 [R_{int} = 0.0122]	153 [R_{int} = 0.0177]	159 [R_{int} = 0.0263]	150 [R_{int} = 0.0310]
Completeness	100% (θ = 19.664°)	99% (θ = 37.52°)	99% (θ = 37.4°)	98% (θ = 38.09°)	98% (θ = 37.05°)
Data / restraints / parameters	105 / 1 / 9	151 / 1 / 7	153 / 1 / 7	159 / 1 / 8	150 / 1 / 7
Goodness-of-fit	1.219	1.35	1.47	1.61	1.58
Final R indices [I > 2σ(I)]	R_{obs} = 0.0303, wR_{obs} = 0.0829	R_{obs} = 0.0327, wR_{obs} = 0.0795	R_{obs} = 0.0353, wR_{obs} = 0.0950	R_{obs} = 0.0418, wR_{obs} = 0.0992	R_{obs} = 0.0481, wR_{obs} = 0.1186
R indices [all data]	R_{all} = 0.0303, wR_{all} = 0.0829	R_{all} = 0.0467, wR_{all} = 0.0834	R_{all} = 0.0560, wR_{all} = 0.1000	R_{all} = 0.0589, wR_{all} = 0.1030	R_{all} = 0.0983, wR_{all} = 0.1270
Largest peak/hole (e$^-$/Å^3)	0.922 / -0.926	0.89 / -0.37	1.74 / -0.51	1.81 / -0.50	3.98 / -0.97

$R = \Sigma||F_o|-|F_c|| / \Sigma|F_o|$, $wR = \{\Sigma[w(|F_o|^2 - |F_c|^2)^2] / \Sigma[w(|F_o|^4)]\}^{1/2}$ and $w=1/[\sigma^2(Fo^2)+(0.0401P)^2+0.1385P]$ where $P=(Fo^2+2Fc^2)/3$

Table S3: Crystallographic information and refinement parameters for the $FA_{1-x}en_xPb_{\eta-y}Sn_yBr_3$ (η = 1-0.5x) family of perovskites where χ_{Sn}= 0.75.

Formula	$FAPb_{0.21}Sn_{0.79}Br_3$	$FA_{0.91}en_{0.09}Pb_{0.24}Sn_{0.72}Br_3$	$FA_{0.77}en_{0.23}Pb_{0.22}Sn_{0.66}Br_3$	$FA_{0.72}en_{0.28}Pb_{0.22}Sn_{0.64}Br_3$	$FA_{0.66}en_{0.34}Pb_{0.22}Sn_{0.61}Br_3$
Formula weight (g/mol)	417.04	420.5	433.6	411.8	425.8
Temperature (K)			295 K		
Wavelength (Å)	0.56083	0.71073	0.71073	0.71073	0.71073
Crystal system			Cubic		
Space group			$Pm\overline{3}m$		
a/*b*/*c* (Å)	5.959(2)	5.9970(8)	6.0213(7)	6.0411(5)	6.0644(7)
V (Å^3)	211.6(3)	215.68(5)	218.31(4)	220.47(3)	223.03(4)
Z			1		
ρ_{calc} (g/cm^3)	3.272	3.2375	3.1425	3.1018	3.0428
μ (mm^{-1})	11.044	20.568	19.849	19.678	19.284
Reflections collected	709	728	770	980	781
Independent reflections	85 [R_{int} = 0.0166]	91 [R_{int} = 0.0322]	151 [R_{int} = 0.0161]	153 [R_{int} = 0.0177]	51 [R_{int} = 0.0303]
Completeness	100% (θ = 19.664°)	98% (θ = 36.76°)	98% (θ = 37.42°)	100% (θ = 37.27°)	98% (θ = 37.52°)
Data / restraints / parameters	85 / 1 / 9	91 / 1 / 8	151 / 1 / 7	153 / 1 / 7	51 / 1 / 8
Goodness-of-fit	1.306	2.37	1.58	1.64	3.12
Final R indices [I > 2σ(I)]	R_{obs} = 0.0463, wR_{obs} = 0.1220	R_{obs} = 0.0508, wR_{obs} = 0.1241	R_{obs} = 0.0402, wR_{obs} = 0.1058	R_{obs} = 0.0450, wR_{obs} = 0.1099	R_{obs} = 0.0679, wR_{obs} = 0.1628
R indices [all data]	R_{all} = 0.0479, wR_{all} = 0.1255	R_{all} = 0.0508, wR_{all} = 0.1241	R_{all} = 0.0633, wR_{all} = 0.1112	R_{all} = 0.0771, wR_{all} = 0.1187	R_{all} = 0.0685, wR_{all} = 0.1629
Largest peak/hole (e$^-$/Å^3)	0.781 / -1.236	0.64 / -1.27	1.66 / -0.53	1.97 / -0.59	0.90 / -0.28

$R = \Sigma||F_o|-|F_c|| / \Sigma|F_o|$, $wR = \{\Sigma[w(|F_o|^2 - |F_c|^2)^2] / \Sigma[w(|F_o|^4)]\}^{1/2}$ and $w=1/[\sigma^2(Fo^2)+(0.0401P)^2+0.1385P]$ where $P=(Fo^2+2Fc^2)/3$

Table S4: Crystallographic information and refinement parameters for the $FA_{1-x}en_xPb_{\eta-y}Sn_yBr_3$ (η = 1-0.5x) family of perovskites where χ_{Sn}= 1.

Formula	$FA_{0.89}en_{0.11}Sn_{0.94}Br_3$	$FA_{0.80}en_{0.20}Sn_{0.90}Br_3$	$FA_{0.72}en_{0.28}Sn_{0.86}Br_3$	$FA_{0.67}en_{0.33}Sn_{0.84}Br_3$
Formula weight (g/mol)	387.1	395.2	391.8	389.6
Temperature (K)				
Wavelength (Å)	0.71073	0.71073	0.71073	0.71073
Crystal system				
Space group				
a/*b*/*c* (Å)	6.0226(7)	6.0324(7)	6.0349(14)	6.0371(10)
V (Å^3)	218.45(4)	219.52	219.79(9)	220.03(6)
Z				
ρ_{calc} (g/cm^3)	2.9424	2.9896	2.9601	2.9399
μ (mm^{-1})	16.394	16.203	16.072	15.98
Reflections collected	967	1196	697	771
Independent reflections	152 [R_{int} = 0.0169]	153 [R_{int} = 0.0242]	62 [R_{int} = 0.0200]	63 [R_{int} = 0.0271
Completeness	99% (θ = 37.41°)	99% (θ = 37.34°)	98% (θ = 37.53°)	99% (θ = 37.3°)
Data / restraints / parameters	152 / 1 / 7	153 / 1 / 7	62 / 1 / 7	63 / 1 / 9
Goodness-of-fit	2.65	2.00	2.85	2.97
Final R indices [I > 2σ(I)]	R_{obs} = 0.0586, wR_{obs} = 0.1835	R_{obs} = 0.0501, wR_{obs} = 0.1495	R_{obs} = 0.0481, wR_{obs} = 0.1411	R_{obs} = 0.0576, wR_{obs} = 0.1540
R indices [all data]	R_{all} = 0.0881, wR_{all} = 0.1888	R_{all} = 0.1002, wR_{all} = 0.1589	R_{all} = 0.0480, wR_{all} = 0.1411	R_{all} = 0.0600, wR_{all} = 0.1549
Largest peak/hole (e$^-$/Å^3)	2.89 / -1.36	3.19 / -0.82	0.88 / -0.51	0.76 / -0.17

$R = \Sigma||F_o|-|F_c|| / \Sigma|F_o|$, $wR = \{\Sigma[w(|F_o|^2 - |F_c|^2)^2] / \Sigma[w(|F_o|^4)]\}^{1/2}$ and $w=1/[\sigma^2(Fo^2)+(0.0401P)^2+0.1385P]$ where $P=(Fo^2+2Fc^2)/3$

Table S5. Atomic coordinates (x10^4) and equivalent isotropic displacement parameters (Å^{2}x10^3) for the $FA_{1-x}en_xPb_{\eta-y}Sn_yBr_3$ (η = 1-0.5x) family of perovskites where χ_{Sn}= 0.2.

Label	x	y	z	Occupancy	U_{eq}*
			**$FAPb_{0.84}Sn_{0.16}Br_3$**		
Pb	5000	5000	5000	0.84	39(1)
Sn	5000	5000	5000	0.1599	39(1)
Br	0	5000	5000	1	84(1)
C	0	0	0	1	160(40)
N	2360(30)	0	0	0.3334	170(40)
			**$FA_{0.89}en_{0.11}Pb_{0.76}Sn_{0.19}Br_3$**		
Pb	5000	5000	5000	0.753	47(1)
Sn	5000	5000	5000	0.1868	47(1)
Br	0	5000	5000	1	100(1)
C	0	0	0	0.8894	166(19)
N	2408(2)	0	0	0.2936	160(40)
			**$FA_{0.76}en_{0.24}Pb_{0.69}Sn_{0.18}Br_3$**		
Pb	5000	5000	5000	0.6927	53(1)
Sn	5000	5000	5000	0.1848	53(1)
Br	0	5000	5000	1	113(1)
C	0	0	0	0.755	300(80)
N	2411(3)	0	0	0.2492	170(50)
			**$FA_{0.65}en_{0.35}Pb_{0.65}Sn_{0.18}Br_3$**		
Pb	5000	5000	5000	0.648	62(1)
Sn	5000	5000	5000	0.1769	62(1)
Br	0	5000	5000	1	129(1)
C	0	0	0	0.6504	280(90)
N	2401(3)	0	0	0.2146	190(60)
			**$FA_{0.55}en_{0.45}Pb_{0.61}Sn_{0.17}Br_3$**		
Pb	5000	5000	5000	0.6067	66(1)
Sn	5000	5000	5000	0.1677	66(1)
Br	0	5000	5000	1	144(2)
C	0	0	0	0.5491	310(120)
N	2394(4)	0	0	0.1816	180(90)

*U_{eq} is defined as one third of the trace of the orthogonalized U_{ij} tensor.

Table S6. Atomic coordinates (x10^4) and equivalent isotropic displacement parameters (Å^{2}x10^3) for the $FA_{1-x}en_xPb_{\eta-y}Sn_yBr_3$ (η = 1-0.5x) family of perovskites where χ_{Sn}= 0.5.

Label	x	y	z	Occupancy	U_{eq}*
		**$FAPb_{0.50}Sn_{0.50}Br_3$**			
Pb	5000	5000	5000	0.5002	46(1)
Sn	5000	5000	5000	0.5002	46(1)
Br	0	5000	5000	1	96(1)
C	0	0	0	1	160(40)
N	2390(30)	0	0	0.3334	210(70)
		**$FA_{0.88}en_{0.12}Pb_{0.42}Sn_{0.52}Br_3$**			
Pb	5000	5000	5000	0.4175	56(1)
Sn	5000	5000	5000	0.5222	56(1)
Br	0	5000	5000	1	116(1)
C	0	0	0	0.8795	230(40)
N	2413(2)	0	0	0.2902	200(40)
		**$FA_{0.81}en_{0.19}Pb_{0.43}Sn_{0.47}Br_3$**			
Pb	5000	5000	5000	0.4292	61(1)
Sn	5000	5000	5000	0.4742	61(1)
Br	0	5000	5000	1	125(1)
C	0	0	0	0.84	260(50)
N	2407(2)	0	0	0.296	230(60)
		**$FA_{0.69}en_{0.31}Pb_{0.42}Sn_{0.43}Br_3$**			
Pb	5000	5000	5000	0.4218	66(1)
Sn	5000	5000	5000	0.4252	66(1)
Br	0	5000	5000	1	135(1)
C	0	0	0	0.768	310(50)
N	2400(3)	0	0	0.2552	210(50)
		**$FA_{0.64}en_{0.36}Pb_{0.42}Sn_{0.40}Br_3$**			
Pb	5000	5000	5000	0.4225	71(1)
Sn	5000	5000	5000	0.3995	71(1)
Br	0	5000	5000	1	143(1)
C	0	0	0	0.6442	180(40)
N	2404(3)	0	0	0.2125	240(80)

*U_{eq} is defined as one third of the trace of the orthogonalized U_{ij} tensor.

Table S7. Atomic coordinates (x10^4) and equivalent isotropic displacement parameters (Å^{2}x10^3) for the $FA_{1-x}en_xPb_{\eta-y}Sn_yBr_3$ (η = 1-0.5x) family of perovskites where χ_{Sn}= 0.75.

Label	x	y	z	Occupancy	U_{eq}*
		**$FAPb_{0.24}Sn_{0.76}Br_3$**			
Pb	5000	5000	5000	0.21(3)	53(1)
Sn	5000	5000	5000	0.79(3)	53(1)
Br	0	5000	5000	1	120(2)
C	0	0	0	1	200(80)
N	2420(40)	0	0	0.3334	260(110)
		**$FA_{0.91}en_{0.09}Pb_{0.24}Sn_{0.72}Br_3$**			
Pb	5000	5000	5000	0.2369	58(1)
Sn	5000	5000	5000	0.7164	58(1)
Br	0	5000	5000	1	124(2)
C	0	0	0	0.9072	160(20)
N	2417(4)	0	0	0.2992	260(100)
		**$FA_{0.77}en_{0.23}Pb_{0.22}Sn_{0.66}Br_3$**			
Pb	5000	5000	5000	0.2204	66(1)
Sn	5000	5000	5000	0.6637	66(1)
Br	0	5000	5000	1	137(1)
C	0	0	0	0.7682	200(40)
N	2408(3)	0	0	0.2535	230(60)
		**$FA_{0.72}en_{0.28}Pb_{0.22}Sn_{0.64}Br_3$**			
Pb	5000	5000	5000	0.2246	73(1)
Sn	5000	5000	5000	0.6376	73(1)
Br	0	5000	5000	1	146(1)
C	0	0	0	0.7245	210(40)
N	2400(3)	0	0	0.2391	250(70)
		**$FA_{0.66}en_{0.34}Pb_{0.22}Sn_{0.61}Br_3$**			
Pb	5000	5000	5000	0.2202	117(2)
Sn	5000	5000	5000	0.6114	117(2)
Br	0	5000	5000	1	191(3)
C	0	0	0	0.6667	340(170)
N	2391(5)	0	0	0.22	500(1000)

*U_{eq} is defined as one third of the trace of the orthogonalized U_{ij} tensor.

Table S7. Atomic coordinates (x10^4) and equivalent isotropic displacement parameters (Å^{2}x10^3) for the $FA_{1-x}en_xPb_{\eta-y}Sn_yBr_3$ (η = 1-0.5x) family of perovskites where χ_{Sn}= 1.

**Label**	**x**	**y**	**z**	**Occupancy**	**U_{eq}***
		**$FA_{0.89}en_{0.11}Sn_{0.94}Br_3$**			
Sn	5000	5000	5000	0.9441	73(1)
Br	0	5000	5000	1	161(2)
C	0	0	0	0.8882	190(40)
N	2407(4)	0	0	0.2931	330(150)
		**$FA_{0.80}en_{0.20}Sn_{0.90}Br_3$**			
Sn	5000	5000	5000	0.9024	78(1)
Br	0	5000	5000	1	162(2)
C	0	0	0	0.8064	190(20)
N	2403(3)	0	0	0.2656	300(100)
		**$FA_{0.72}en_{0.28}Sn_{0.86}Br_3$**			
Sn	5000	5000	5000	0.8621	79(1)
Br	0	5000	5000	1	163(2)
C	0	0	0	0.7243	190(60)
N	2402(5)	0	0	0.239	300(140)
		**$FA_{0.67}en_{0.33}Sn_{0.84}Br_3$**			
Sn	5000	5000	5000	0.8352	86(2)
Br	0	5000	5000	1	170(2)
C	0	0	0	0.672	260(110)
N	2402(5)	0	0	0.2208	1000(200)

*U_{eq} is defined as one third of the trace of the orthogonalized U_{ij} tensor.

Table S8: Calculated bond lengths (Å) for the $FA_{1\text{-}x}en_xPb_{\eta\text{-}y}Sn_yBr_3$ (η = 1-0.5x) family of perovskites at 295K with estimated standard deviations in parenthesis

Compound	Bond Label	Distance (Å)
	χ_{Sn}= **0.2**	
$FAPb_{0.84}Sn_{0.16}Br_3$	Pb/Sn – Br	2.9789(6)
$FA_{0.89}en_{0.11}Pb_{0.76}Sn_{0.19}Br_3$	Pb/Sn – Br	3.0101(6)
$FA_{0.76}en_{0.24}Pb_{0.69}Sn_{0.18}Br_3$	Pb/Sn – Br	3.0066(4)
$FA_{0.65}en_{0.35}Pb_{0.65}Sn_{0.18}Br_3$	Pb/Sn – Br	3.0189(7)
$FA_{0.55}en_{0.45}Pb_{0.61}Sn_{0.17}Br_3$	Pb/Sn – Br	3.0283(6)
	χ_{Sn}= **0.5**	
$FAPb_{0.50}Sn_{0.50}Br_3$	Pb/Sn – Br	2.9808(9)
$FA_{0.88}en_{0.12}Pb_{0.42}Sn_{0.52}Br_3$	Pb/Sn – Br	3.0038(7)
$FA_{0.81}en_{0.19}Pb_{0.43}Sn_{0.47}Br_3$	Pb/Sn – Br	3.0118(7)
$FA_{0.69}en_{0.31}Pb_{0.42}Sn_{0.43}Br_3$	Pb/Sn – Br	3.0206(3)
$FA_{0.64}en_{0.36}Pb_{0.42}Sn_{0.40}Br_3$	Pb/Sn – Br	3.016(1)
	χ_{Sn}= **0.75**	
$FAPb_{0.24}Sn_{0.76}Br_3$	Pb/Sn – Br	2.980(1)
$FA_{0.91}en_{0.09}Pb_{0.24}Sn_{0.72}Br_3$	Pb/Sn – Br	2.9985(8)
$FA_{0.77}en_{0.23}Pb_{0.22}Sn_{0.66}Br_3$	Pb/Sn – Br	3.0106(7)
$FA_{0.72}en_{0.28}Pb_{0.22}Sn_{0.64}Br_3$	Pb/Sn – Br	3.0206(5)
$FA_{0.66}en_{0.34}Pb_{0.22}Sn_{0.61}Br_3$	Pb/Sn – Br	3.0322(7)
	χ_{Sn}= **1**	
$FA_{0.89}en_{0.11}Sn_{0.94}Br_3$	Pb/Sn – Br	3.0113(7)
$FA_{0.80}en_{0.20}Sn_{0.90}Br_3$	Pb/Sn – Br	3.0162(7)
$FA_{0.72}en_{0.28}Sn_{0.86}Br_3$	Pb/Sn – Br	3.018(1)
$FA_{0.67}en_{0.33}Sn_{0.84}Br_3$	Pb/Sn – Br	3.019(1)

Table S9: Quantitative ^{1}H NMR analysis for the $FA_{1-x}en_xPb_{\eta-y}Sn_yBr_3$ (η = 1-0.5x) family of perovskites.

Loadings		Quantitative Info		Integrations						Calculated Values		
χ_{Sn}	en load (μl)	Maleic Acid (M)	NMR Sample Mass (mg)	FA exo −N−H	FA endo −N−H	en −NH3	FA −CH−	Maleic −$(CH)_2$−	en −$(CH_2)_2$−	FA mmol	en mmol	en/FA ratio
0.2	0	0.11114	45.9	3.4194	3.4055	0	1.6586	2	0	0.0939	0.0000	0.0000
	20	0.11114	49.4	3.4074	3.4333	0.8732	1.7114	2	0.7552	0.0951	0.0105	0.1104
	50	0.11114	52.1	3.3064	3.4051	2.0917	1.7585	2	1.6703	0.0947	0.0232	0.2449
	75	0.11114	50.2	2.9771	3.0041	2.7672	1.5832	2	2.1333	0.0847	0.0296	0.3498
	100	0.11114	59.6	2.6208	2.6897	2.89	1.4524	2	2.4682	0.0913	0.0411	0.4507
0.5	0	0.11114	51.8	4.2294	4.1992	0	2.0328	2	0	0.1157	0.0000	0.0000
	20	0.11114	50.4	3.9282	3.9332	1.0641	1.9522	2	0.9452	0.1090	0.0131	0.1205
	50	0.11114	42.9	3.195	3.19	1.5119	1.6159	2	1.2385	0.0891	0.0172	0.1932
	75	0.11114	52.0	3.3326	3.3696	2.7298	1.7442	2	2.0791	0.0944	0.0289	0.3060
	100	0.11114	53.7	3.3307	3.3398	3.1409	1.6948	2	2.3874	0.0932	0.0332	0.3560
0.75	0	0.11114	53.9	4.9518	5.051	0	2.423	2	0	0.1375	0.0000	0.0000
	20	0.11114	53.0	4.5502	4.5112	0.9019	2.1806	2	0.8348	0.1243	0.0116	0.0933
	50	0.11114	49.5	3.4759	3.5289	1.949	1.7997	2	1.6387	0.0982	0.0228	0.2318
	75	0.11114	56.1	4.112	4.1152	2.8426	2.0844	2	2.2771	0.1148	0.0316	0.2755
	100	0.11114	51.3	3.479	3.4926	3.1095	1.8087	2	2.3776	0.0981	0.0330	0.3368
1	0	0.09425	44.6	5.0077	4.9163	0	2.3848	2	0	0.1154	0.0000	0.0000
	20	0.09425	55.7	6.0572	5.9662	1.534	2.9435	2	1.3345	0.1407	0.0157	0.1118
	50	0.09425	58.8	5.9428	5.9403	2.9861	2.9592	2	2.3231	0.1398	0.0274	0.1958
	75	0.09425	45.8	4.3411	4.3647	2.9697	2.2231	2	2.4173	0.1033	0.0285	0.2757
	100	0.09425	47.9	4.3411	4.3114	3.6311	2.1864	2	2.8653	0.1023	0.0338	0.3300

Table S10: Quantitative ICP-OES analysis for the $FA_{1-x}en_xPb_{\eta-y}Sn_yBr_3$ (η = 1-0.5x) family of perovskites

χ_{Sn}	x (en^{2+})	Sn (ppm)	Pb (ppm)	Sn mM	Pb mM	Sn/Pb	Pb/Sn
0.2	0	83.41	764.5	0.7027	3.691	0.1904	5.253
0.2	0.11	54.61	384.6	0.4599	1.855	0.2479	4.035
0.2	0.24	53.96	353.2	0.4546	1.703	0.2669	3.747
0.2	0.35	50.63	323.9	0.4266	1.563	0.2730	3.663
0.2	0.45	36.05	227.6	0.3036	1.098	0.2765	3.617
0.5	0	136.2	236.7	1.148	1.142	1.005	0.9952
0.5	0.12	203.5	283.7	1.714	1.370	1.251	0.7995
0.5	0.19	0.7393	0.6692	0.7393	0.6692	1.105	0.9052
0.5	0.31	1.048	1.040	1.048	1.039	1.008	0.9919
0.5	0.36	1.050	1.110	1.050	1.110	0.9456	1.057
0.75	0	431.7	236.7	3.637	1.142	3.184	0.3141
0.75	0.09	287.8	166.1	2.423	0.8014	3.024	0.3307
0.75	0.23	200.4	116.2	1.689	0.5608	3.012	0.3320
0.75	0.28	268.8	165.2	2.263	0.7974	2.838	0.3524
0.75	0.34	215.6	135.5	1.815	0.6540	2.776	0.3602
1	0	182.7	0.00	1.539	0	———	———
1	0.11	250.4	0.00	2.109	0	———	———
1	0.20	393.4	0.00	3.314	0	———	———
1	0.28	247.5	0.00	2.085	0	———	———
1	0.33	341.0	0.00	2.871	0	———	———

Table S11. XPS peak statistics for Sn $3d_{5/2}$ spectrum

	peak max $3d_{5/2}$	$3d_{5/2}$ FWHM (eV)	% 2+	% 4+
Standard 2+	486.38	1.1	89%	11%
$FASnBr_3$ (χ_{Sn} = 1, x = 0)	486.74	1.2	93%	7%
$FA_{0.67}en_{0.33}Sn_{0.84}Br_3$ (χ_{Sn} = 1, x = 0.33)	486.71	1	96%	4%
$FAPb_{0.5}Sn_{0.5}Br_3$ (χ_{Sn} = 0.5, x = 0)	487.01	1.4	88%	12%
$FA_{0.64}en_{0.36}Pb_{0.42}Sn_{0.40}Br_3$ (χ_{Sn} = 0.5, x = 0.36)	486.60	1.1	96%	4%
Standard 4+	487.60	1.4	-	-

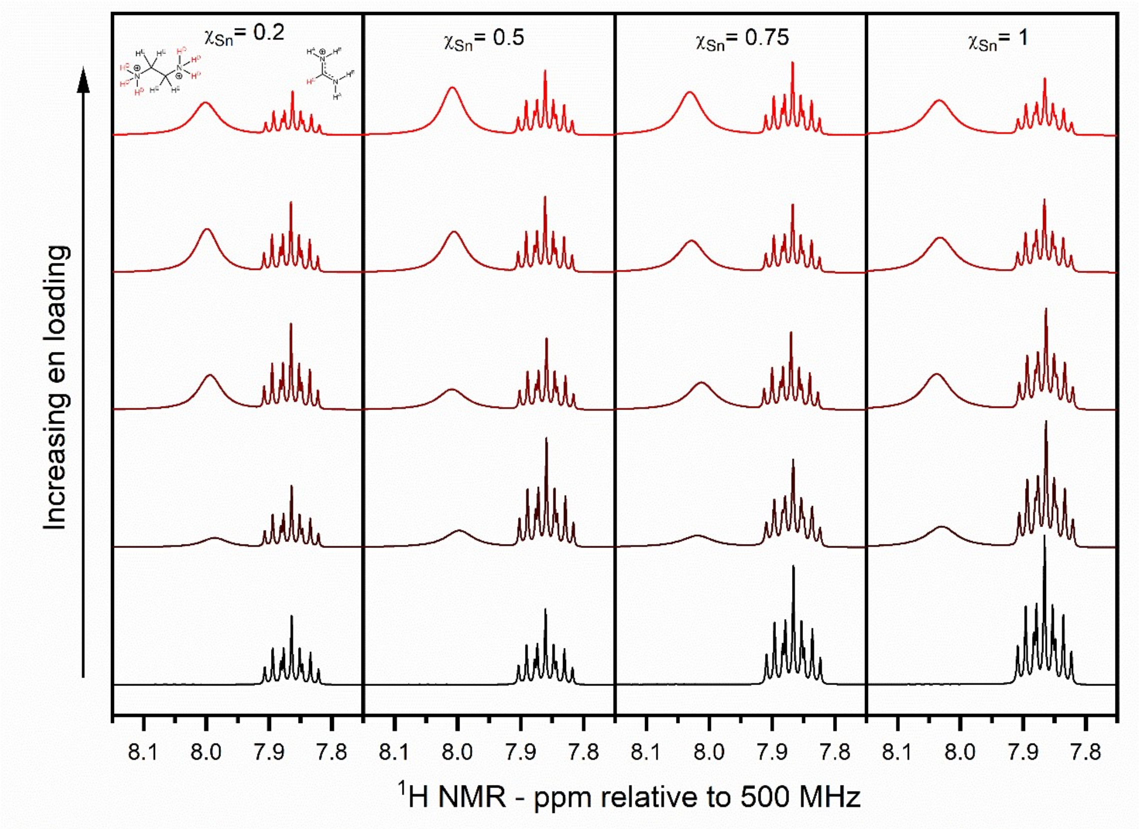

Figure S1: ^{1}H NMR Spectra for the compounds in the $FA_{1-x}en_xPb_{\eta-y}Sn_yBr_3$ (η = 1-0.5x) family of perovskites shown in the range of 8.15 to 7.75 ppm.

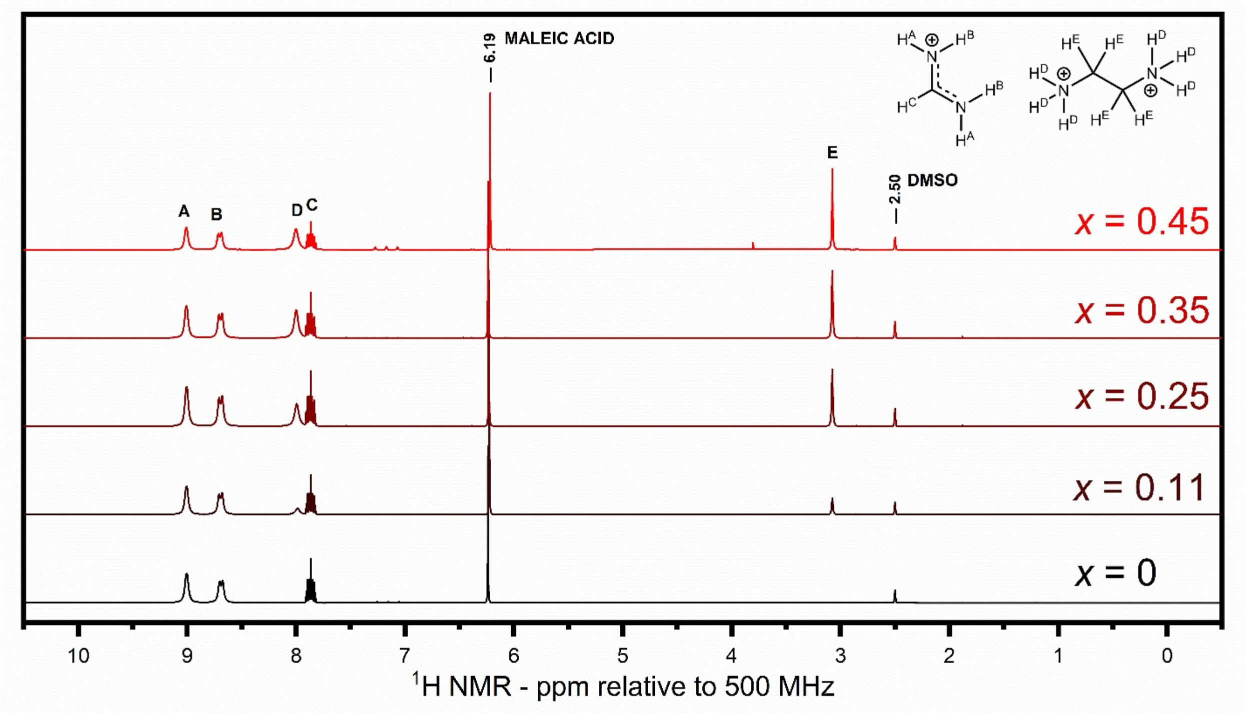

Figure S2: ^{1}H NMR spectrum for the ^{1}H NMR Spectra for the compounds in the $FA_{1-x}en_xPb_{\eta-y}Sn_yBr_3$ (η = 1-0.5x) family of perovskites where χ_{Sn}= 0.2.

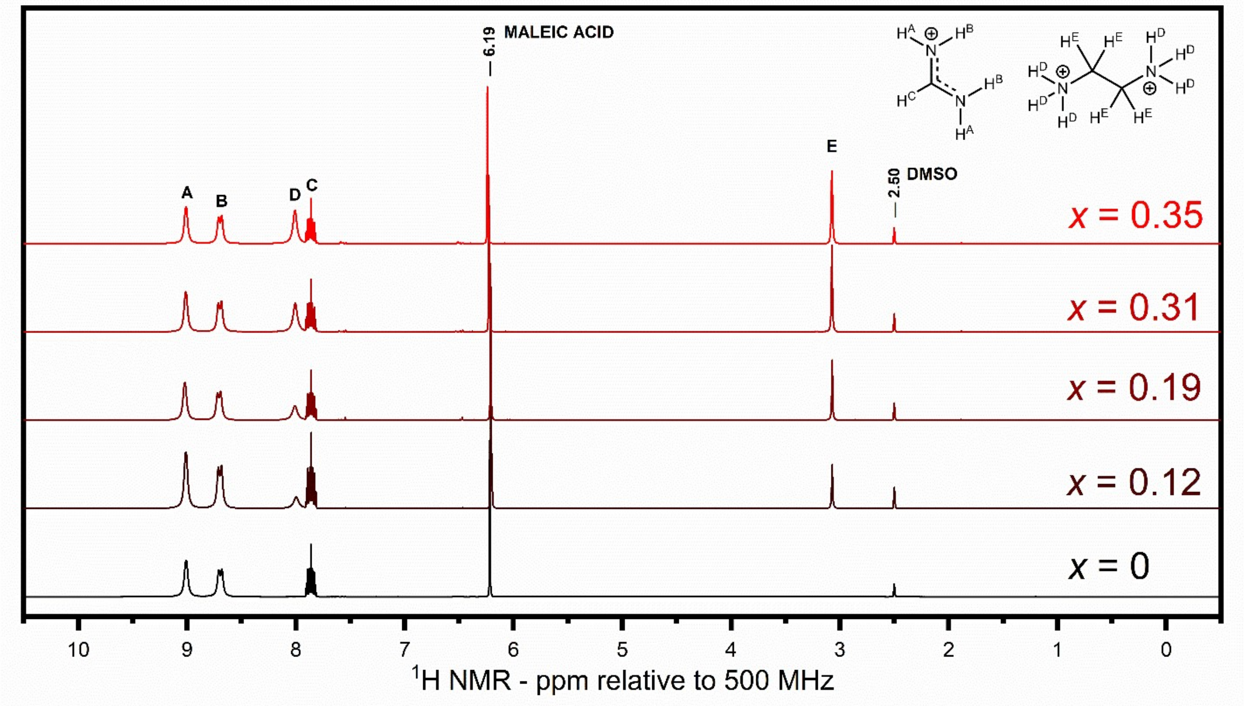

Figure S3: ^{1}H NMR spectrum for the ^{1}H NMR Spectra for the compounds in the $FA_{1\text{-}x}en_xPb_{\eta\text{-}y}Sn_yBr_3$ (η = 1-0.5x) family of perovskites where χ_{Sn}= 0.5.

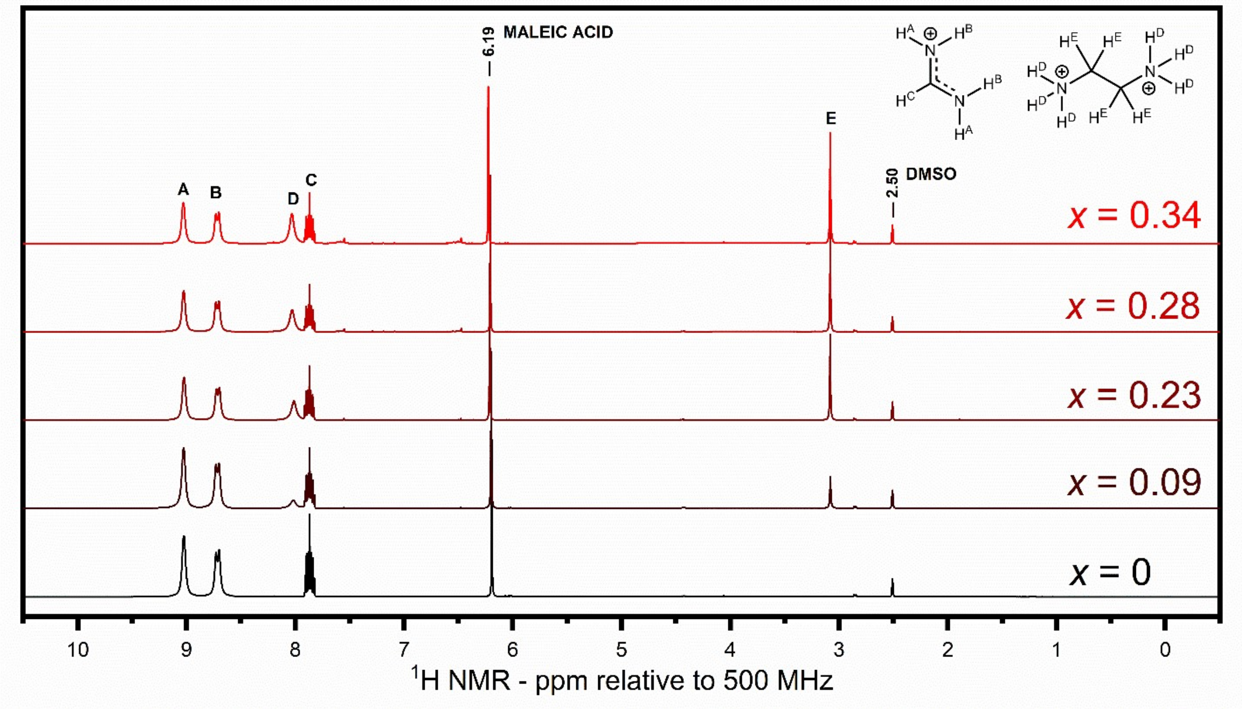

Figure S4: ^{1}H NMR spectrum for the ^{1}H NMR Spectra for the compounds in the $FA_{1\text{-}x}en_xPb_{\eta\text{-}y}Sn_yBr_3$ (η = 1-0.5x) family of perovskites where χ_{Sn}= 0.75.

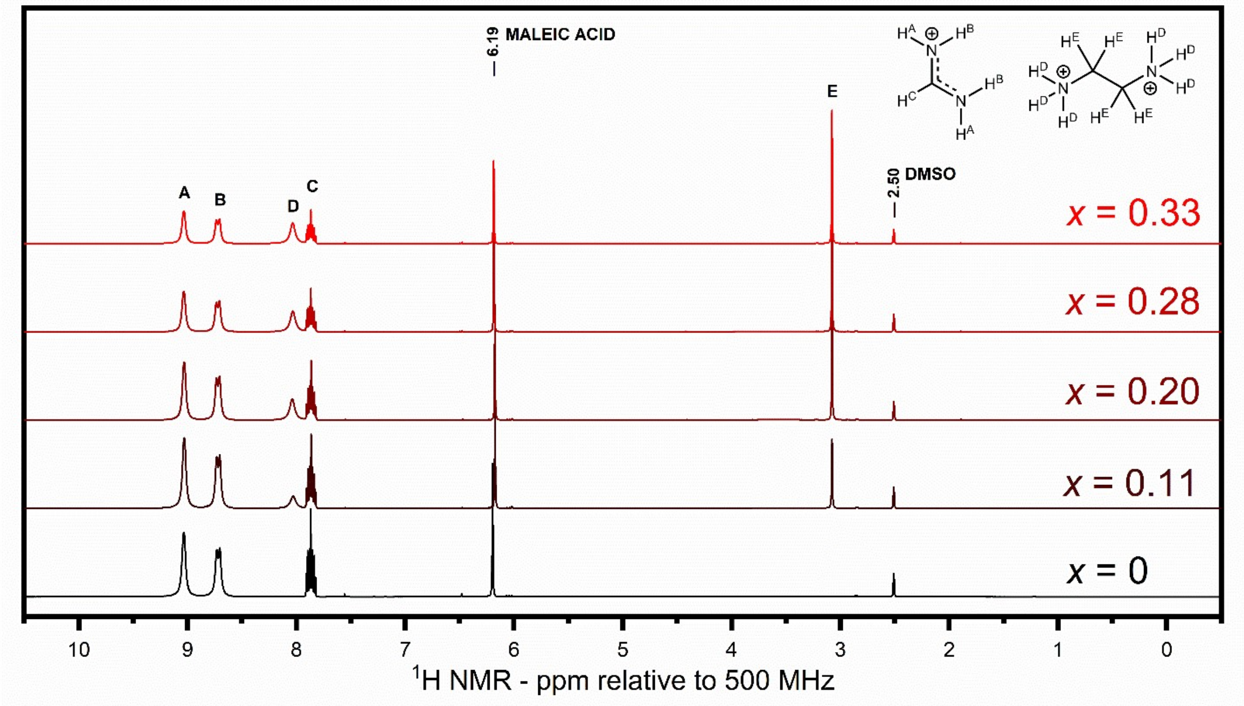

Figure S5: ^{1}H NMR spectrum for the ^{1}H NMR Spectra for the compounds in the $FA_{1-x}en_xPb_{\eta-y}Sn_yBr_3$ (η = 1-0.5x) family of perovskites where χ_{Sn}= 1.

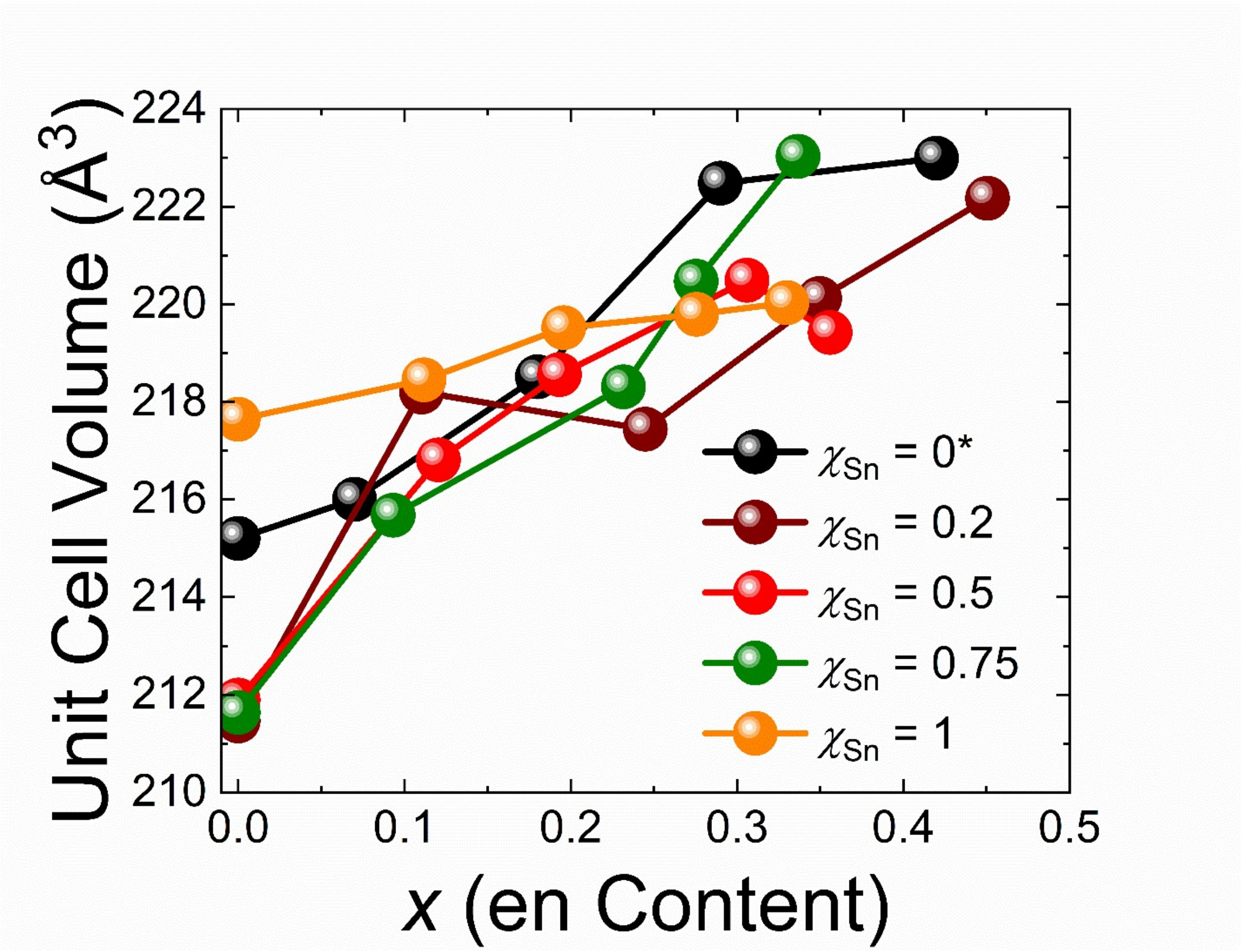

Figure S6: The measured unit cell volume for the $FA_{1\text{-}x}en_xPb_{\eta\text{-}y}Sn_yBr_3$ (η = 1-0.5x) family of perovskites. *See Note S1.

Note S1: The full Pb data (χ_{Sn}= 0) plotted here was obtained from our previous study.[2]

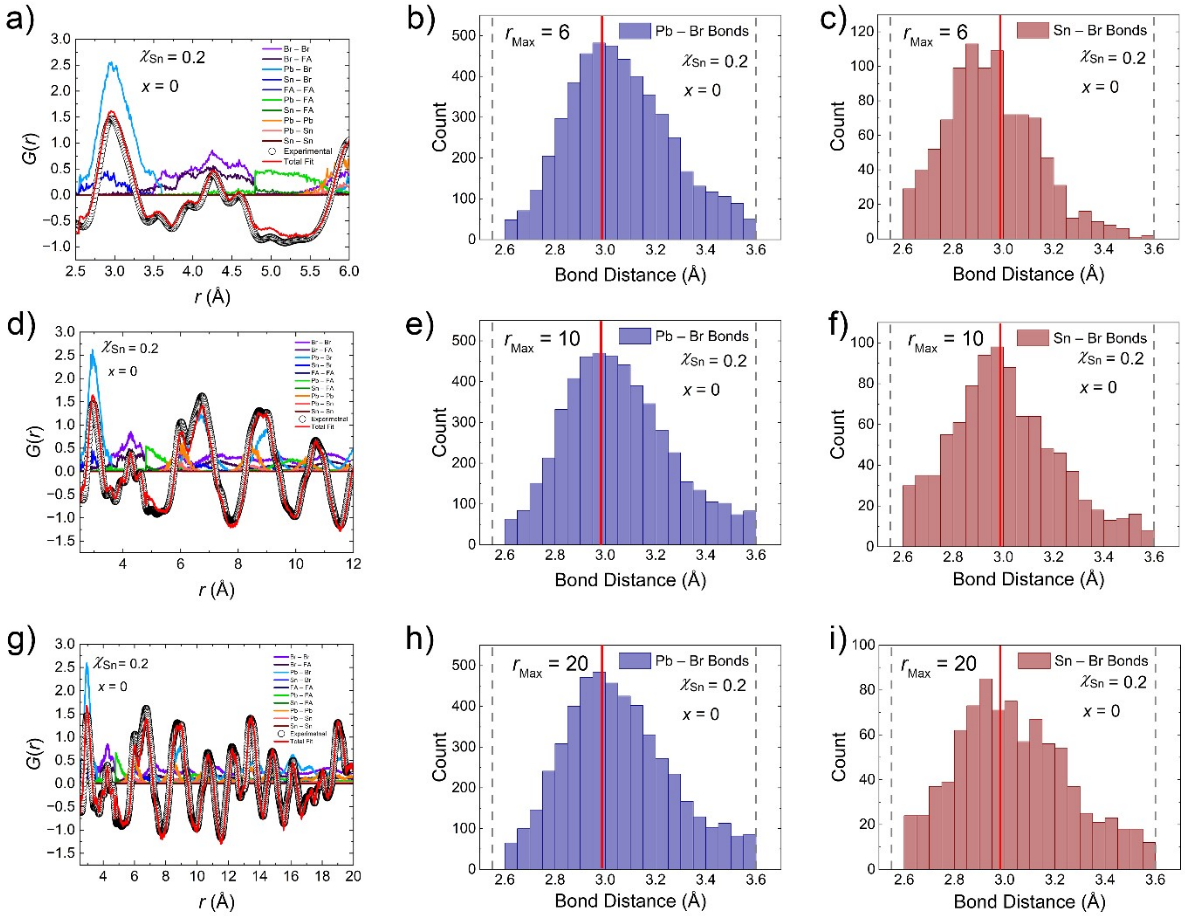

Figure S7: Results from pair distribution function fitting from Reverse Monte Carlo simulations on $FAPb_{0.84}Sn_{0.16}Br_3$ considering 3 different scales of *r* where the experimental data, total fit, and partial radial distribution functions for all atomic pairs are shown (a, d, g). Histograms of the Pb – Br (b, e, h) and Sn – Br (c, f, i) from the generated model are also shown with the average M – X bond distance obtained from single crystal X-ray diffraction experiments shown as a red line.

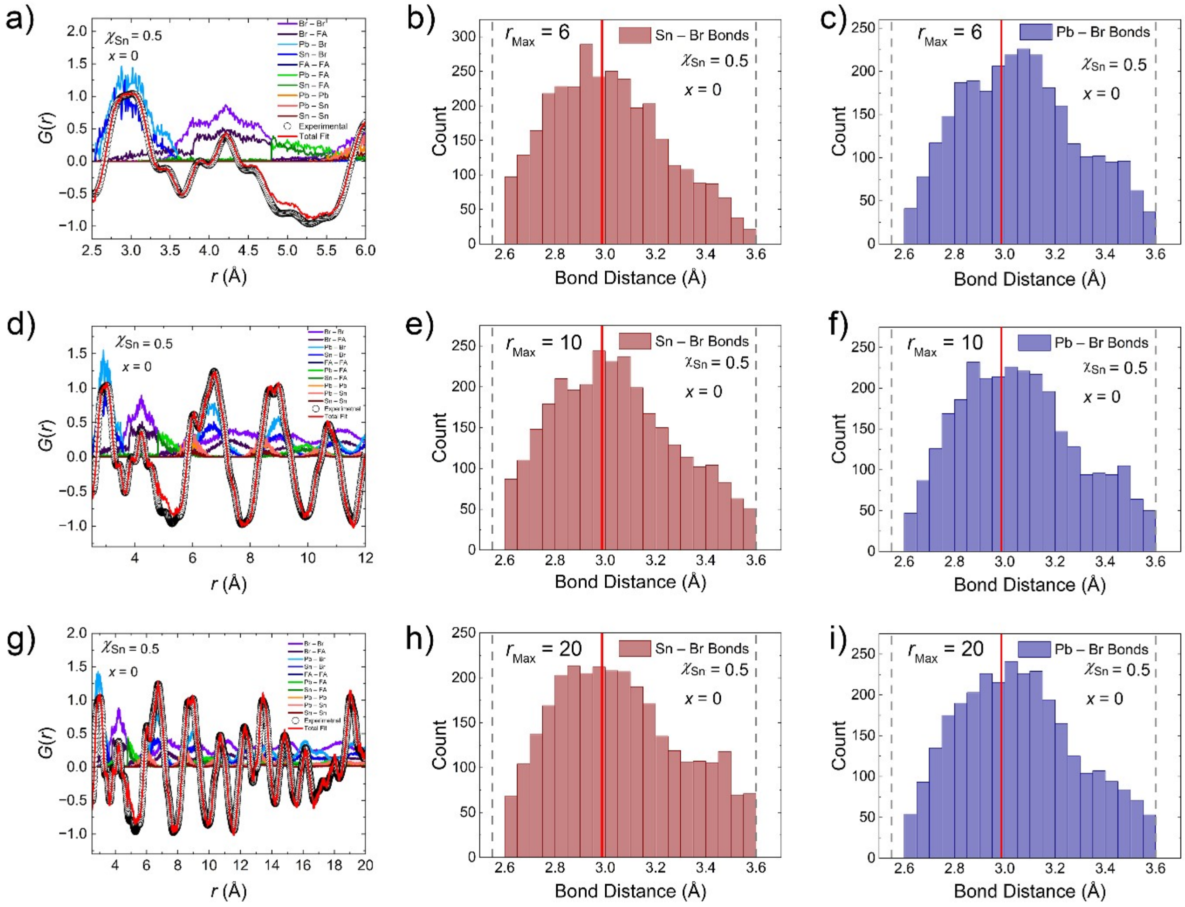

Figure S8: Results from pair distribution function fitting from Reverse Monte Carlo simulations on $FAPb_{0.50}Sn_{0.50}Br_3$ considering 3 different scales of *r* where the experimental data, total fit, and partial radial distribution functions for all atomic pairs are shown (a, d, g). Histograms of the Pb – Br (b, e, h) and Sn – Br (c, f, i) from the generated model are also shown with the average M – X bond distance obtained from single crystal X-ray diffraction experiments shown as a red line.

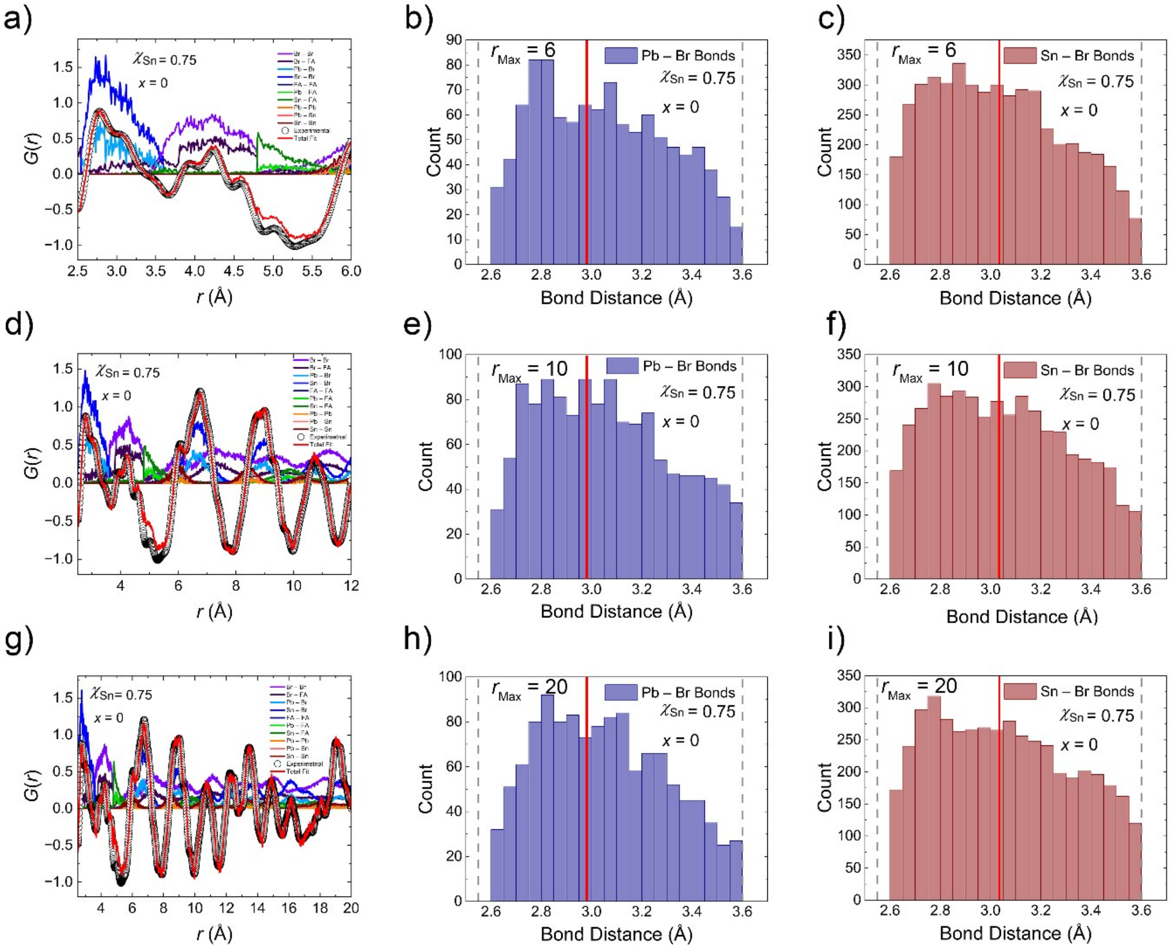

Figure S9: Results from pair distribution function fitting from Reverse Monte Carlo simulations on $FAPb_{0.21}Sn_{0.79}Br_3$ considering 3 different scales of *r* where the experimental data, total fit, and partial radial distribution functions for all atomic pairs are shown (a, d, g). Histograms of the Pb – Br (b, e, h) and Sn – Br (c, f, i) from the generated model are also shown with the average M – X bond distance obtained from single crystal X-ray diffraction experiments shown as a red line.

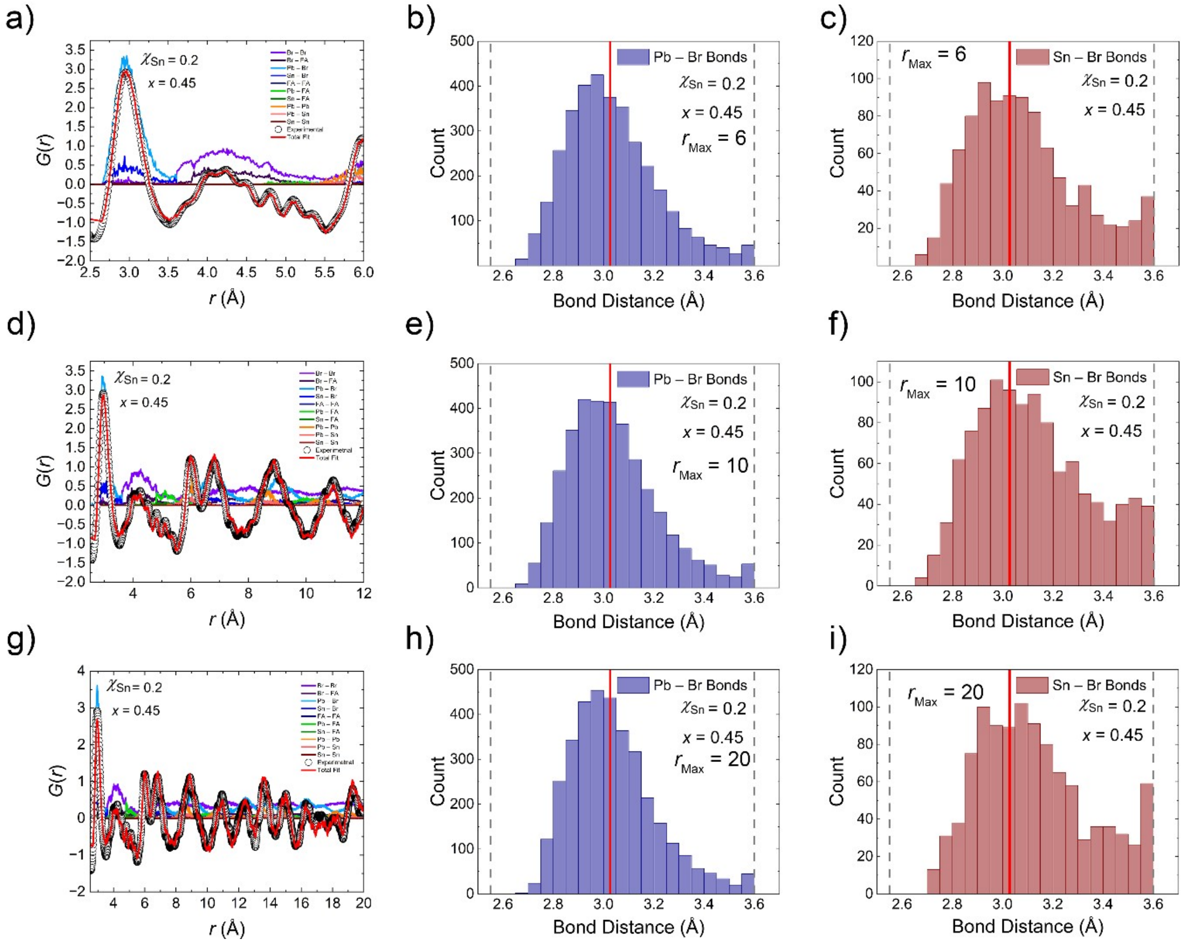

Figure S10: Results from pair distribution function fitting from Reverse Monte Carlo simulations on $FA_{0.55}en_{0.45}Pb_{0.61}Sn_{0.17}Br_3$ considering 3 different scales of *r* where the experimental data, total fit, and partial radial distribution functions for all atomic pairs are shown (a, d, g). Histograms of the Pb – Br (b, e, h) and Sn – Br (c, f, i) from the generated model are also shown with the average M – X bond distance obtained from single crystal X-ray diffraction experiments shown as a red line.

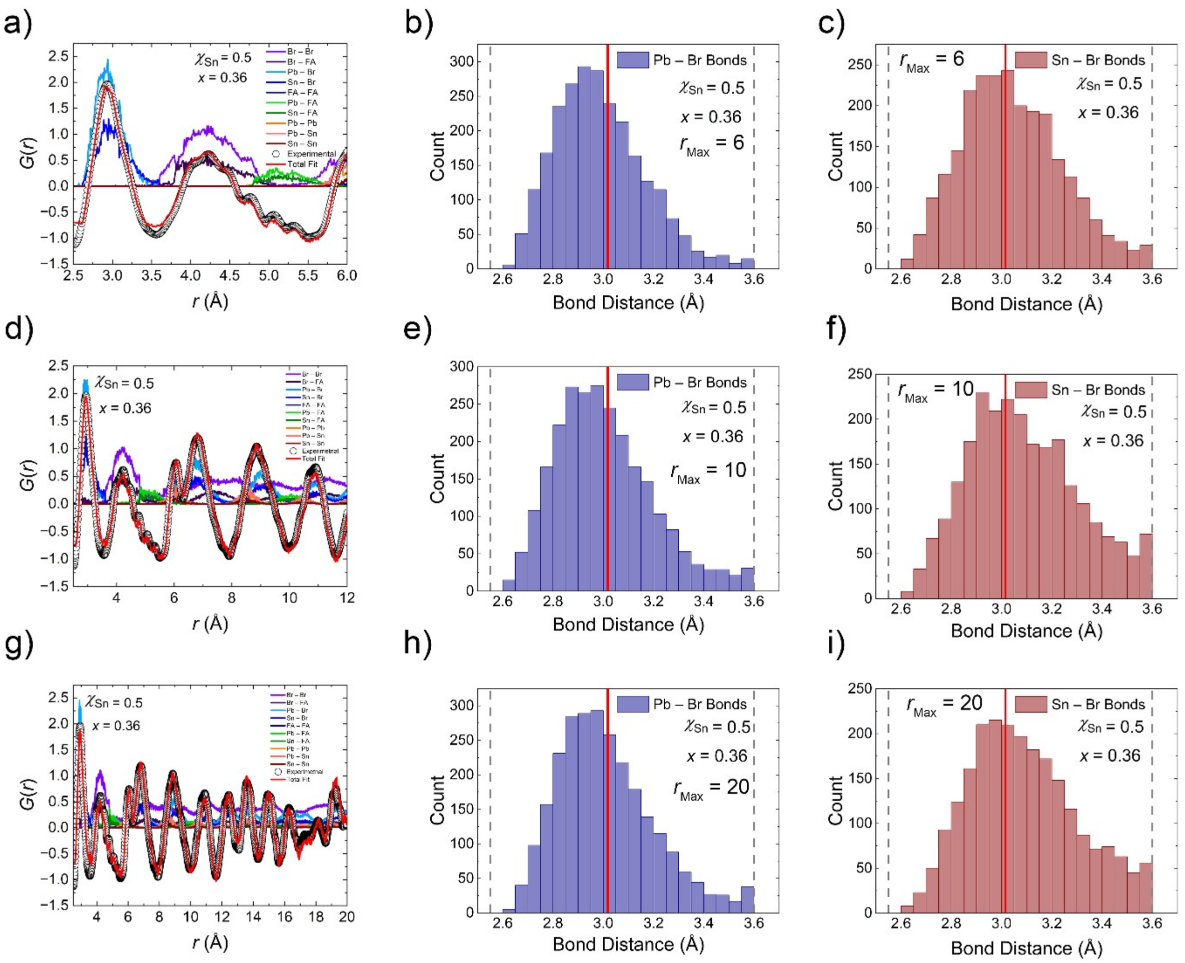

Figure S11: Results from pair distribution function fitting from Reverse Monte Carlo simulations on $FA_{0.64}en_{0.36}Pb_{0.42}Sn_{0.40}Br_3$ considering 3 different scales of *r* where the experimental data, total fit, and partial radial distribution functions for all atomic pairs are shown (a, d, g). Histograms of the Pb – Br (b, e, h) and Sn – Br (c, f, i) from the generated model are also shown with the average M – X bond distance obtained from single crystal X-ray diffraction experiments shown as a red line.

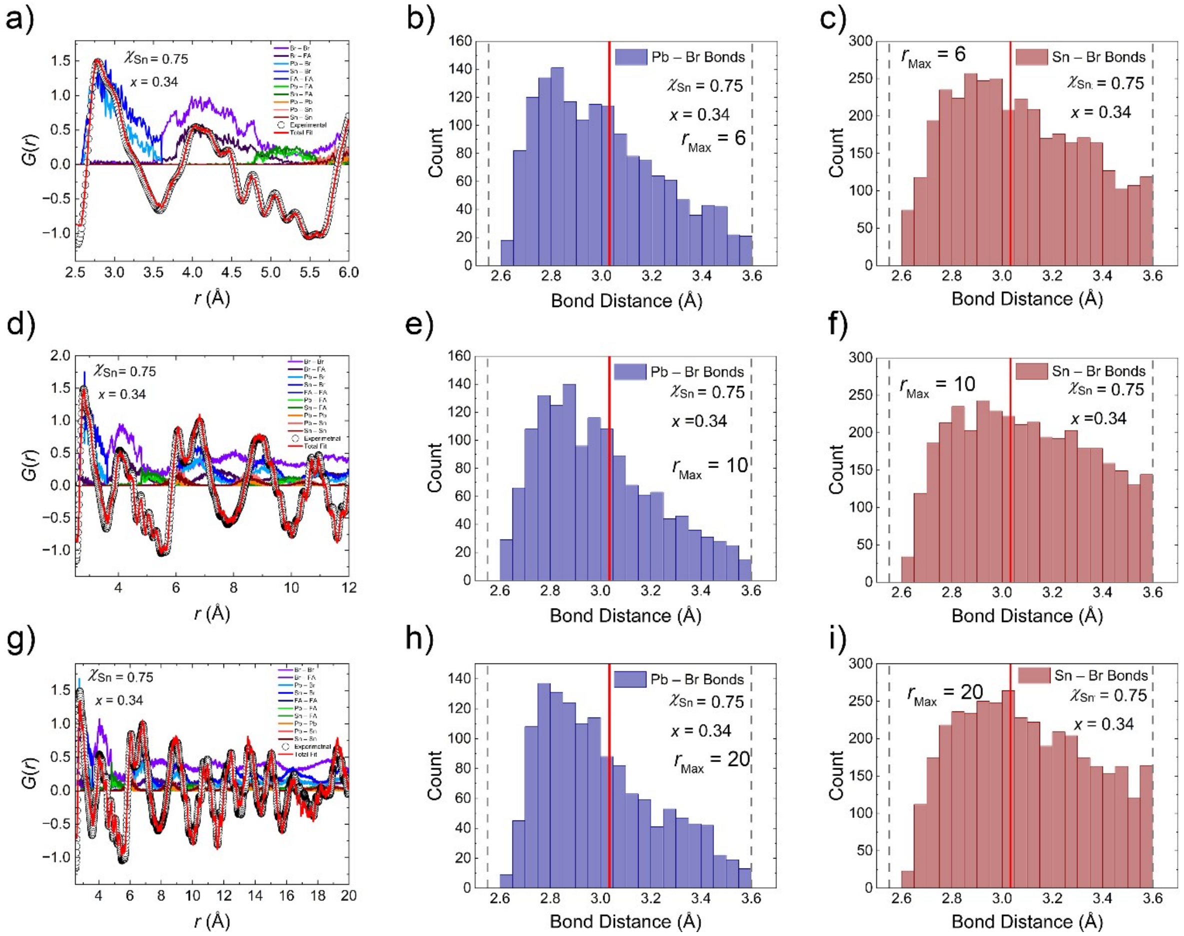

Figure S12: Results from pair distribution function fitting from Reverse Monte Carlo simulations on $FA_{0.66}en_{0.34}Pb_{0.22}Sn_{0.61}Br_3$ considering 3 different scales of *r* where the experimental data, total fit, and partial radial distribution functions for all atomic pairs are shown (a, d, g). Histograms of the Pb – Br (b, e, h) and Sn – Br (c, f, i) from the generated model are also shown with the average M – X bond distance obtained from single crystal X-ray diffraction experiments shown as a red line.

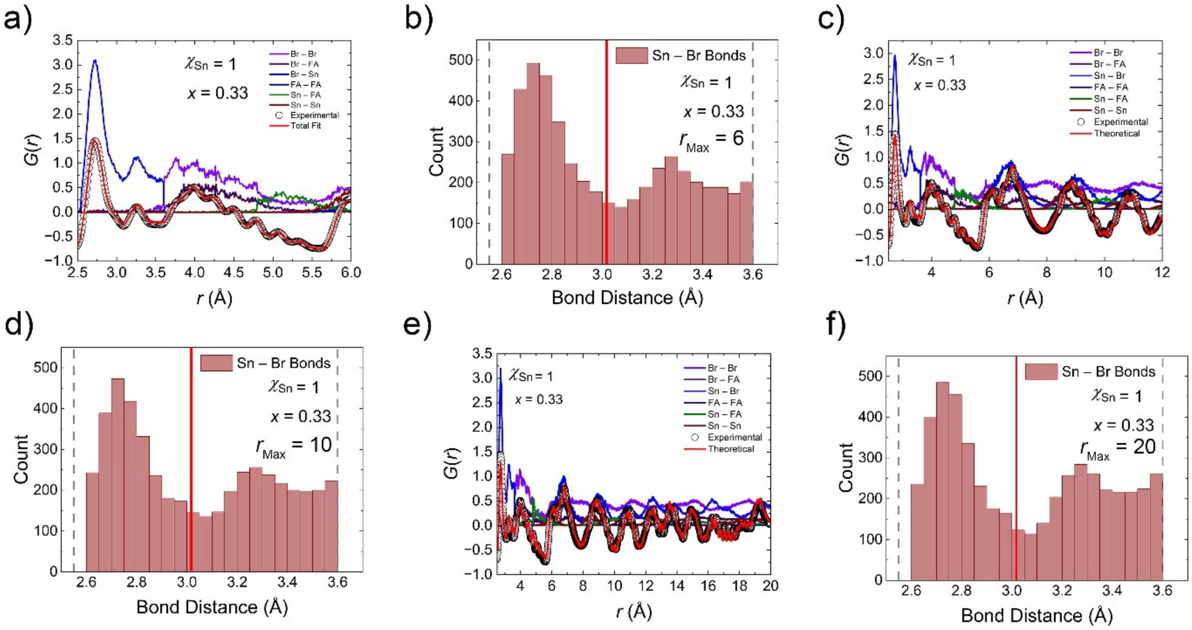

Figure S13: Results from pair distribution function fitting from Reverse Monte Carlo simulations on $FA_{0.67}en_{0.33}Sn_{0.84}Br_3$ considering 3 different scales of *r* where the experimental data, total fit, and partial radial distribution functions for all atomic pairs are shown (a, c, e). Histograms of the Sn – Br (b, d, f) from the generated model are also shown with the average M – X bond distance obtained from single crystal X-ray diffraction experiments shown as a red line.

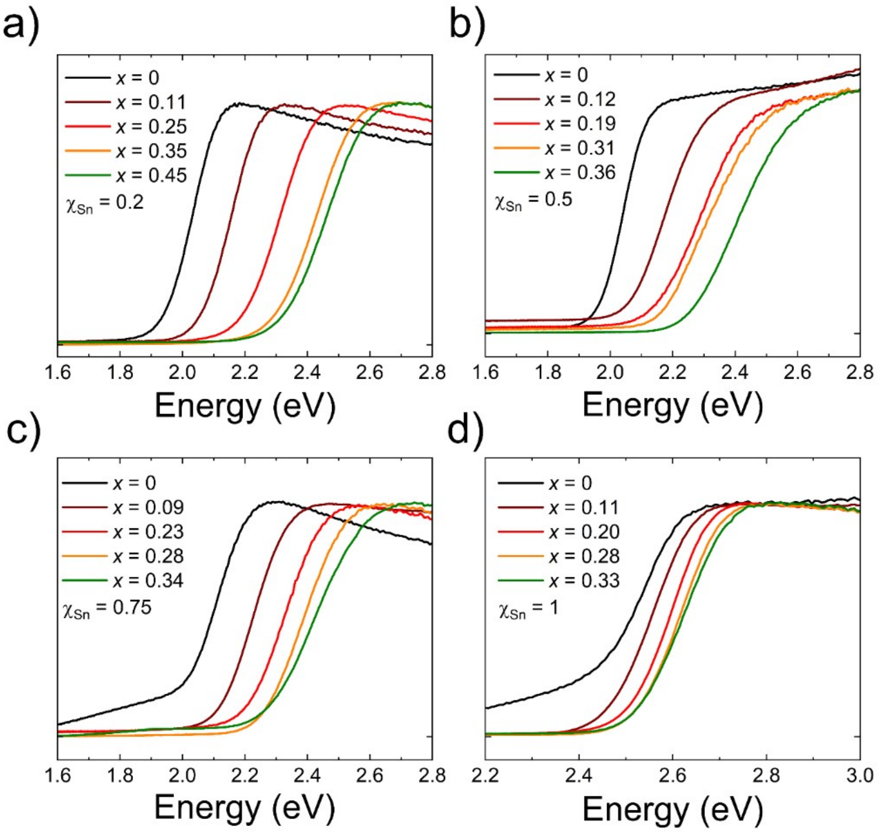

Figure S14: Diffuse reflectance UV-Vis spectra for the $FA_{1-x}en_xPb_{\eta-y}Sn_yBr_3$ samples in four panels where a), b), c), and d) represent compounds with a 0.2, 0.5, 0.75, and 1 experimental Sn mol fraction respectively.

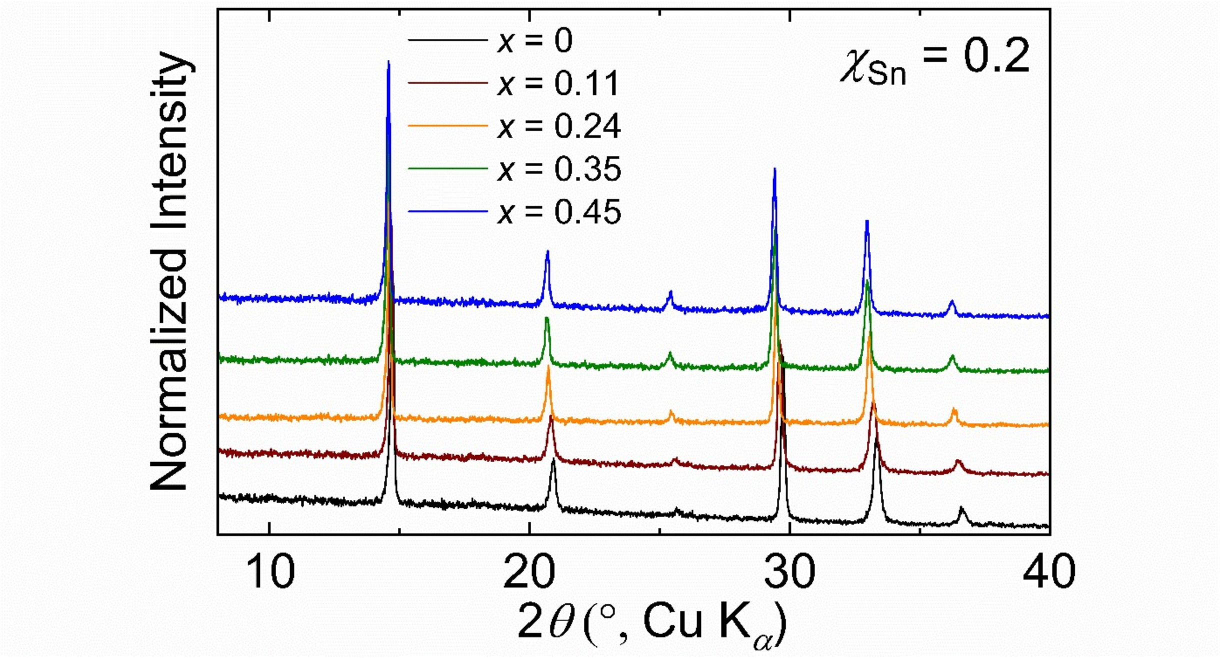

Figure S15: Air-free powder diffraction patterns for the χ_{Sn} = 0.2 compounds with various *en* subsitution fractions.

Note S2: The measurment was conducted in a transmission geometry which can lead to high backgrounds especially at low angles.

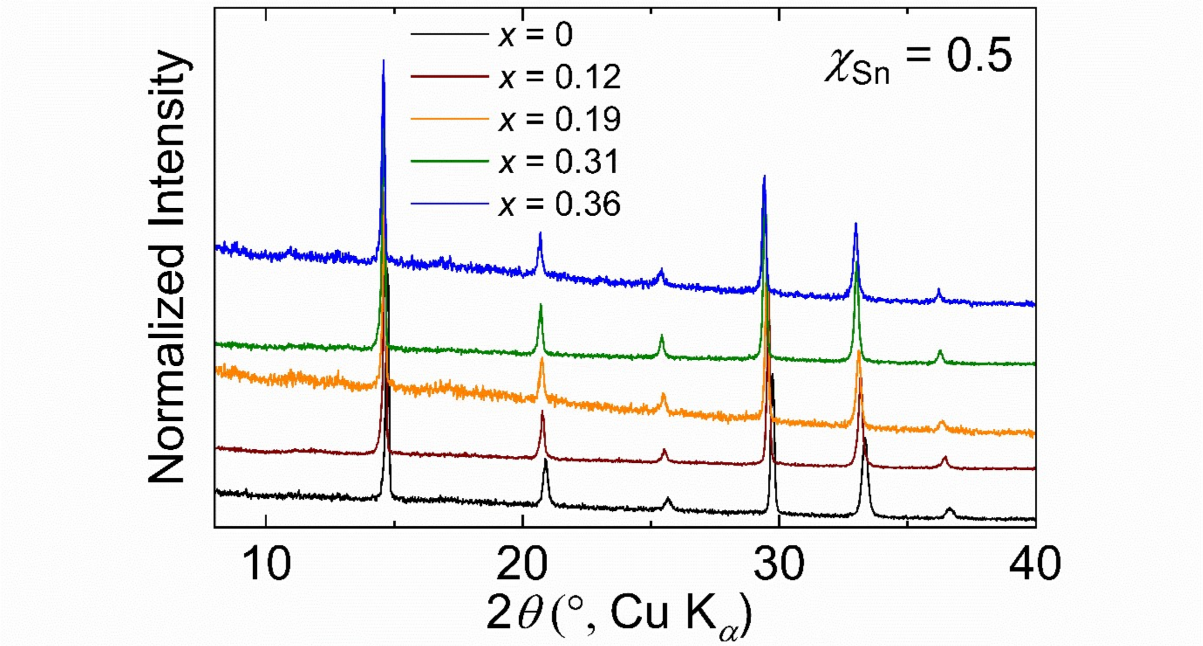

Figure S16: Air-free powder diffraction patterns for the χ_{Sn} = 0.5 compounds with various *en* subsitution fractions.

Note S3: The measurment was conducted in a transmission geometry which can lead to high backgrounds especially at low angles.

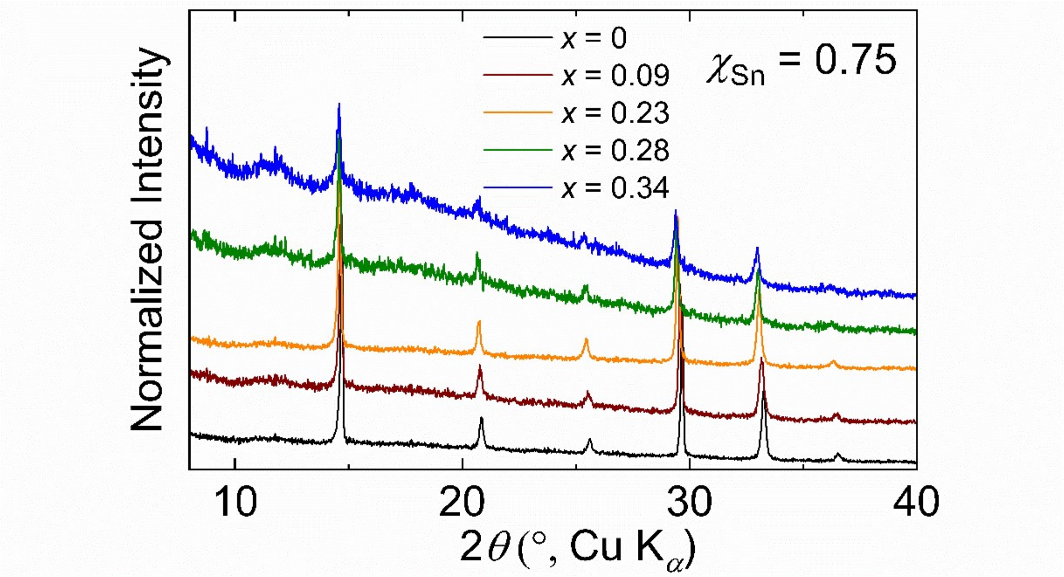

Figure S17: Air-free powder diffraction patterns for the χ_{Sn} = 0.75 compounds with various *en* subsitution fractions.

Note S4: The measurment was conducted in a transmission geometry which can lead to high backgrounds especially at low angles.

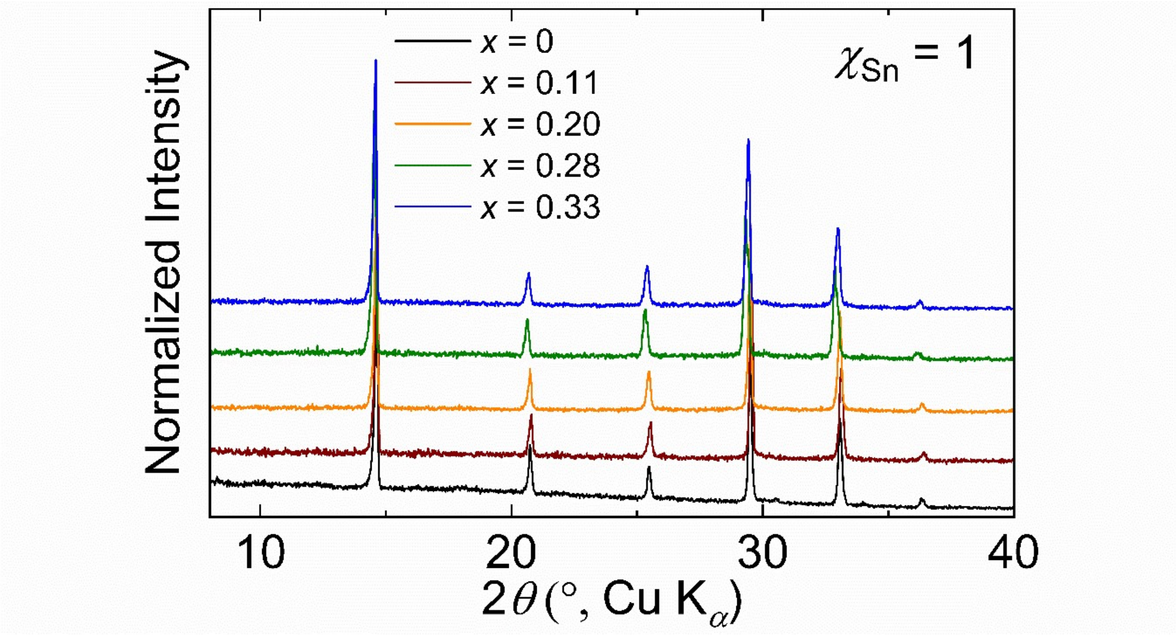

Figure S18: Air-free powder diffraction patterns for the χ_{Sn} = 1 compounds with various *en* subsitution fractions.

Note S5: The measurment was conducted in a transmission geometry which can lead to high backgrounds especially at low angles.

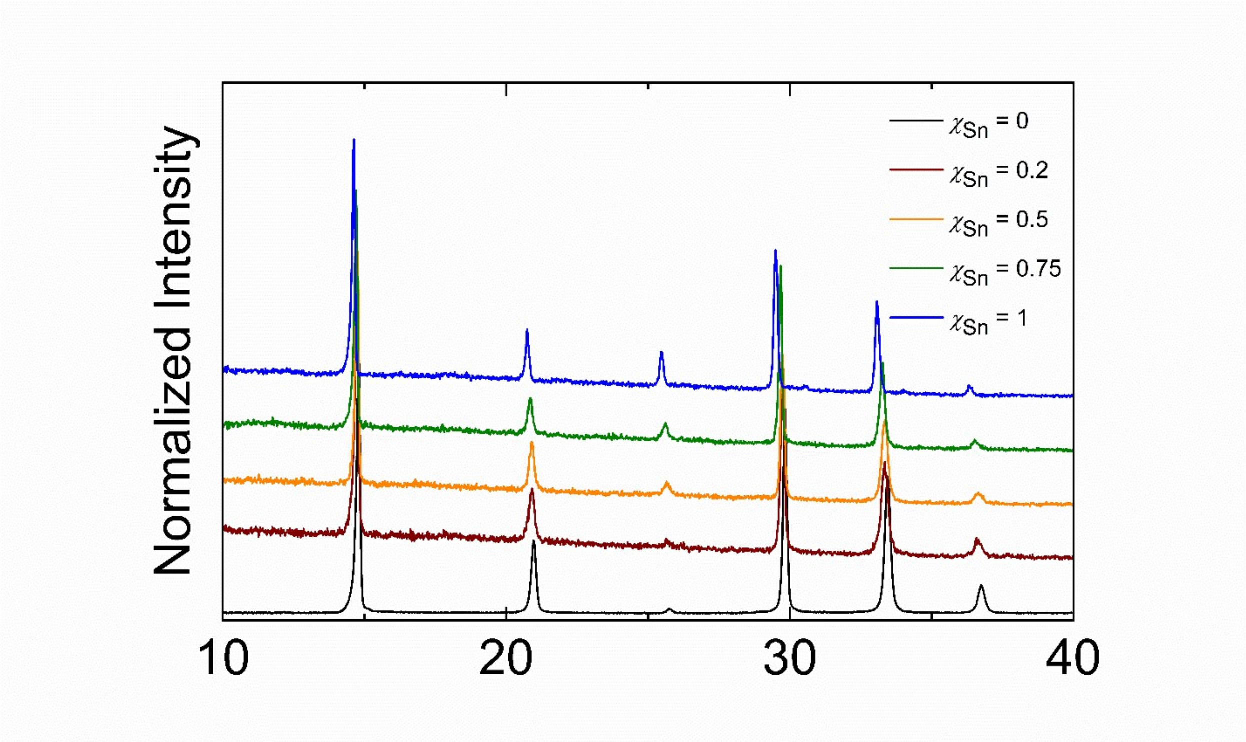

Figure S19: Experimental powder difrraction patterns for the $x = 0$ compounds with varying Sn concentration. *$FAPbBr_3$ ($\chi_{Sn} = 0$) was prepared using the previously published synthetic procedures[2] and was measured in reflection geomery.

Note S6: The measurment was conducted in a transmission geometry (with the exception of $FAPbBr_3$) which can lead to high backgrounds especially at low angles.

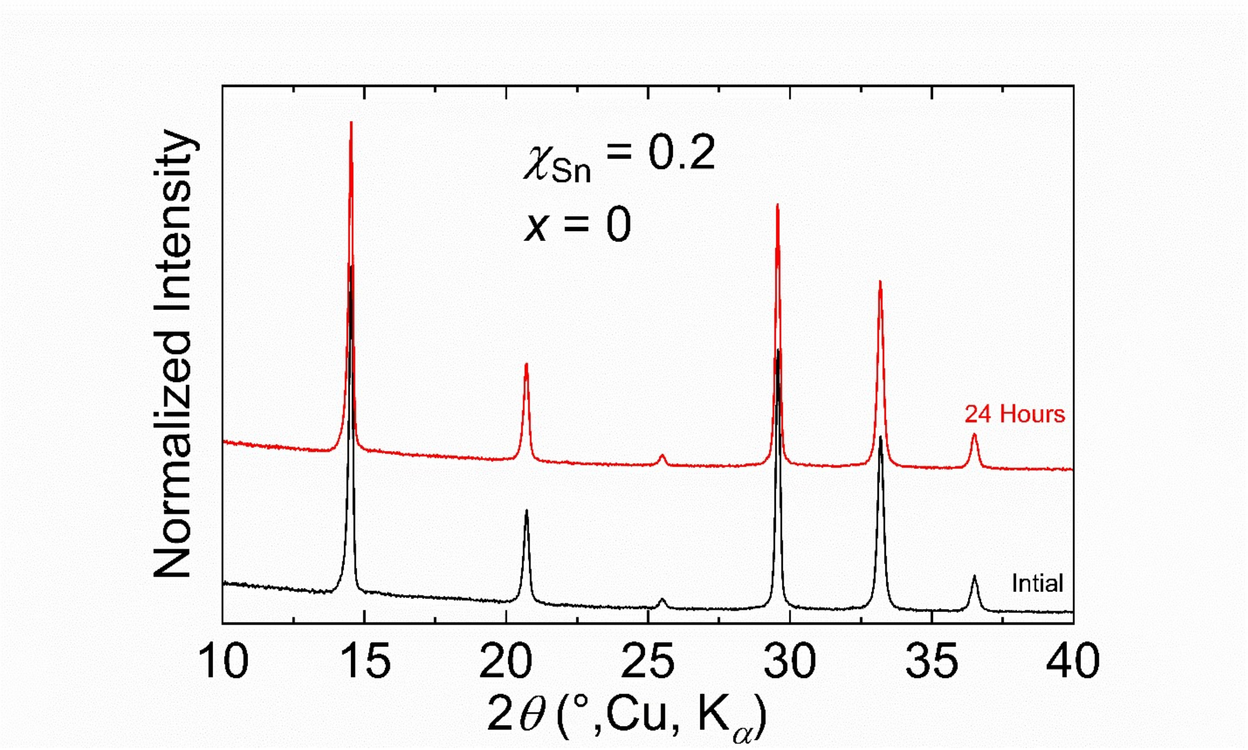

Figure S20: Experimental powder diffraction patterns for the $x = 0$, $\chi_{Sn} = 0.2$ compound upon intial and 24 hours of exposure to air.

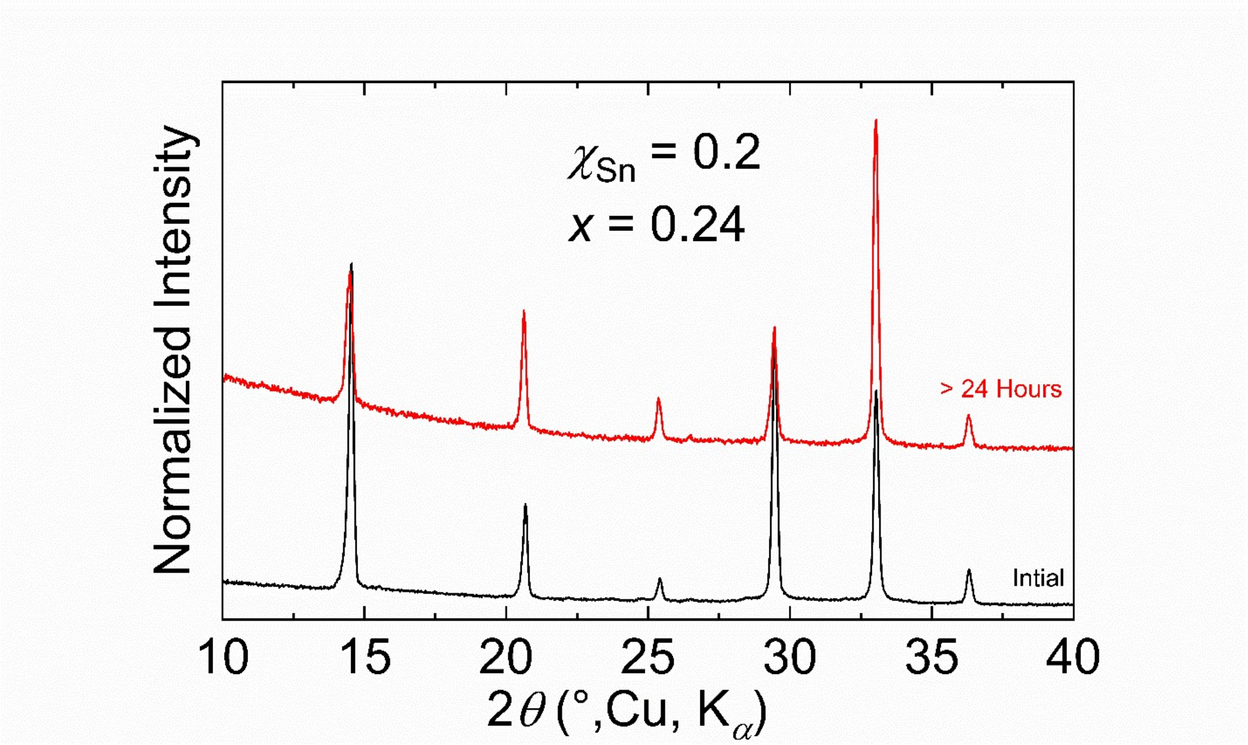

Figure S21: Experimetal powder diffraction patterns for the $x = 0.2$X, $\chi_{Sn} = 0.2$ compound upon intial and 24 hours of exposure to air.

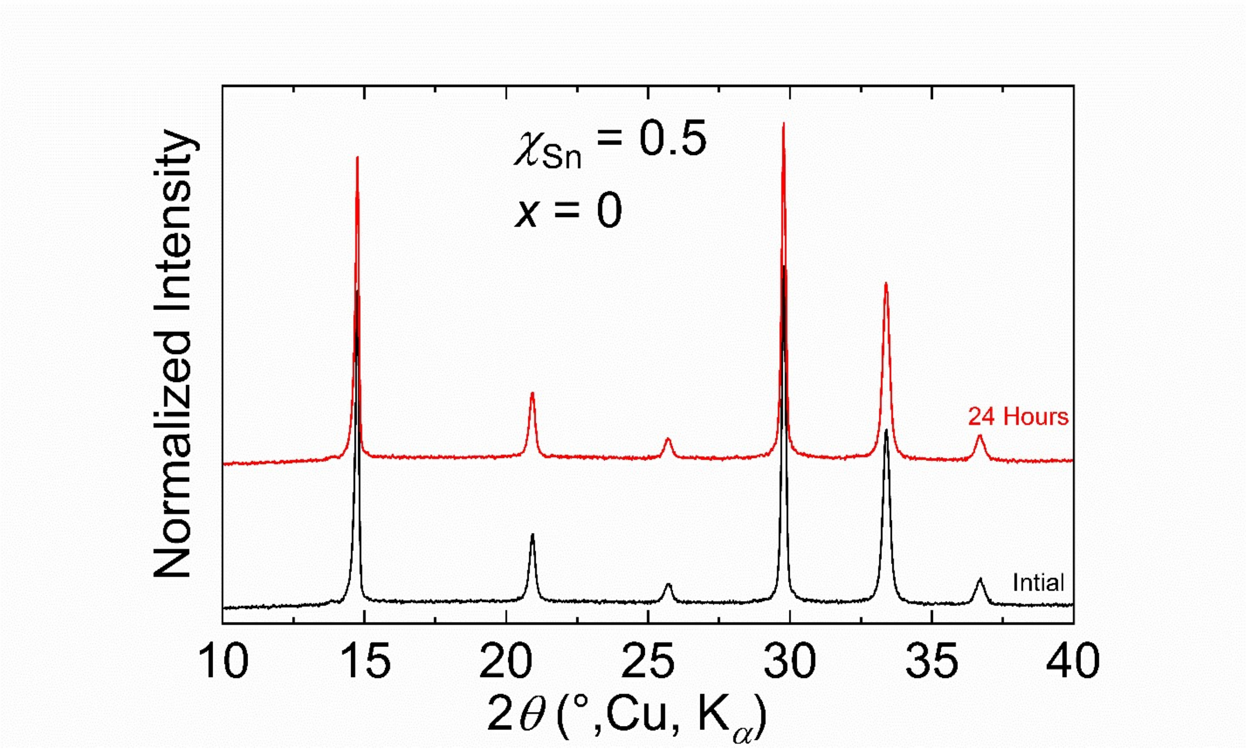

Figure S22: Experimental powder diffraction pattern for the $x = 0$, $\chi_{Sn} = 0.5$ compound upon intial and 24 hours of exposure to air.

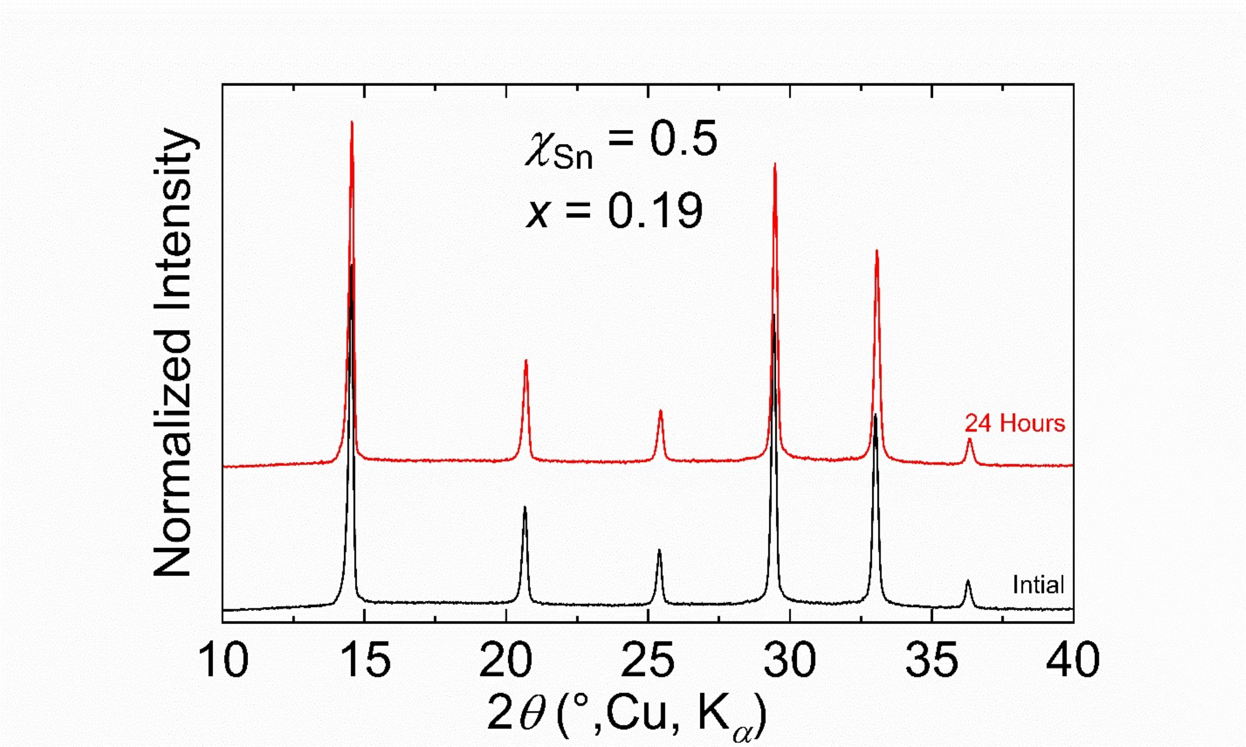

Figure S23: Experimental powder diffraction pattern for the $x = 0.19$, $\chi_{Sn} = 0.5$ compound upon intial and 24 hours of exposure to air.

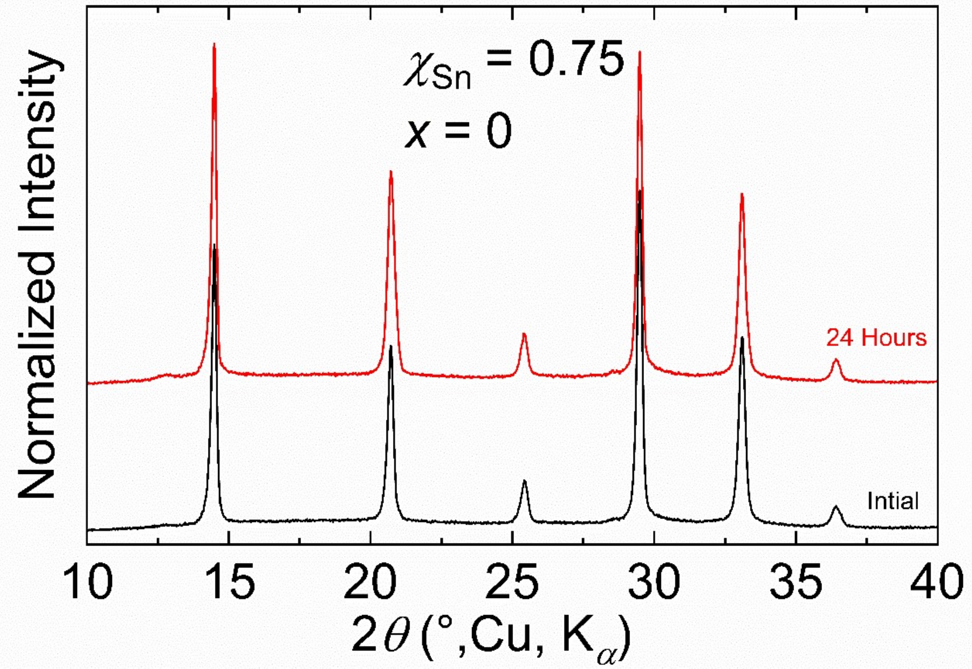

Figure S24: Experimental powder diffraction pattern for the $x = 0$, $\chi_{Sn} = 0.75$ compound upon intial and 24 hours of exposure to air.

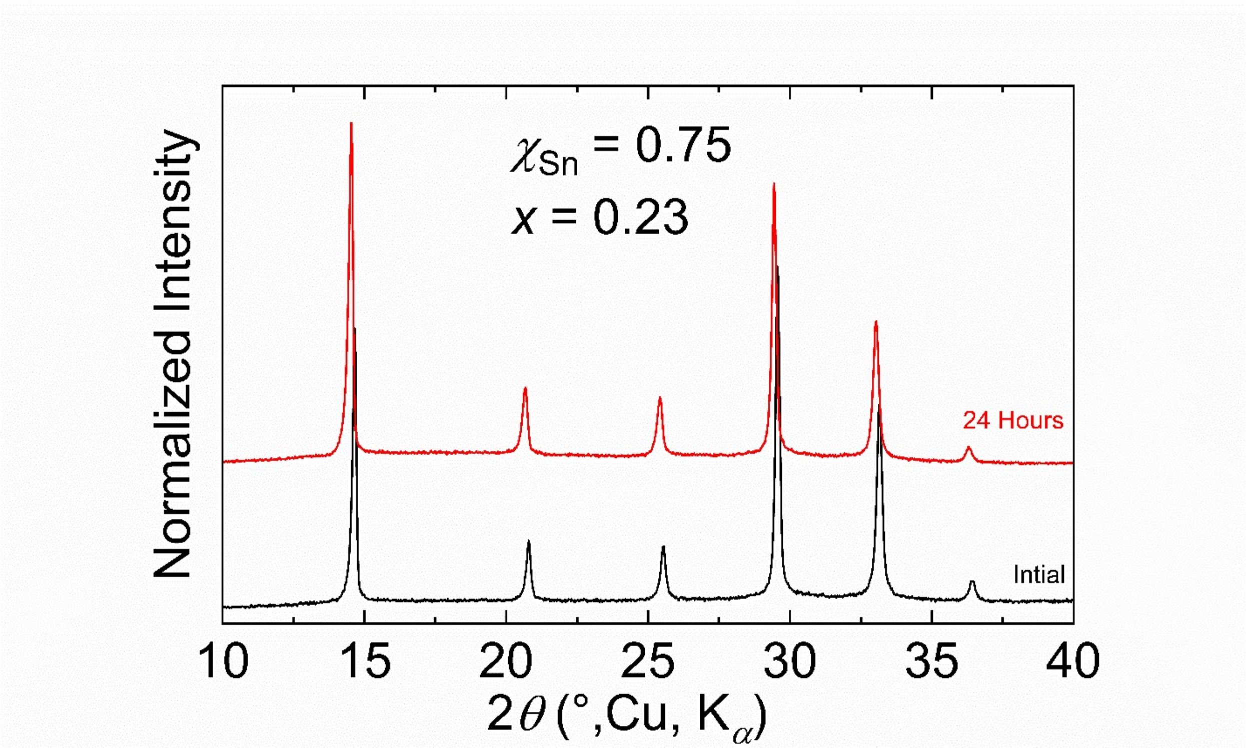

Figure S25: Experimental powder diffraction patterns for the $x = 0.23$, $\chi_{Sn} = 0.75$ compound upon intial and 24 hours of exposure to air.

Note S7: Shifting of peak position is consistent with variablity in sample preparation.

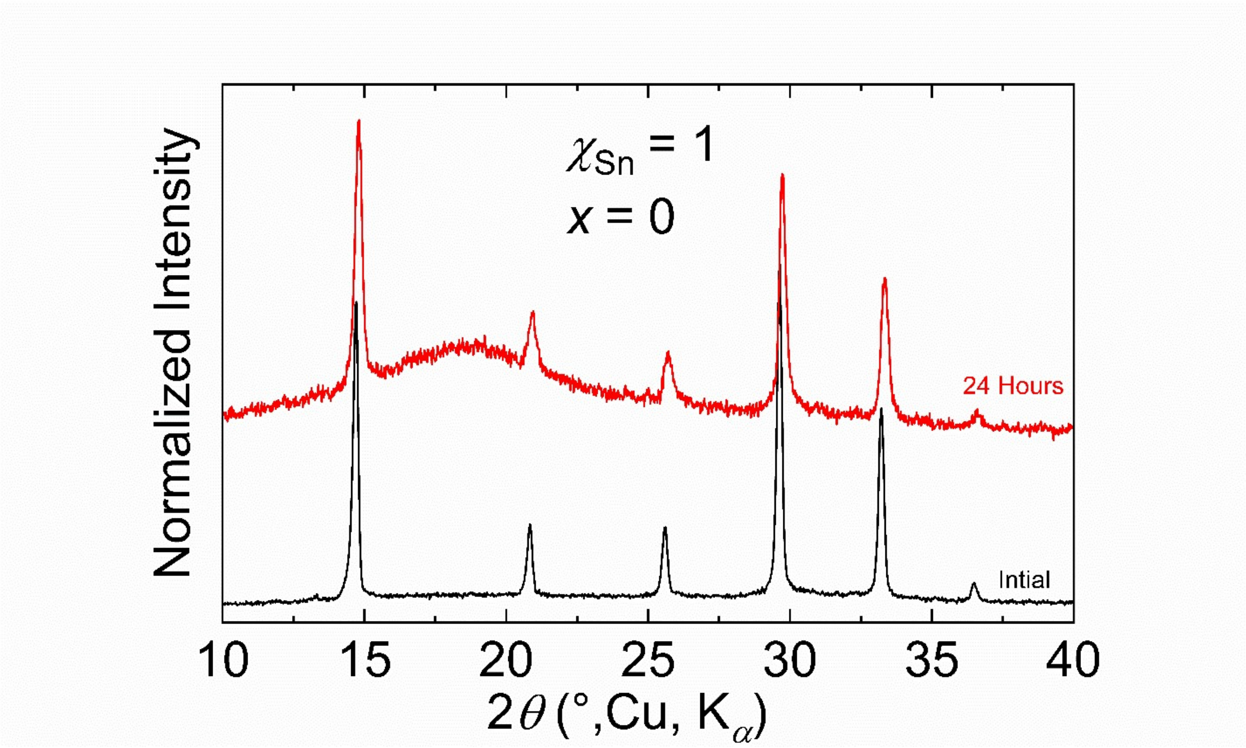

Figure S26: Experimental powder diffraction patterns for the $x = 0$, $\chi_{Sn} = 1$ compound upon intial and 24 hours of exposure to air.

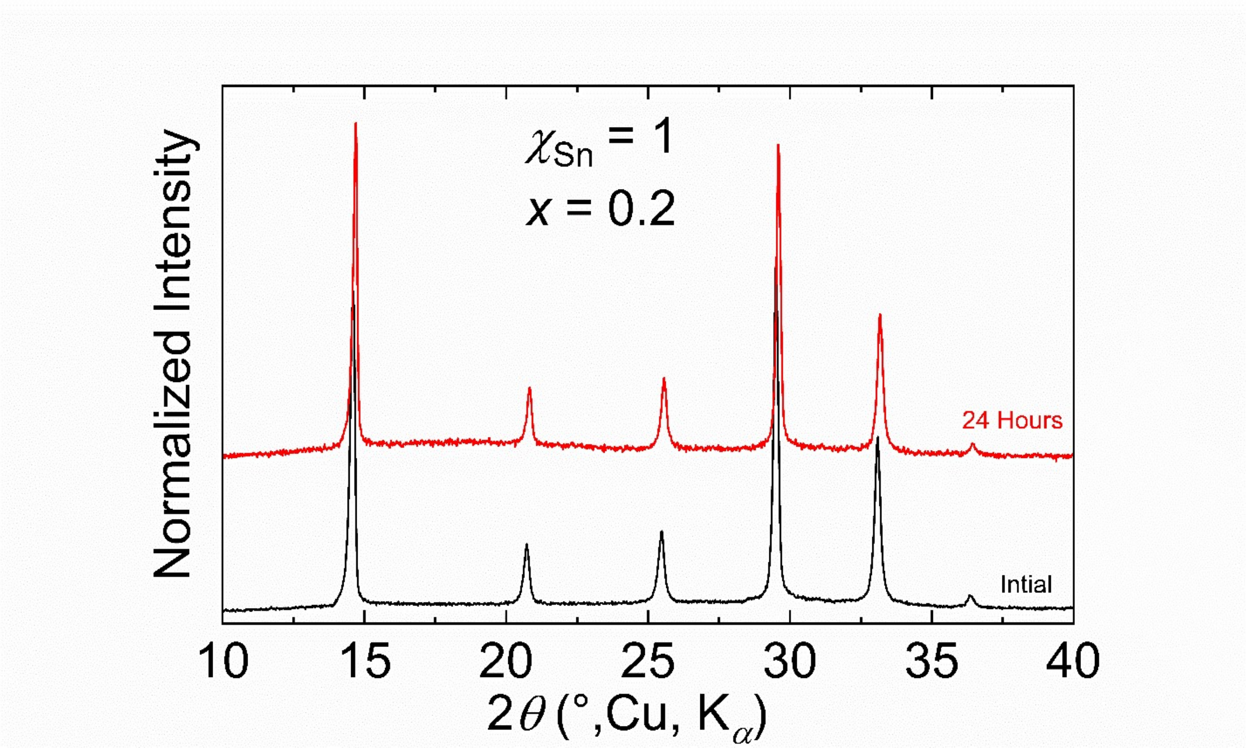

Figure S27: Experimental powder diffraction patterns for the $x = 0$, $\chi_{Sn} = 1$ compound upon intial and 24 hours of exposure to air.

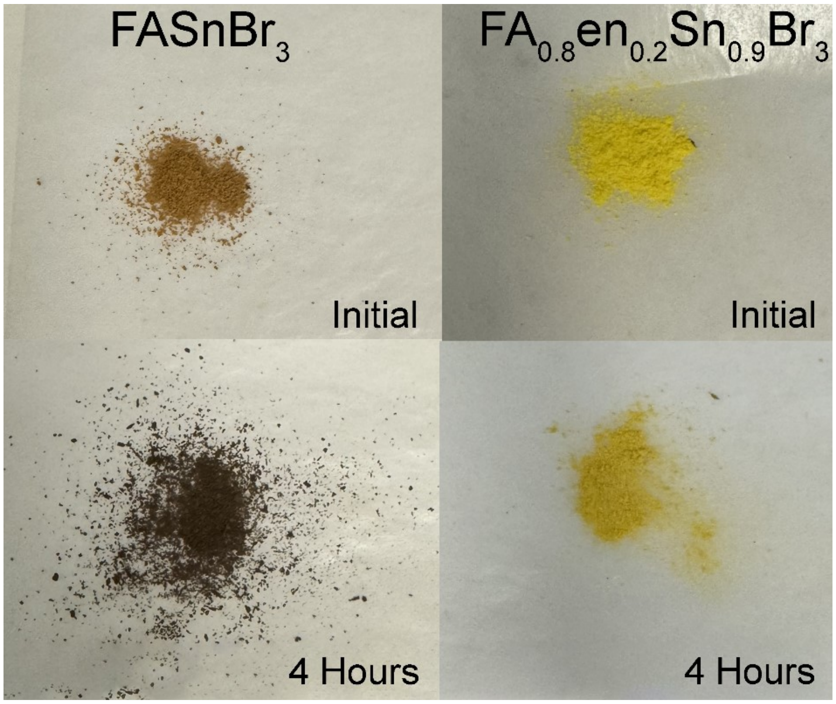

Figure S28: Images of $FASnBr_3$ and $FA_{0.8}en_{0.2}Sn_{0.9}Br_3$ powder upon initial and 4 hours of exposure to air.

Note S8: $FASnBr_3$ indially oxidizes on the surface forming SnO, darkening the material before changes in experimetnal powder diffraction patterns can be observed. Therefore, oxidation can be informed directly from alterations in the sample appearance.

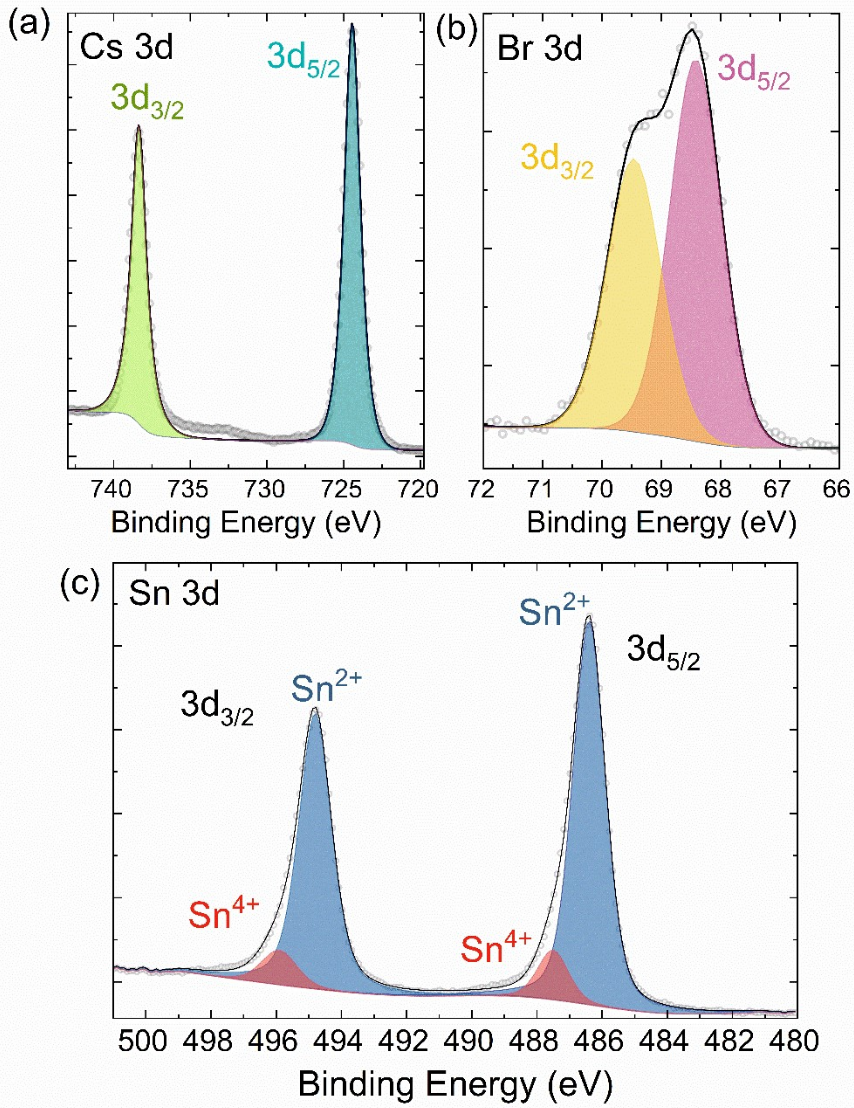

Figure S29: Extra XPS data for $CsSnBr_3$ 2+ standard. (a) Cs 3*d* spectra showing peaks consistent with Cs-Br bonding. (b) Br 3*d* spectra showing peaks consistent with Br-Sn bonding. (c) Sn 3*d* spectra with 2 components per main peak, the majority being Sn^{2+} (89%) and a minor Sn^{4+} (11%) peak.

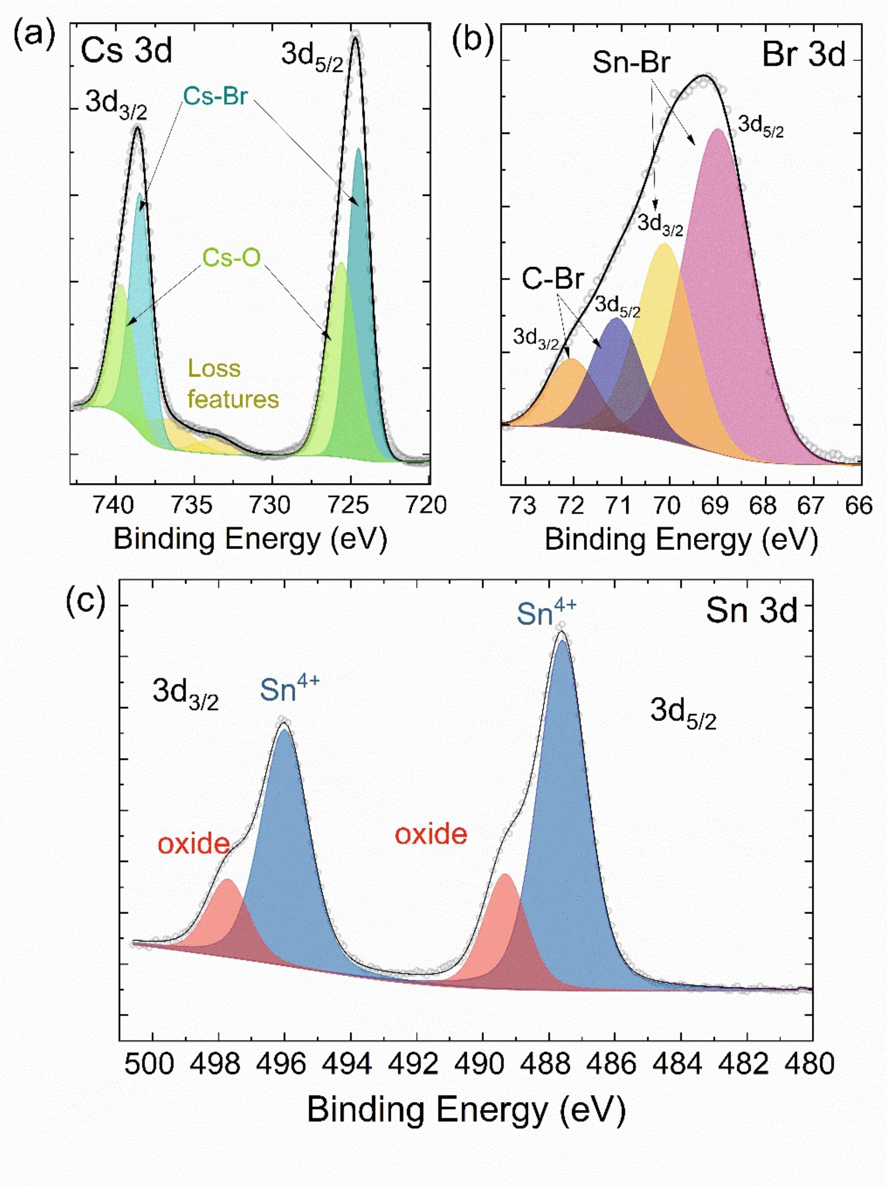

Figure S30: Extra XPS plots for Sn^{4+} standard Cs_2SnBr_6. (a) Cs 3*d* spectra showing two components per peak, the majority being Cs-Br bonding and some surface oxide. Additional loss features are visible as is typical for Cs 3*d*.[3] (b) Br 3*d* spectra showing two components per peak, the majority being Br-Sn bonding and some Br-C bonding, likely from surface contamination. (c) Sn 3*d* spectra with 2 components per main peak, the majority being Sn^{4+} from Cs_2SnBr_6 and some tin oxide. Peak positions for surface contamination species were determined using the XPS handbook.[3]

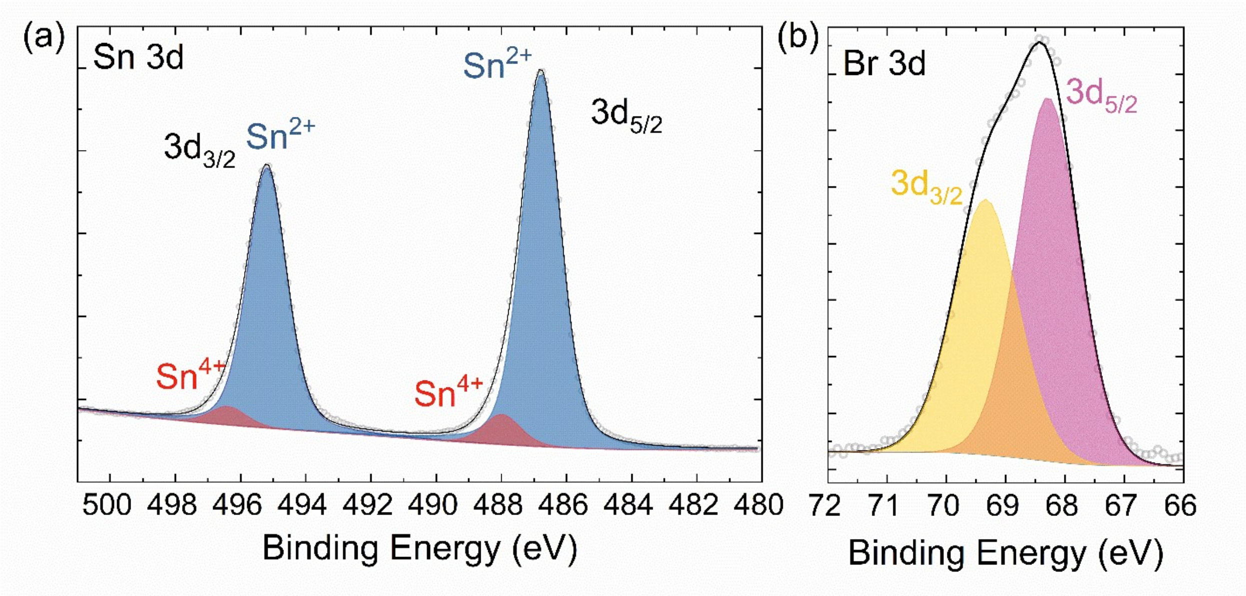

Figure S31: Extra XPS data for $FASnBr_3$ ($\chi_{Sn} = 1$, $x = 0.33$). (a) Sn 3*d* spectra with 2 components per main peak, the majority being Sn^{2+} (93%) and a minor Sn^{4+} peak (7%). (b) Cs 3*d* spectra showing peaks consistent with Cs-Br bonding.

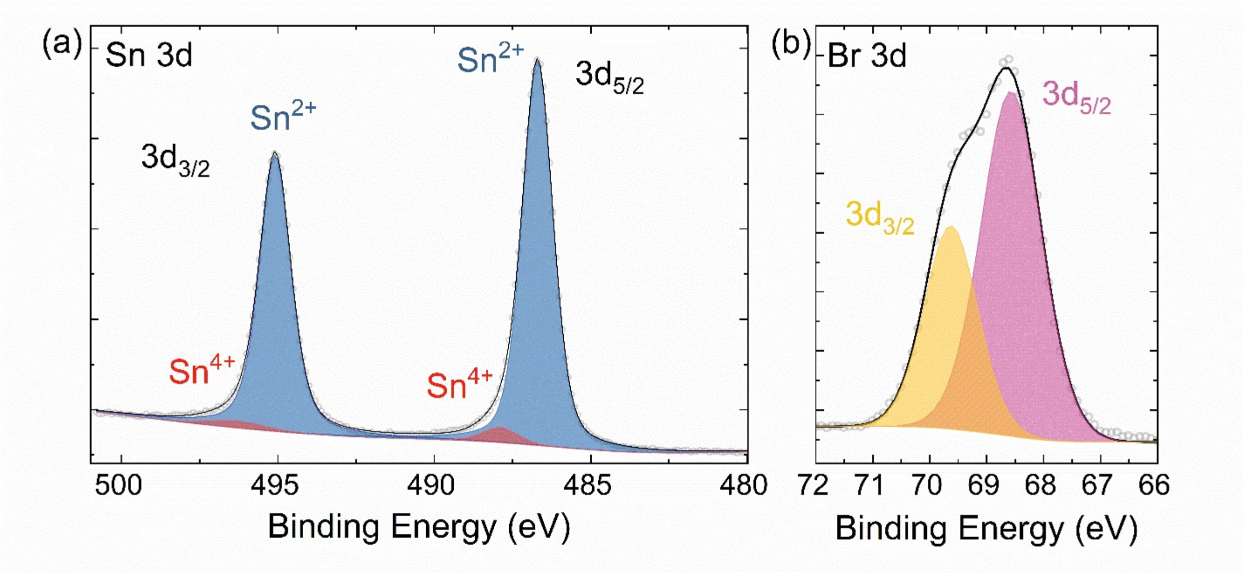

Figure S32: Extra XPS data for $FA_{0.67}en_{0.33}Sn_{0.84}Br_3$ ($\chi_{Sn} = 1$, $x = 0.33$). (a) Sn 3*d* spectra with 2 components per main peak, the majority being Sn^{2+} (96%) and a minor Sn^{4+} peak (4%). (b) Cs 3*d* spectra showing peaks consistent with Cs-Br bonding.

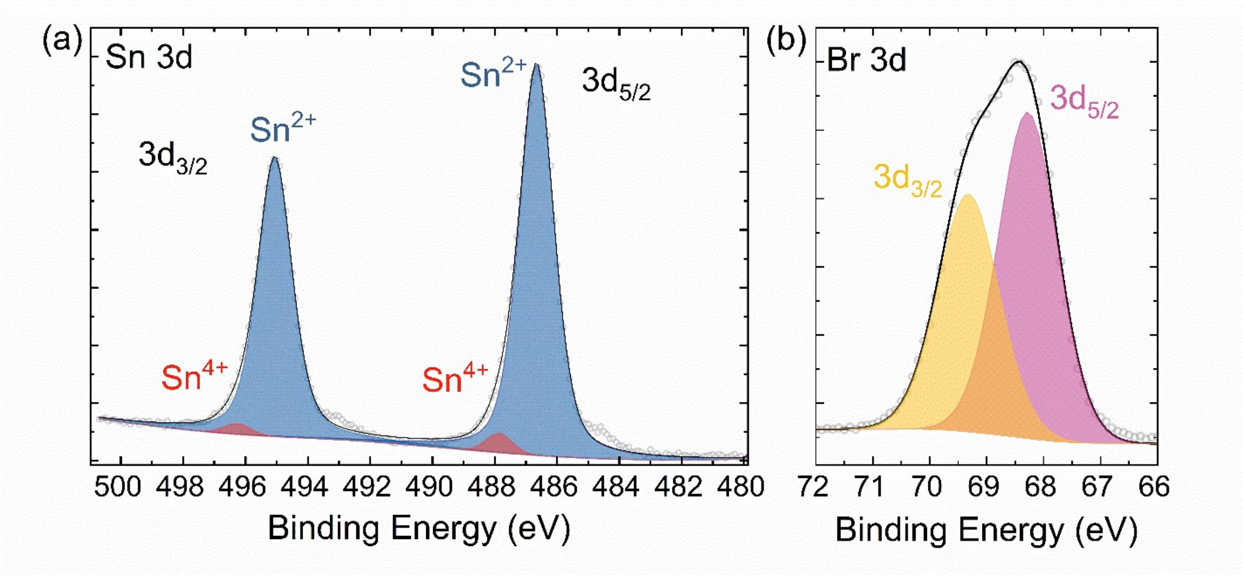

Figure S33: Extra XPS data for $FA_{0.64}en_{0.36}Pb_{0.42}Sn_{0.40}Br_3$ (χ_{Sn} = 0.5, x = 0). (a) Sn 3*d* spectra with 2 components per main peak, the majority being Sn^{2+} (88%) and a minor Sn^{4+} peak (12%). (b) Cs 3d spectra

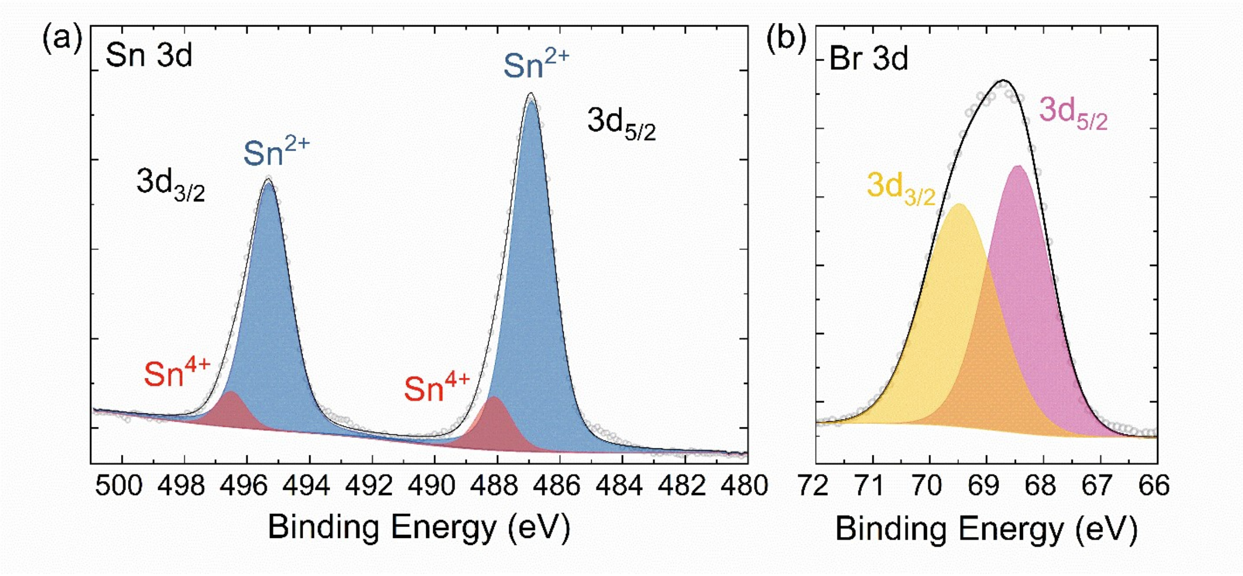

showing peaks consistent with Cs-Br bonding.

Figure S34: Extra XPS data for $FA_{0.64}en_{0.36}Pb_{0.42}Sn_{0.40}Br_3$ (χ_{Sn} = 0.5, x = 0.36). (a) Sn 3*d* spectra with 2 components per main peak, the majority being Sn^{2+} (96%) and a minor Sn^{4+} peak (4%). (b) Cs 3*d* spectra showing peaks consistent with Cs-Br bonding.

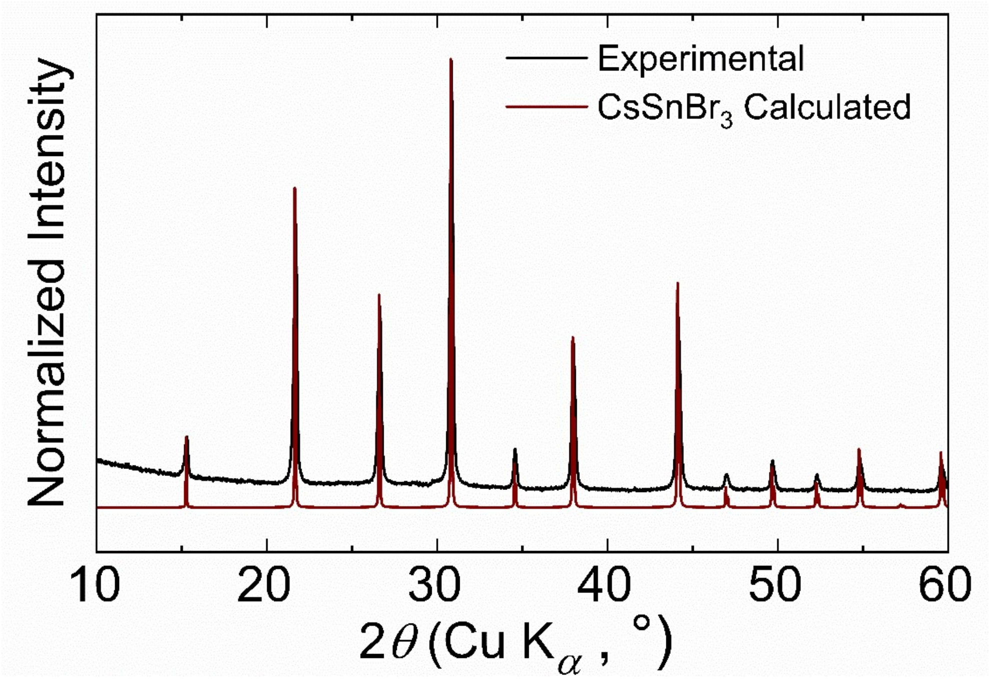

Figure S35: Experimental X-ray powder diffraction pattern for the as syntehsized $CsSnBr_3$ compared against the calculated pattern.

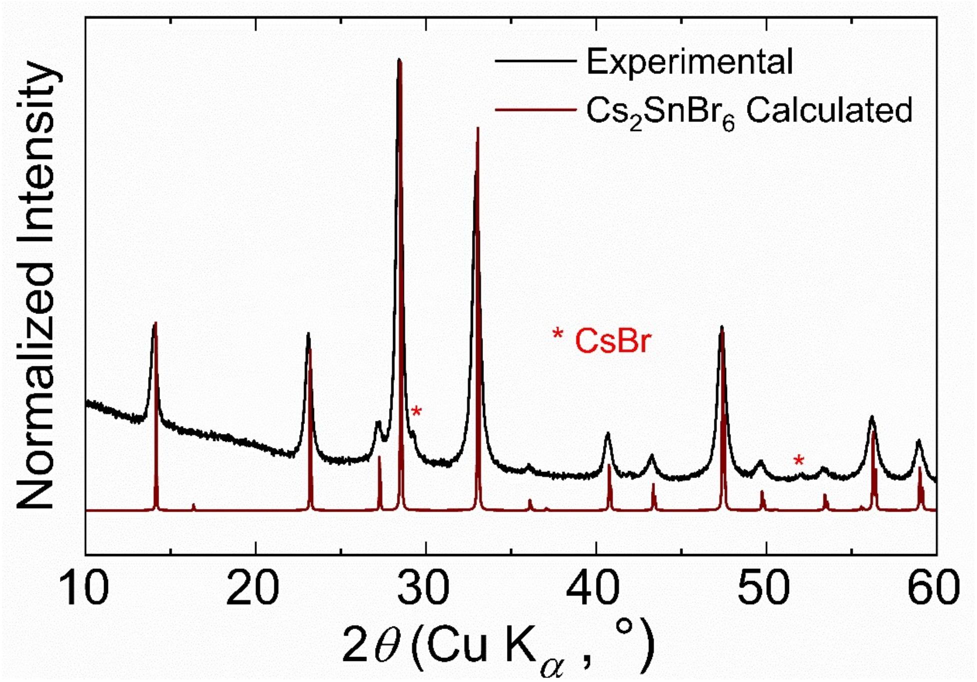

Figure S36: The experimental X-ray powder diffraction pattern for the as synthesized Cs_2SnBr_6 compared against the caluclated pattern. Peaks from unreacted CsBr are highlighted (*).

References:

1. G. Kortüm, W. Braun and G. Herzog, *Angewandte Chemie International Edition in English*, 1963, **2**, 333-341.
2. I. Spanopoulos, I. Hadar, W. Ke, P. Guo, E. M. Mozur, E. Morgan, S. Wang, D. Zheng, S. Padgaonkar, G. N. Manjunatha Reddy, E. A. Weiss, M. C. Hersam, R. Seshadri, R. D. Schaller and M. G. Kanatzidis, *Journal of the American Chemical Society*, 2021, **143**, 7069-7080.
3. Moulder, J. F. *Handbook of X-Ray Photoelectron Spectroscopy: A Reference Book of Standard Spectra for Identification and Interpretation of XPS Data*; Physical Electronics Division, Perkin-Elmer Corporation, 1992.
